# Exploring the Fecal Microbiome Dysbiosis and Its Plasma Metabolome Determinants in Advanced Parkinson's Disease With Motor Complications

**DOI:** 10.1002/cns.70750

**Published:** 2026-01-23

**Authors:** Shuangjie Qian, Jialong Hou, Xi Xiong, Qi Duan, Tao Jiang, Yi Zheng, Weiwei Quan, Jiaxue Xu, Keke Chen, Jingjing Qian, Hongchang Gao, Chenglong Xie

**Affiliations:** ^1^ Department of Neurology The First Affiliated Hospital of Wenzhou Medical University Wenzhou Zhejiang China; ^2^ School of Pharmaceutical Science Wenzhou Medical University Wenzhou Zhejiang China; ^3^ Key Laboratory of Alzheimer's Disease of Zhejiang Province, Institute of Aging Wenzhou Medical University Wenzhou Zhejiang China; ^4^ Oujiang Laboratory Wenzhou Zhejiang China; ^5^ Department of Geriatrics, Geriatric Medical Center The First Affiliated Hospital of Wenzhou Medical University Wenzhou Zhejiang China

**Keywords:** fecal microbiome, motor complications, multiomics biomarkers, Parkinson's disease, plasma metabolome

## Abstract

**Background:**

Parkinson's disease with motor complications (PD‐MC) lacks effective diagnostic and therapeutic strategies. The perturbations of the gut microbiota and plasma metabolites are closely associated with the etiopathogenesis of PD. However, whether fecal microbiome dysbiosis and changed plasma metabolites are involved in PD progression, particularly in the development of PD‐MC, is still unclear.

**Methods:**

In this study, we performed an extensive multiomics analysis involving 108 PD patients for 16S rRNA gut microbiome profiling and 246 PD patients for plasma nontargeted metabolomics. Our findings revealed distinct gut microbiota and plasma metabolites associated with PD‐MC. Utilizing these discriminative features, we developed a multivariate diagnostic model for PD‐MC. The relationships between differential metabolites and microorganisms were evaluated using Spearman correlation analysis. Functional interpretation of the key metabolites was conducted through enrichment and pathway analysis, employing the KEGG and SMPDB databases.

**Results:**

PD‐MC patients had distinct gut microbial signatures as compared with PD without motor complications (PD‐NMC) individuals and were increased in fecal Lactobacillus, Limosilactobacillus, Bifidobacterium, and Ligilactobacillus genera along with depleted Agathobacter. Moreover, metabolomic analysis revealed the differences in plasma 3‐deoxysappanchalcone (3‐DSC), 1,3‐Dimethyluracil (1,3‐DTl), Leucine, and N‐Acetylisoleucine (N‐AIL), Dodec‐6‐enoic acid (D‐6‐E), N‐butyl Oleate (N‐BO), and 4‐hydroxyundecanoic acid (4‐HUA) in PD‐MC compared to PD‐NMC. Spearman correlation analysis showed that the fecal microbiota aberrations in PD‐MC patients were linked to plasma metabolic changes, indicating the association between key microbial populations and metabolomic profiles in PD‐MC.

**Conclusions:**

This study underscores the value of employing integrated multiomics profiling of the fecal microbiome and plasma metabolome to enhance the mechanistic understanding of PD‐MC and to identify potential diagnostic biomarkers.

## Graphical abstract

Our multiomics analysis across three PD cohorts revealed distinct fecal microbiome and plasma metabolome alterations in patients with motor complications (PD‐MC). We identified specific microbial and metabolic biomarkers and uncovered significant correlations between them, providing new insights into PD‐MC pathophysiology.

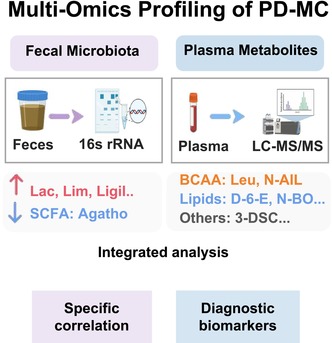

**Abbreviations**
1,3‐DTl: 1,3‐Dimethyluracil
16SrRNA: 16S ribosomal RNA
3‐DSC: 3‐deoxysappanchalcone
4‐HUA: 4‐hydroxyundecanoic acid
7‐MDCA: 7‐methyldecanoic acid
ADL: Activities of Daily Living Scale
ANOVA: analysis of variance
AUC: area under the curve
BMI: body mass index
CI: confidence interval
CSF: cerebrospinal fluid
CTAB: cetyltrimethylammonium bromide
D‐6‐E: Dodec‐6‐enoic acid
ESI: electrospray ionization
FC: fold change
FDR: false discovery rate
GLM: generalized linear model
HAMA: Hamilton Anxiety Inventory
HAMD: Hamilton Depression Rating Scale
HILIC: hydrophilic interaction liquid chromatography
HMDB: Human Metabolome Database
KEGG: Kyoto Encyclopedia of Genes and Genomes
LC–MS: liquid chromatography‐mass spectrometry
LDA: linear discriminant analysis
L‐dopa: levodopa
LEDD: Levodopa Equivalent Daily Dose
LEfSe: Linear discriminant analysis Effect Size
LSD: least significant difference
MDA: mean decrease accuracy
MDG: Mean Decrease Gini
MDS: movement disorders
MMSE: mini‐mental state examination
MPTP: 1‐methyl‐4‐phenyl‐1,2,3,6‐tetrahydropyridine
N‐AIL: N‐Acetylisoleucine
N‐BO: N‐butyl Oleate
NMDS: non‐metric multidimensional scaling
NMS: nonmotor symptoms
OOB: out‐of‐bag error
OTUs: operational taxonomic units
PCA: principal component analysis
PCoA: principal coordinate analysis
PD: Parkinson's disease
PD‐MC: PD‐motor complications
PD‐NMC: PD without motor complications
PEA: pathway enrichment analysis
PLS‐DA: partial least squares discriminant analysis
PubChem: public chemical database
QC: quality control
QIIME: quantitative insights into microbial ecology
RBDQ‐HK: The Rapid Eye Movement Sleep Behavior Disorder Questionnaire‐Hong Kong Version
RF: random forest
ROC: receiver operating characteristic
RT: retention time
SCFA: short‐chain fatty acids
SCFAs: short‐chain fatty acids
SMPDB: small molecule pathway database
SNpc: substantia nigra pars compacta
UPDRS: Unified Parkinson's Disease Rating Scale
VIP: variable importance in projection

## 1 Introduction

Parkinson's disease (PD) is a second common aging‐related progressive neurodegenerative disorder characterized by the degeneration of dopaminergic neurons within the substantia nigra pars compacta (SNpc) and a substantial decrease of dopamine level in the striatum [1]. As the exact pathogenesis of PD remains elusive, no disease‐modifying regimens are available for PD, and levodopa (L‐dopa), the amino‐acid precursor of dopamine, remains the most effective available drug for controlling PD motor symptoms [2]. Nonetheless, its effectiveness diminishes over time as the disease progresses. Higher dosage and long‐term oral L‐dopa can reduce off time but tend to increase significant motor complications, encompassing symptom fluctuations and dyskinesia [3], are significant concerns in long‐term treatment. Symptom fluctuations primarily involve wearing‐off, the on‐off phenomenon, and freezing of gait, whereas dyskinesia predominantly includes peak‐dose dyskinesia, biphasic dyskinesia, and off‐period dystonia. The pathogenesis of these complications is largely attributed to the degeneration of dopamine neurons and abnormal sensitivity of dopamine receptors. Following 3 to 5 years of levodopa therapy, approximately 50% of patients experience motor complications, with the risk markedly increasing to 80% for those treated for over 10 years [4]. Because of the PD‐related motor complications (PD‐MC), the L‐dopa is often postponed and substituted by less effective, poorly tolerated alternatives, such as amantadine or dopamine agonists [5]. Since clinically effective strategies to mitigate PD‐MC are highly restricted, novel approaches or prevention ways are needed [6]. It is therefore vital to filter those at greater risk of motor complications from PD subjects because these conditions impose a huge burden on the patients, their families, and society.

Over the past decade, the microbiota–gut–brain axis has been suggested to play an important role in PD based on studies in humans and animal models [7, 8]. A plethora of preclinical and clinical studies have shed light on the complicated relationship between the gut and the brain in PD conditions [9]. In parallel, gut dysbiosis, as an emerging biomarker and intervention target for various complex diseases, has also been consistently reported in PD patients [10]. Of note, it was reported that PD‐associated gut microbiota disturbance, especially the lack of short‐chain fatty acids (SCFA)‐producing bacteria and enrichment of putative pathobionts [11, 12], was related to intestinal immune activation, hyperpermeability, and α‐synuclein aggregation [13]. Moreover, alterations in the gut microbiome have also been displayed in several rodent models of PD, including neurotoxin MPTP, Rotenone, and 6‐OHDA‐induced models, and transgenic mice overexpressing αsynuclein [14, 15]. The relationship between gut‐related microbiota and PD pathogenesis in rodents and humans is largely correlative. However, whether fecal microbiome dysbiosis may be involved in disease progression, particularly in the development of PD‐MC, is still unclear. A recent study reported that the relative abundance of *Lachnospiraceae Blautia* and *Lactobacillaceae Lactobacillus* was significantly changed in the gut microbiota of PD patients with motor complications [16]. This indicates that an imbalance (dysbiosis) in microbiome composition and function is probably associated with the development of PD‐MC. However, although this prior study suggested differentiated variations in microbial composition in PD‐MC, it might be underpowered to comprehensively detect host–microbiome interactions.

Notably, the fecal microbiome is actively involved in the metabolism of plasma metabolites. Recent studies have shown that alterations in plasma metabolites are linked to changes in human health and various diseases, including PD [17]. Changes in the concentration of metabolites have also been widely studied for PD diagnostics within the blood metabolome, including dopamine metabolites, catecholamines, amino acids, and urate et al. [18, 19]. Until the last decade, untargeted metabolomics (such as Liquid chromatography‐mass spectrometry (LC‐MS)) has been applied to the PD field, counting on its abundant potential for the identification of novel biomarkers [20, 21]. LC‐MS is a powerful tool to profile metabolite changes and has been utilized to study plenty of biofluids concerning PD prognosis and diagnosis, such as blood, saliva, and cerebrospinal fluid (CSF) [22]. A previous study indicated the involvement of amino acids, acylcarnitines, organic acids, steroids, amides, and lipids in PD participants [20]. However, whether plasma metabolites are altered in advanced PD‐MC patients remains undetermined. Interestingly, combining fecal microbiome and plasma metabolome presents a promising approach for understanding the development of PD‐MC and related gut environment alterations and offers a noninvasive biomarker for PD patients with motor complications. In this work, we comprehensively analyzed the fecal microbiome dysbiosis and its plasma metabolome determinants of PD patients from three independent cohorts. Our objective was to identify specific gut bacteria and plasma metabolites that contribute to the development of PD‐MC. We also aimed to create prediction models using disease‐specific multiomics biomarkers to enhance PD‐MC prediction.

## 2 Results

### 2.1 Sociodemographic and Comprehensive Multiomics Characterization of PD‐MC and PD‐NMC

**FIGURE 1:**
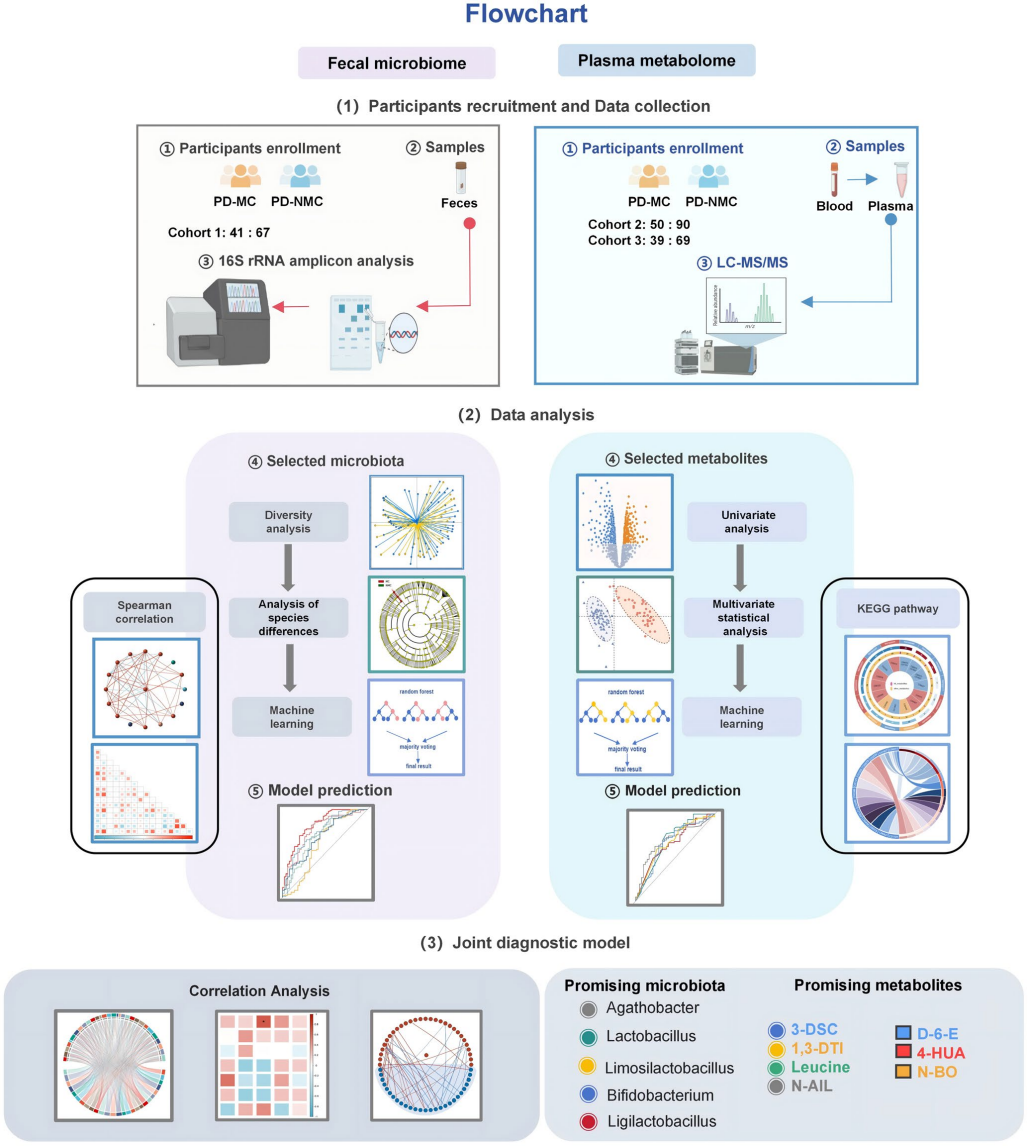
Flowchart illustrating the analysis of the fecal microbiome and plasma metabolome. The figure delineates the grouping, sample collection, fecal 16S rRNA amplicon analysis, plasma nontargeted Liquid Chromatography‐Mass Spectrometry (LC–MS) metabolomics analysis, and the integrated analysis of characteristic microbiota and metabolites across the three cohorts, culminating in the identification of five significant microbial genera and seven core metabolites.

In this study, we employed a multiomics approach that integrates fecal 16S rRNA and plasma metabolomics to investigate alterations in PD‐MC subjects. Table 1 shows important demographic data for each group investigated here and the results of significance tests between cohort group metadata. A total of 354 individual PD participants were recruited from the First Affiliated Hospital of Wenzhou Medical University, for whom we obtained clinical and multiomics profiling and divided into 2 independent sections: (i) Cohort 1 for the microbiome, including 42 PD‐MC and 66 PD‐NMC; and (ii) Metabolome cohort, including 87 PD‐MC and 159 PD‐NMC (Figure 1). In terms of the basic characteristics, there were no statistically significant differences in age, sex ratio, BMI, smoking, hypertension, and diabetes mellitus between PD‐MC and PD‐NMC both in the microbiome and metabolome dataset, which suggested that the participants in each group were comparable. Notably, we aim to reveal the patterns of variation in fecal microbiota and plasma metabolites through a comprehensive statistical analysis, and then utilize machine learning techniques for diagnosing and predicting PD‐MC.

**TABLE 1:** Demographic characteristics of the gut microbiome and plasma metabolomics cohorts.

Characteristics	Cohort for microbiota			Cohort for metabolome		
	PD‐MC	PD‐NMC	*p* value^a^	PD‐MC	PD‐NMC	*p* value^a^
	(*N* = 41)	(*N* = 67)		(*N* = 87)	(*N* = 159)	
Age, years	68.9 ± 8.5	67.0 ± 10.0	0.315	67.8 ± 8.0	65.2 ± 11.1	0.035
Sex (Females%)^b^	20 (48.8%)	30 (44.8%)	0.685	43 (49.4%)	70 (44%)	0.416
BMI, kg/m^2^	24.5 ± 3.1	25.4 ± 3.1	0.151	23.8 ± 4.3	23.8 ± 3.1	0.957
Education, years	3 (0, 6)	4 (0, 6.5)	0.941	2 (0, 6)	5 (0, 7)	0.01
Disease duration, years	7 (4.3, 10)	3 (1, 5)	< 0.001	7 (5, 10)	2 (1, 3)	< 0.001
Cigarette, *n* (%)	5 (12.2%)	17 (25.4%)	0.099	15 (17.2%)	33 (20.8%)	0.521
Alcohol, *n* (%)	5 (12.2%)	20 (29.9%)	0.035	14 (16.1%)	49 (30.8%)	0.012
Hypertension, *n* (%)	16 (39%)	34 (50.7%)	0.236	29 (33.3%)	54 (34%)	0.921
Diabetes, *n* (%)	11 (26.8%)	15 (22.4%)	0.551	15 (17.2%)	25 (15.7%)	0.758
Constipation, *n* (%)	33 (80.5%)	34 (50.7%)	< 0.001	65 (74.4%)	56 (35.2%)	< 0.001
Dyskinesia, *n* (%)	14 (35%)	0 (0%)	0.233	20 (23%)	0 (0%)	< 0.001
Fall, *n* (%)	17 (42.5%)	10 (14.9%)	< 0.001	32 (36.8%)	21 (13.3%)	< 0.001
UPDRS total score	54.5 (34, 72)	39 (28, 50)	0.005	55 (44, 77)	35 (22, 48)	< 0.001
UPDRS Part I score	2 (1, 4)	1 (0, 3)	0.127	2 (1, 5)	2 (1, 3)	0.013
UPDRS Part II score	14.5 (10, 20.3)	10 (8, 13)	< 0.001	16 (12, 22)	9 (6, 13)	< 0.001
UPDRS Part III score	33 (21, 43.5)	27 (17, 33)	0.069	34 (22, 43)	23 (14, 32)	< 0.001
UPDRS Part IV score	3 (2, 6.3)	0 (0, 1)	< 0.001	5 (3, 7)	0 (0, 2)	< 0.001
H‐Y stage	3 (2, 4)	2 (1.5, 3)	< 0.001	3 (2.5, 4)	2 (1.5, 3)	< 0.001
MMSE	19.8 ± 5.6	20.9 ± 6.4	0.396	18.8 ± 6.9	21.5 ± 6.5	0.002
HAMA	9 (5, 13)	6 (3, 9)	0.009	9.5 (5, 13.3)	8 (4, 11)	0.027
HAMD	6 (4, 11)	6 (2.8, 9)	0.010	6.5 (4, 11)	5 (2, 8)	< 0.001
RBDQ‐HK	14 (6.3, 28.5)	6.5 (3, 21)	0.038	22 (6.8, 41)	9 (3, 23)	< 0.001
ADL	36 (27.5, 49)	28 (22, 35)	< 0.001	35 (28.8, 48)	25 (20, 31)	< 0.001
PD medication, *n* (%)	41 (100%)	57 (85.1%)	0.012	87 (100%)	133 (83.6%)	< 0.001
L‐DOPA intake, *n* (%)	35 (85.8%)	55 (82.1%)	0.550	82 (94.3%)	129 (81.1%)	0.005
Dopamine agonists, *n* (%)	28 (68.3%)	38 (56.7%)	0.171	69 (79.3%)	84 (52.8%)	< 0.001
MAO‐B inhibitor, *n* (%)	10 (24.4%)	16 (23.9%)	0.952	17 (19.5%)	28 (17.6%)	0.708
COMT inhibitor, *n* (%)	18 (43.9%)	4 (6%)	< 0.001	40 (40.6%)	19 (11.9%)	< 0.001
Amantadine, *n* (%)	10 (24.4%)	5 (7.5%)	0.014	22 (25.3%)	24 (15.1%)	< 0.001
Benzhexol hydrochloride	0 (0%)	0 (0%)	NA	5 (5.7%)	6 (3.8%)	0.262
LEED mg/day	606 (381.5, 802.8)	375 (187.5, 475)	< 0.001	550 (375, 715)	338 (150, 413)	< 0.001

*Note:* Continuous variables were evaluated for normality using the Kolmogorov–Smirnov test. Data are presented as mean (standard deviation) for normally distributed variables, median (interquartile range) for non‐normally distributed variables, and as *n* (%) for categorical variables. The *p* values for continuous variables, including Age, BMI, and MMSE, were derived from the Independent Samples Student's *t*‐test for normally distributed data, or the Mann–Whitney *U* test for data not following a normal distribution. For categorical variables, *p* values were calculated using the chi‐squared test.

Abbreviations: ADL: Activity of Daily Living Scale; BMI, body mass index; HAMA, Hamilton Anxiety Scale; HAMD, Hamilton Depression Scale; LEED, Levodopa equivalent daily dose; MC, Motor complications; MMSE, Mini‐Mental State Examination; NMC, nonmotor complications; PD, Parkinson's disease; RBDQ‐HK, REM sleep behavior disorder questionnaire‐Hong Kong; UPDRS, unified Parkinson's disease rating scale.

^a^ The *p* values associated with the two groups.

^b^ The number of individuals and the percentage within each group.

We obtained omics data, using identical methodologies and platforms in each group, which we further sampled for individuals matching baseline characteristics. Moreover, PD‐MC was characterized by longer disease duration compared to PD‐NMC, with a *p*‐value of < 0.001, indicating that disease duration is the vital prediction factor of motor complications. To investigate a potential association between neuropsychological severity and motor complications, our analysis revealed that the UPDRS scores were significantly elevated in the PD‐MC compared with the PD‐NMC group. Moreover, the PD‐MC group exhibited moderately higher HAMA, HAMD, RBD‐HK, and ADL scores compared with the PD‐NMC and lower MMSE scores, suggesting the worse neuropsychological functions were related to an increased likelihood of PD‐MC patients. According to the medication data (such as the levodopa equivalent dose (LEDD)), we confirmed the associations between PD‐MC with medication factors. Finally, we utilized a series of differential analyses and feature filtration to identify 5 microbiota and 7 metabolites that effectively discriminate PD‐MC patients (Figure 1). To explore gut microbiota‐related plasma metabolic processes, we introduced the multiomics biological correlation maps framework to explore the connections.

### 2.2 Microbiota in PD‐MC and PD‐NMC

**FIGURE 2:**
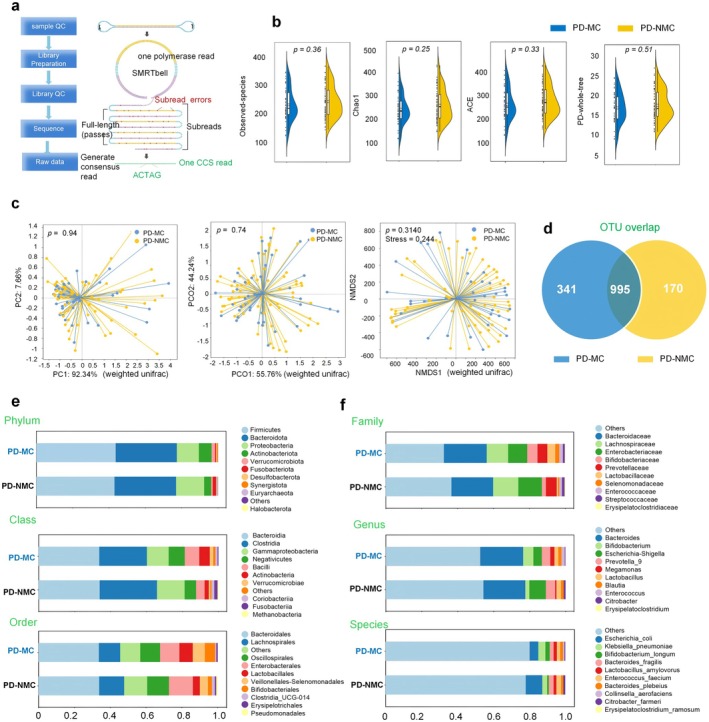
Abundance and diversity of microbiota associated with PD‐MC and PD‐NMC. (a) Mechanism diagram of 16S rRNA amplicon analysis; (b) Alpha diversity analysis encompassed the Observed‐species, Chao1 index, ACE index, and PD‐whole‐tree (Wilcoxon rank‐sum test); (c) Beta diversity was assessed using Principal component analysis (PCA), Principal coordinate analysis (PCoA) and nonmetric Multidimensional scaling (NMDS) analysis based on weighted unifrac. A stress value of less than 0.1 represents a high‐quality ordination. Statistical differences were evaluated using Wilcoxon methods, with a *p* value of less than 0.05 deemed statistically significant. (d) Venn diagram based on microbial Operational Taxonomic Units (OTUs). (e, f) Bar plot of top 10 abundant species across the taxonomic levels of the phylum, class, order, family, genus, and species levels.

We then characterized the fecal microbiota associated with PD‐MC through high‐throughput sequencing of the V_3_–V_4_ region of the 16S rRNA gene (Figure 2a). A total of 108 samples were subjected to 16S rRNA analysis utilizing the NovaSeq X Plus sequencing platform. First, we measured the bacterial richness within each sample from both groups using alpha and beta diversities. All the basic methods used to analyze the diversities were calculated based on the rooted phylogenetic tree [23]. Alpha diversity measures (e.g., observed OTUs, Chao, ACE, and PD‐whole‐tree) presented in Figure 2b indicated a low abundance of observed richness and diversity in the PD‐NMC compared to the samples of the PD‐MC at all analyzed taxonomic levels but no statistical difference was achieved (*p* = 0.36 for the observed species diversity index, *p* = 0.25 for the Chao1 diversity, *p* = 0.33 for the ACE, and *p* = 0.51 for the PD‐whole‐tree). Similarly, we calculated the beta‐diversity of the samples using the principal component analysis (PCA), principal coordinates analysis (PCoA), and NMDS ordination analysis also did not show an obvious separation of bacterial diversity between PD‐MC and PD‐NMC (Figure 2c), indicating there was little variation within these two groups. This was reasonable in a certain sense, as the PD‐MC and PD‐NMC we recruited are all from the PD populations. Interestingly, although diversity analysis showed no statistical discrepancies, the standalone number of OTUs obtained shown by the Venn diagram was 341 in the PD‐MC group and 170 in the PD‐NMC group, with 995 in common (Figure 2d), displaying the gut microbiota from patients with PD‐MC was more diverse in trends than those from the PD‐NMC subjects.

Differential taxa were assessed using the Kruskal–Wallis test at which we identified several phyla and genera significantly differed between groups. Of note, concerning phylum taxonomic comparison, a higher abundance of *Actinobacteriota* (7.0% in PD‐MC versus 3.9% in PD‐NMC) and *Verrucomicrobiota* (1.9% in PD‐MC versus 0.85% in PD‐NMC) was observed in the PD‐MC compared with PD‐NMC. However, only the decrease of *Proteobacteria* (12.1% in PD‐MC versus 15.4% in PD‐NMC) and *Fusobacteriota* (0.56% in PD‐MC versus 1.80% in PD‐NMC) in PD‐MC was significant (Figure 2e). Genus comparison discovered that PD‐MC showed a lower abundance of *Escherichia‐Shigel*, *Prevotella_9*, *Blautia*, and *Citrobacter* compared to the PD‐NMC group. Moreover, the PD‐MC was characterized by a higher abundance of *Bifidobacterium*, *Megamonas*, *Lactobacillus*, and *Enterococcus* (Figure 2f and Table S1).

### 2.3 Shifted Gut Microbiota Composition With the Progression of Motor Complications

**FIGURE 3:**
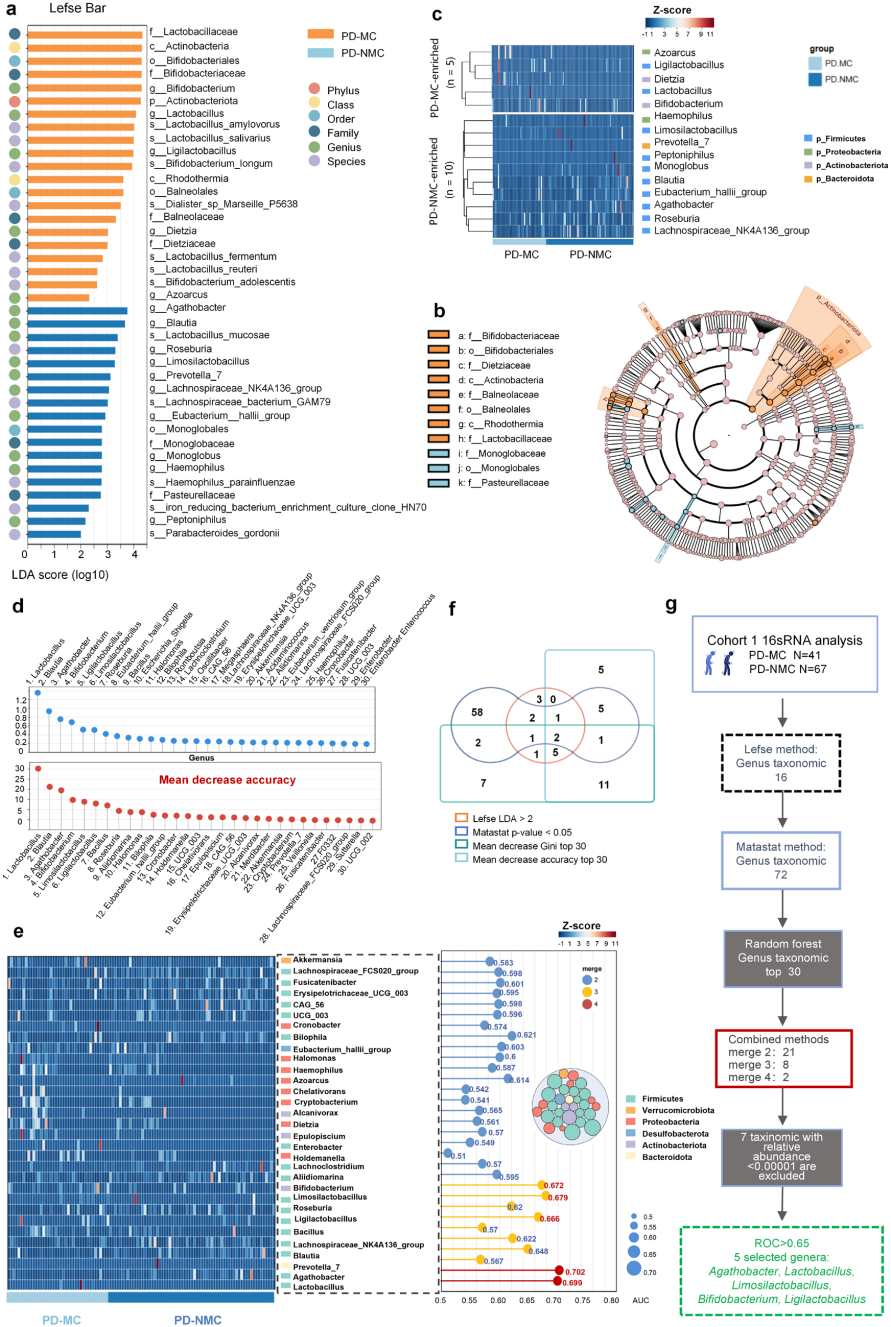
Identification of microbiota linked to PD‐MC. (a) Linear discriminant effect size (LEfSe) analysis and linear discriminant analysis (LDA) of microbiome differences between two groups at different taxonomic levels. The histogram length represents the contribution of the differential species (LDA score), and only microbial taxa that meet the LDA > 2 and the *p*‐value < 0.05 are shown in the histogram. (b) The LEfSe cladogram illustrates the differential abundance of taxa, with the innermost to outermost rings corresponding to the hierarchical levels of phylum, class, order, family, genus, and species. (c) The heat map shows the distribution of 15 genera, with 5 enriched in PD‐MC and 10 in PD‐NMC, highlighting significant abundance differences between the two groups as identified by Lefse analysis. (d) Random Forest of microbiota in PD‐MC and PD‐NMC on genus taxonomic. The lollipop plot illustrates the top 30 microbiota based on the mean decrease in accuracy and the mean decrease in the Gini index, depicted in red and blue, respectively. (f) Venn diagrams show results from random forest (accuracy and Gini index), Lefse, and metastat analyses. Taxonomic microbiota at the genus level, meeting at least two criteria from the random forest (top 30 accuracy and Gini index reductions), Lefse analysis (LDA > 2), and matastat analysis (*p* < 0.05), were identified as characteristic and were displayed in (e). (e) The lollipop chart illustrated the Area Under the Curve (AUC) values for 31 characteristic microorganisms on genus taxonomic that satisfied more than two screening criteria, with red, yellow, and blue colors indicating four, three, and two screening conditions, respectively. The circle stacking diagram illustrated the phylus distribution of these 31 microbiota at the genus taxonomic level, with the color of each circle indicating the phylus level and its size representing the AUC value. Furthermore, the left heat map presented the relative species abundance of the 31 key microorganisms in both PD‐MC and PD‐NMC. (g) Flowchart for the identification of characteristic microbiota on genus taxonomic. In Cohort 1 (PD‐MC: 41; PD‐NMC: 67), a genus‐level matastat analysis (*p* < 0.05) identified 72 microbiota. LEfSe analysis found 16, and random forest analysis selected the top 30. A Venn diagram helped identify microorganisms meeting at least two criteria. After calculating AUC values and excluding those with a relative abundance of < 0.00001, we selected 5 genera with AUC values > 0.65.

A supervised comparison of the microbiota between PD‐MC and PD‐NMC groups was carried out by linear discriminant analysis (LDA) effect size (LefSe) analysis without any adjustments, which is often utilized to identify the presence and effect size of region‐specific OTUs among different comparisons [24]. We used a logarithmic LDA score cutoff of 2.0 to screen vital taxonomic differences between the PD‐MC and PD‐NMC and revealed a notable difference in the fecal microbiome based on LefSe analysis (Figure 3a,b and Table S2). We observed that the relative abundances of the *Bifidobacterium*, *Lactobacillus*, *Ligilactobacillus*, *Dietzia*, and *Azoarcus* genera were markedly higher in the PD‐MC than in the PD‐NMC. In contrast, the relative richnesses of *Agathobacter*, *Blautia*, *Roseburia*, *Limosilactobacillus*, *Prevotella_7*, *Lachnospiraceae*, *Eubacterium*, *Monoglobus*, *Haemophilus*, and *Peptoniphilus* genera were lower in patients with PD‐MC than in PD‐NMC (Figure 3c and Table S3). To be rigorous, we identified the top 30 genera when using Mean Decrease Gini (MDG) and mean decrease accuracy (MDA) analysis, respectively (Figure 3d and Tables S4, S5), displaying higher variable importance within the PD‐MC populations relative to the PD‐NMC. Additionally, ROC‐AUC (Receiver Operating Characteristic—Area Under the Curve) provides a precise approach to evaluate the predictive accuracy of binary classification models and discover the underlying genera. As shown in Figure 3e, the highest AUC levels for 30 fecal genera were detected through this method (Table S6). To establish an optimal combination of genera signature biomarkers for the classification of PD‐MC, we analyzed the intersection of differentially abundant genera by integrating Lefse LDA > 2, Matastat *q*‐value < 0.05, MDG top 30, and MDA top 30 (at least met two methods). In general, this approach ultimately identified 5 genera that significantly contributed to distinguishing between PD‐MC and PD‐NMC (AUC > 0.65), including *Agathobacter*, *Lactobacillus*, *Limosilactobacillus*, *Bifidobacterium*, and *Ligilactobacillus* (Figure 3e,f). Furthermore, a subgroup analysis was conducted on 42 individuals diagnosed with PD‐MC, categorized into the dyskinesia group (*N* = 5), the symptom fluctuation group (*N* = 27), and the combined group (*N* = 9). A one‐way analysis of variance (ANOVA) was utilized to assess the relative abundances of the aforementioned characteristic genera. The significant difference in the abundance of *Limosilactobacillus* was observed among the three groups (*p* value = 0.028), with the highest in the dyskinesia group (Table S18).

### 2.4 Fecal Microbiota as a Potential Diagnostic Biomarker for PD With Motor Complications

**FIGURE 4:**
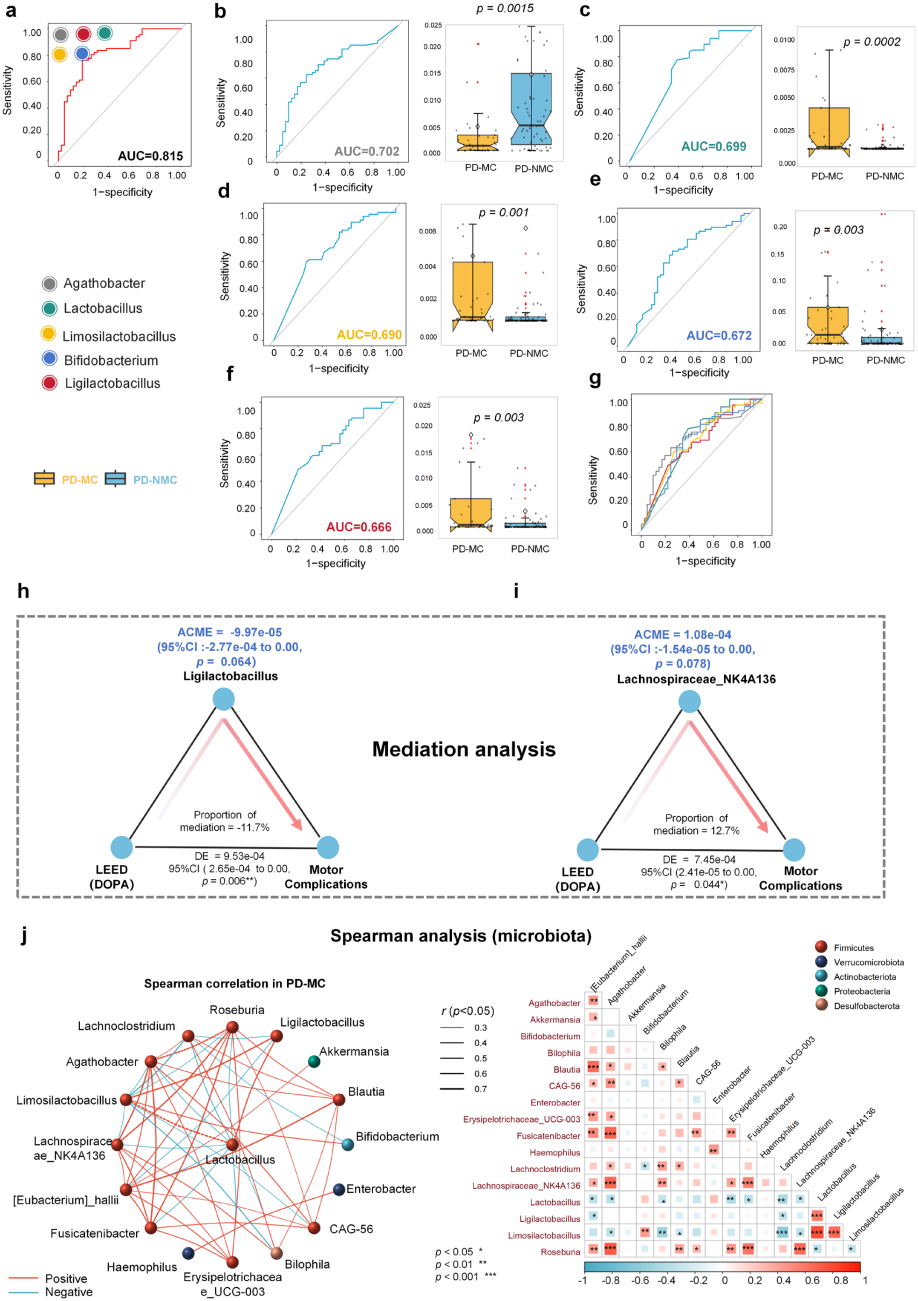
ROC curves, mediating analysis, and microbiota correlation analysis of the core microbiota in PD‐MC. (a–g) The receiver operating characteristic (ROC) curves and abundance expression level box plots for core microbiota are presented. The *p*‐values and AUC values have been annotated, where (b–f) represent Agathobacter (AUC = 0.702), Lactobacillus (AUC = 0.699), Limosilactobacillus (AUC = 0.690), Bifidobacterium (AUC = 0.672) and Ligilactobacillus (AUC = 0.666), respectively and combined AUC analysis of the five core metabolites is illustrated in (a). (h–i) The mediation analysis, adjusted for variables including age, sex, education, body mass index (BMI), and disease duration indicates that the Levodopa Equivalent Dose (LEED), which is pharmacologically equivalent to 100 mg of standard levodopa tablets, exhibits a partial but statistically nonsignificant mediating effect on motor complication via the microbiota, specifically g_Ligilactobacillus and g_Lachnospiraceae_NK4A136_group. In this context, ACME represents the Indirect Effect, while ADE denotes the Direct Effect. The mediation analysis was conducted utilizing the “mediation” package in R, employing 1000 simulation iterations (*p < 0.05, **p < 0.01, ***p < 0.001). (j) Spearman correlation analysis of the characteristic microbial abundances, revealed a significant association among the microbiota. The phylus levels are represented by nodes of different colors, while the edges indicated positive (red) or negative (blue) correlations. The thickness of the edges corresponds to the magnitude of the correlation coefficients; Correlations with coefficients greater than 0.7 are denoted by dashed lines. All correlation *p* values are below 0.05. Detailed correlation coefficients and *p*‐values are provided in heat map.

We examined the AUC values of these genera to investigate if the filtrated genera could improve the diagnostic accuracy of PD‐MC when compared to the PD‐NMC. Using a logistic regression classifier to select predictive features, we could discriminate PD‐MC from PD‐NMC with a cross‐validated AUC of 0.815 using five different genera together (Figures 4a and S1), this corresponds to a sensitivity of 74.5% and a specificity of 89.2%, indicating fecal microbiota‐based classifier showed good performance in distinguishing PD‐MC from PD‐NMC. Among them, *Agathobacter* was selected as the most important genus, singularly predicting PD‐MC with an AUC of 0.702 (sensitivity = 74.2%, specificity = 57.1%, *p* value = 0.0015). Moreover, the AUC values of the remaining genera were 0.699 (*Lactobacillus*), 0.690 (*Limosilactobacillus*), 0.672 (*Bifidobacterium*), and 0.666 (*Ligilactobacillus*), respectively (Figure 4b–g).

Given the important influence of pharmaceuticals on motor complications, we tested the effects of the dopaminergic medications (LEDD) on the microbiota. Then, to further explore whether microbiota mediated the relationships of dopaminergic medications with PD‐MC. The model was built by using the occurrence of PD‐MC as the outcome, the different types of medications, and microbiota as the exposure and mediator, respectively. The results demonstrated that the relationship between LEDD and PD‐MC was mediated by *Ligilactobacillus* genera with an approximate proportion of mediation of −11.7% (*p* = 0.006) when using mediation analyses (Figure 4h). Consistent with these results, our findings showed that the association between LEDD and PD‐MC was also partially mediated by *Lachnospiraceae_NK4A136* (Figure 4i, *p* = 0.044), pointing the potential direction of causality from gut dysbiosis, and dopaminergic medications to PD‐MC.

In light of the considerable impact of NMS, disease progression, and pharmacological treatments on PD‐MC, we reanalyzed the five different genera using a generalized linear mixed model, accounting for these factors, with PD‐MC as the outcome. The findings indicated that NMS like constipation, HAMA, and HAMD had no significant impact, while RBD significantly affected the results (*p* = 0.03). Importantly, Ligilactobacillus exhibited an independent protective effect against PD‐MC (*p* = 0.022), while Limosilactobacillus demonstrated an independent adverse effect on PD‐MC (*p* = 0.016). The analysis of interaction effects corroborated these findings, revealing significant interaction effects for both Ligilactobacillus (*p* = 0.026) and Limosilactobacillus (*p* = 0.022) with LEED. Furthermore, the significant interaction effect between Limosilactobacillus and RBD (*p* = 0.011) aligned with these results, thereby reinforcing the hypothesis that the five core genera can independently influence PD‐MC (Table S19). Then, correlations between taxa abundance were analyzed by applying the Spearman correlation network diagram. Notably, there was a significant positive correlation between the genera *Lactobacillus* abundance and *Limosilactobacillus* (*r* = 0.777, *p* = 2.27e‐09) and *Ligilactobacillus* (*r* = 0.584, *p* = 6.02e‐05) in PD‐MC (Figure 4j and Table S7). *Bifidobacterium* was positively associated with *Limosilactobacillus* and negatively related to *Lachnoclostridium*. Moreover, the genera *Agathobacter* was negatively correlated with *Lactobacillus* and *Limosilactobacillus*. Compared with PD‐MC, the association in PD‐NMC was weakened. We found there was no significant relationship between the genera *Agathobacter* and *Lactobacillus* enriched in PD‐NMC. *Lactobacillus* and *Limosilactobacillus* had a positive association, and *Bifidobacterium* and *Agathobacter* showed a negative association (Figure S2 and Table S8).

### 2.5 Patients With PD‐MC Have a Distinct Plasma Metabolite Profile

**FIGURE 5:**
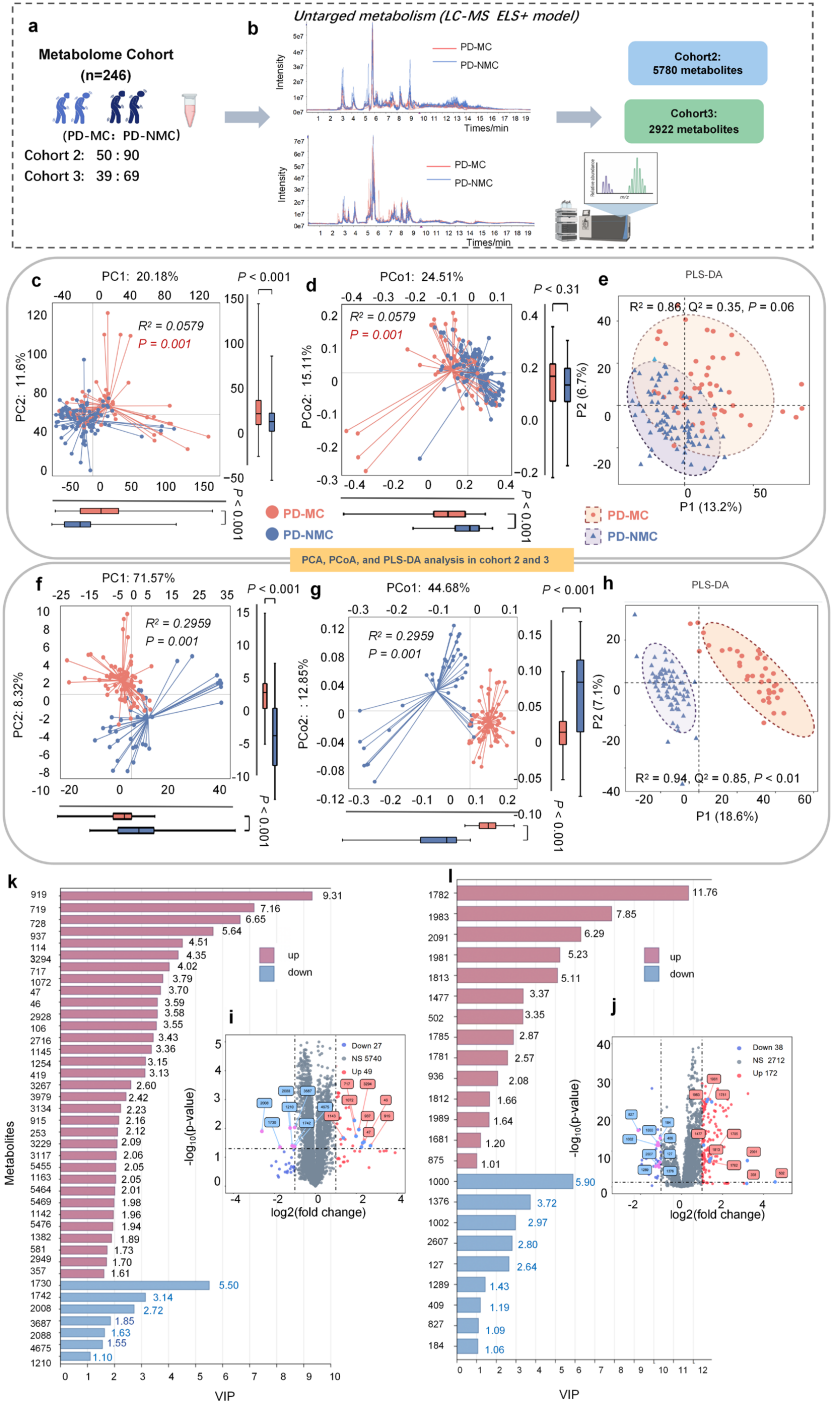
Plasma metabonomic profiling by LC–MS/MS in Cohort 2 and Cohort 3. (a, b) The metabolomics study was divided into two cohorts: Cohort 2, comprising 50 PD‐MC and 90 PD‐NMC, and Cohort 3, consisting of 39 PD‐MC and 69 PD‐NMC. Utilizing untargeted liquid chromatography‐mass spectrometry (LC–MS) in positive electrospray ionization mode (ESI+), a total of 5780 metabolites were identified in Cohort 2, while 2922 metabolites were detected in Cohort 3. (c–h) Principal component analysis (PCA), Principal coordinate analysis (PCoA) and Partial least squares discriminant analysis (PLS‐DA) analysis of metabolomic profiles in Cohort 2 and Cohort 3, with red dots correspond to PD‐MC, and blue dots correspond to PD‐NMC. Q2 indicates the predictive capability of the PLS‐DA model, while R2 denotes the fit of the model. Group differences at the principal component or coordinate level are evaluated using the two‐sided Mann–Whitney *U* test, with the results illustrated through box plots. *p* < 0.05 represents a significant difference. (k, l) The bar graph highlights key PLS‐DA metabolites (VIP score > 1) with rose red for upregulated and blue for downregulated metabolites in PD‐MC; (i, j) Volcano plot of metabolites in Cohort 2 and Cohort 3, showing expression fold change and significant *p*‐values; upregulated metabolites (log2(FC) > 1) are in red, downregulated (log2(FC) < −1) in blue, with the top 20 metabolites (VIP > 1) highlighted.

Appealed by the intricate interplay between gut microbiota and host co‐metabolism, we further aimed to reveal the spectrum of changes in plasma metabolites through metabolomics. The demographic and clinical characteristics of the subjects involved in the metabolomics assay are presented in Table 1. A total of 246 individuals were enrolled and divided into two independent cohorts, namely Cohorts 2 and 3 (Figure 5a and Table S9). Each plasma sample was analyzed by LC‐MS positive electrospray ionization mode (ESI+) mode to facilitate the ionization and detection of alkaline and acidic metabolites (Figure 5b). After peak detection and alignment analysis, 5780 and 2922 metabolites were identified in cohort 2 and cohort 3, respectively. To simplify the complexity of high‐dimensional data while retaining trends and patterns, we carried out PCA and repeated correlation evaluation in two cohorts, which displayed obvious differences in composition between the PD‐MC vs. PD‐NMC groups (*R* ^2^ = 0.0597, *p* = 0.001, *R* ^2^ = 0.2959, *p* < 0.001, respectively, Figure 5c,f). Moreover, the quality control samples were tightly clustered in the center of the PCA model, indicating the performance stability of the current analysis. Then, the metabolite composition of PD‐MC versus PD‐NMC would still be supported by the distinct clustering patterns in PCoA based on the Bray–Curtis distance matrix [25]. The PD‐MC group presented a distinct clustering pattern of metabolite profile relative to the PD‐NMC (*R* ^2^ = 0.0597, *p* < 0.001, *R* ^2^ = 0.2959, *p* = 0.001, respectively, Figure 5d,g). To maximize the identification of differential features in PD‐MC patients, we further constructed a PLS‐DA model. The plasma metabolome of PD‐MC was separated from PD‐NMC on the score plot in Cohort 3 as well (*R* ^2^ = 0.94, *p* < 0.01, Figure 5h). However, PLS‐DA models between PD‐MC and PD‐NMC in Cohort 2 did not show significant separation (*R* ^2^ = 0.86, *p* = 0.06, Figure 5e). In general, we performed PCA, PCoA, and PLS‐DA models, revealing substantial differentiation in the metabolomic profiles between the two groups. These findings inferred that continuous dopaminergic medications‐related PD‐MC could cause a distinct plasma metabolite profile compared with the PD‐NMC conditions, suggesting metabolomics is probably a promising tool for diagnosing and predicting PD‐MC in the future.

### 2.6 Interpretable Machine Learning on Metabolomics Data

To further understand the differences in metabolites between PD‐MC patients and PD‐NMC controls, we performed a Mann–Whitney *U* test with FDR calibration after adjusting for potential confounders and found that 76 and 210 metabolites were significantly changed in Cohorts 2 and 3, respectively (Figure 5i,j). Meanwhile, the overlap in PD‐MC and PD‐NMC plasma metabolomes of Cohorts 2 and 3 (5740 and 2712, respectively) indicate that PD‐MC may share many common biological features with PD‐NMC subjects (Figure 5i,j). To develop accurate diagnosis models for disease prediction using metabolomics datasets, machine learning methods are widely used [26]. Notably, PLS‐DA is a popular machine learning technique that combines features from PCA and multiple linear regression, which is gaining increasing attention as a useful feature selector and classifier [27]. Of these selected metabolites in Cohort 2, a total of 40 candidate metabolites contributed significantly to the distinction of PD‐MC and PD‐NMC with PLS‐DA VIP > 1 and log_10_FC > 1 (Figure 5k and Table S10). Most of these metabolites were found to be enriched in PD‐MC patients (82.5%), with only a few being depleted (17.5%). Similarly, the plot displayed 23 candidate metabolites that were found to be different between MC and NMC in PD subjects of Cohort 3, among them 14 metabolites were increased and the remaining 9 were decreased in the PD‐MC patients (Figure 5l and Table S11). These metabolites were further annotated based on the database (mzCloud/HMDB/Chemspider library) searching by *m*/*z* value, retention time, and fragmentation mass spectrum [28]. The majority of them belong to the three major metabolism processes, including amino acids, carbohydrates, and fatty acids.

**FIGURE 6:**
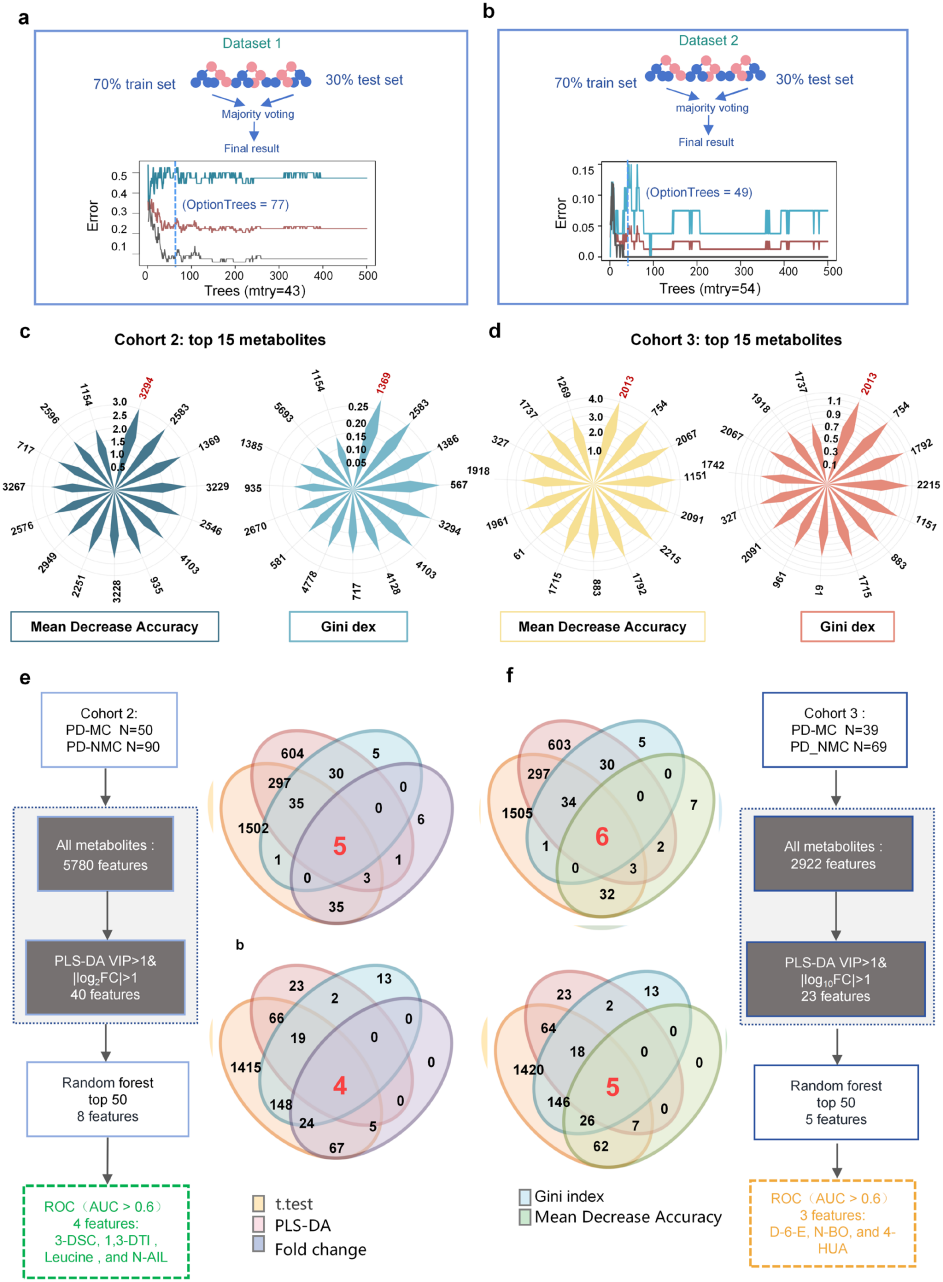
Identification of plasma metabolites linked to motor complications. (a, b) Random forest performance. (c, d) The feature importance map, derived from the random forest regressor, was employed to rank the features based on their significance, as indicated by the mean decrease in accuracy and the mean decrease in the Gini index. Subsequently, the top 15 features were selected and represented using polar graphs for visualization. (e, f) Flowcharts and Venn diagrams for the identification of characteristic core plasma metabolites in Cohort 2 and Cohort 3. Using combined *t*‐test (*p* < 0.05), volcano plots (|logFC| > 1), PLS‐DA (VIP > 1), and the top 50 random forest features, 8 features were identified from 5780 in Cohorts 2 and 5 from 2922 in Cohort 3 ultimately. ROC analysis revealed 4 core metabolites(3‐DSC, 1,3‐DTl, Leucine and N‐AIL) in Cohorts 2 and 3 core metabolites(D‐6‐E, N‐BO and 4‐HUA) in Cohort 3, with AUC > 0.6, related to Figure 7.

Moreover, decision tree‐based statistical methods, for example, Random Forest (RF) have gained prominence. RF is a powerful and versatile supervised machine‐learning algorithm that aims to select important features as potential biomarkers of disease diagnosis or for patient stratification [29]. We performed RF analysis with a 70/30 training and testing split approach, leading to the identification of promising plasma metabolites (Figure 6a,b). By utilizing RF approaches, we ranked a panel of the top 15 metabolites based on the MDA and MDG methods in Cohort 2 and Cohort 3, respectively (Figure 6c,d and Tables S12 and S13), which contributed significantly to the differentiation between PD‐MC and PD‐NMC with high accuracy. We next sought to investigate whether a specific small group of metabolites can serve as an accurate diagnostic tool for PD‐MC. To obtain an optimal variable combination of biomarkers for the diagnosis model, we then integrated the *t*‐test, fold change (FC), PLS‐DA, RF, and AUC > 0.6 machine‐learning methods and identified a total of four metabolites with significantly differential abundance that were commonly present in Cohort 2 (Figure 6e), including 3‐deoxysappanchalcone (3‐DSC), 1,3‐Dimethyluracil (1,3‐DTl), Leucine, and N‐Acetylisoleucine (N‐AIL). Besides, we also screened out three metabolites with significantly altered abundance from Cohort 3 according to the same processes (Figure 6f): Dodec‐6‐enoic acid (D‐6‐E), N‐butyl Oleate (N‐BO), and 4‐hydroxyundecanoic acid (4‐HUA). Additionally, subgroup analyses of PD‐MC were performed in Cohorts 2 and 3. The results of ANOVA indicated a statistically significant difference in the level of 3‐DSC among the three groups in Cohort 2 (*p* = 0.046), with the dyskinesia group exhibiting the highest abundance. Similarly, in Cohort 3, a statistically significant difference was observed in the levels of 7‐methyldecanoic acid (7‐MDCA) across the three groups (*p* = 0.036), with the combined group demonstrating the highest abundance (Table S18).

### 2.7 Plasma Metabolomic Profiles Correctly Classify PD‐MC Samples From PD‐NMC

**FIGURE 7:**
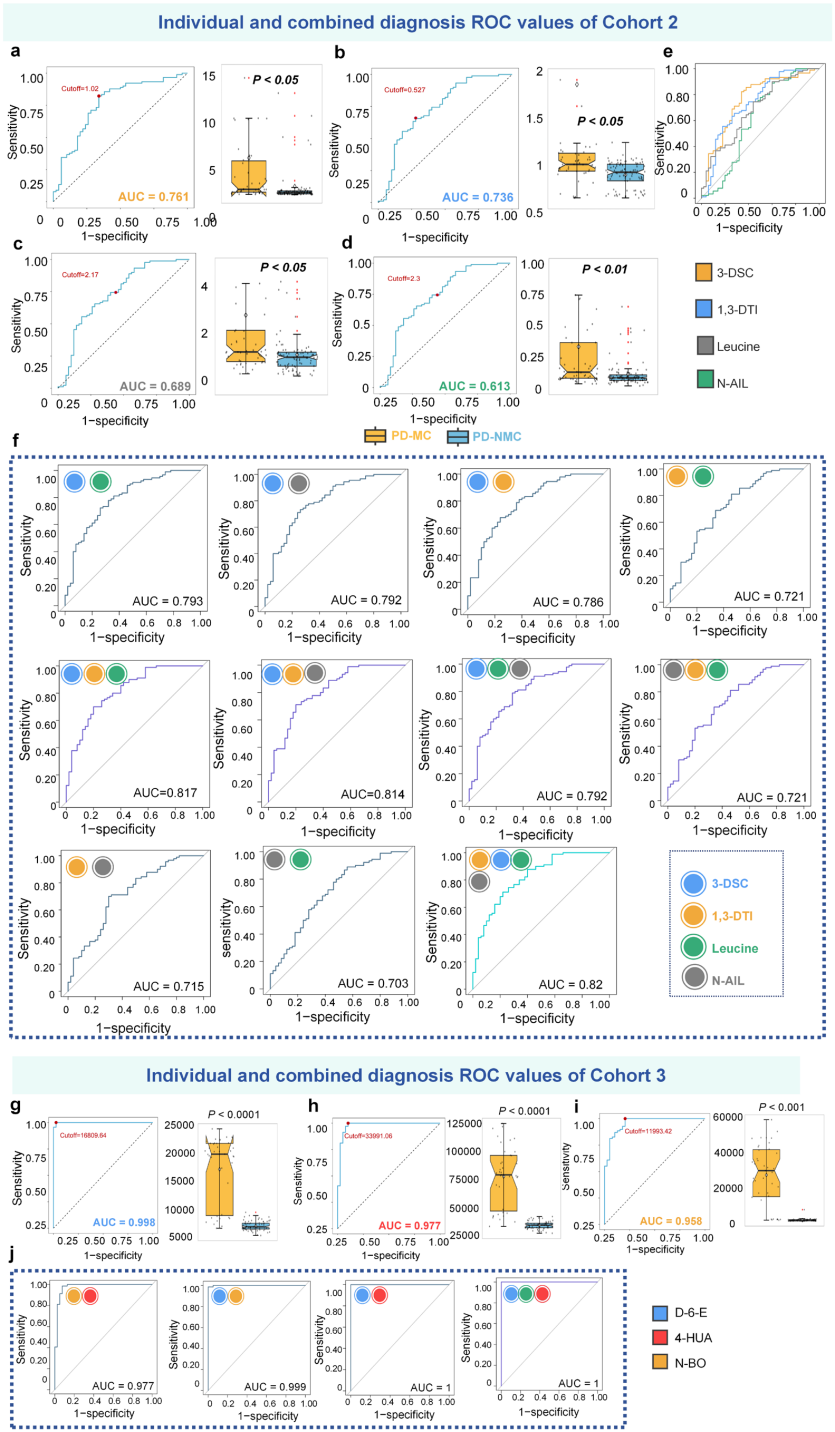
Individual and combined AUC for core plasma metabolites. (a–e) The Individual receiver operating characteristic (ROC) curves of Cohort 2 and abundance expression level box plots for core metabolites are presented. The *p* values and AUC values have been annotated, where (a), (b), (c), (d) represent 3‐DSC (AUC = 0.761), 1,3‐DTI (AUC = 0.736), Leucine (AUC = 0.689) and N‐AIL (AUC = 0.613), respectively. In Cohort 2, combined AUC analysis of the two, three and four core metabolites is illustrated in (f); (g–i) The Individual receiver operating characteristic (ROC) curves of Cohort 3 and abundance expression level box plots for core metabolites are presented. The *p*‐values and AUC values have been annotated, where (g), (h), (i) represent D‐6‐E (AUC = 0.998), 4‐HUA (AUC = 0.977) and N‐BO (AUC = 0.958), respectively. In Cohort 3, combined AUC analysis of the two and three core metabolites is illustrated in (j).

To investigate if the filtrated metabolites could improve the diagnostic accuracy of PD‐MC when compared to the PD‐NMC, we further examined the AUC values of these metabolites. Cohort 2 showed the AUC values of each metabolite were 0.761 (3‐DSC), 0.736 (1,3‐DTl), 0.689 (Leucine), and 0.613 (N‐AIL), respectively (Figure 7a–e). After achieving good diagnostic performance in the above compounds, we further explored whether combining all metabolites could enhance the diagnostic capacity of our model. Notably, the discriminative accuracy of PD‐MC, expressed as the AUC, improved from 0.721–0.793 in the basic model (two identified metabolites) to 0.703–0.817 with the addition of the third metabolite (advanced model), which differed significantly between the PD‐MC and PD‐NMC in the multivariate logistic regression analysis (Figure 7f). Additionally, the discriminant ability was similar when uniting the four metabolites into a biomarker panel with an AUC value of 0.82 in differentiating between PD‐MC and PD‐NMC, as well as 0.868 sensitivity and 0.859 specificity in this set. After that, to our surprise, we found that the Cohort 3 represented the AUC of each metabolite were 0.998 (D‐6‐E, Figure 7g), 0.977 (4‐HUA, Figure 7h), and 0.958 (N‐BO, Figure 7i), respectively. Our results indicate that combining metabolites can enhance the diagnostic performance of PD‐MC vs. PD‐NMC in plasma metabolomic analysis (Figure 7j). These findings suggest that differences in levels of metabolite can differentiate PD‐MC from PD‐NMC. Notably, identifying robust metabolite predictors of PD‐MC occurrence could help to identify new preventive strategies.

### 2.8 Perturbation of Amino Acids and Unsaturated Fatty Acids Pathways Underlie the Distinction Between PD‐MC and PD‐NMC

**FIGURE 8:**
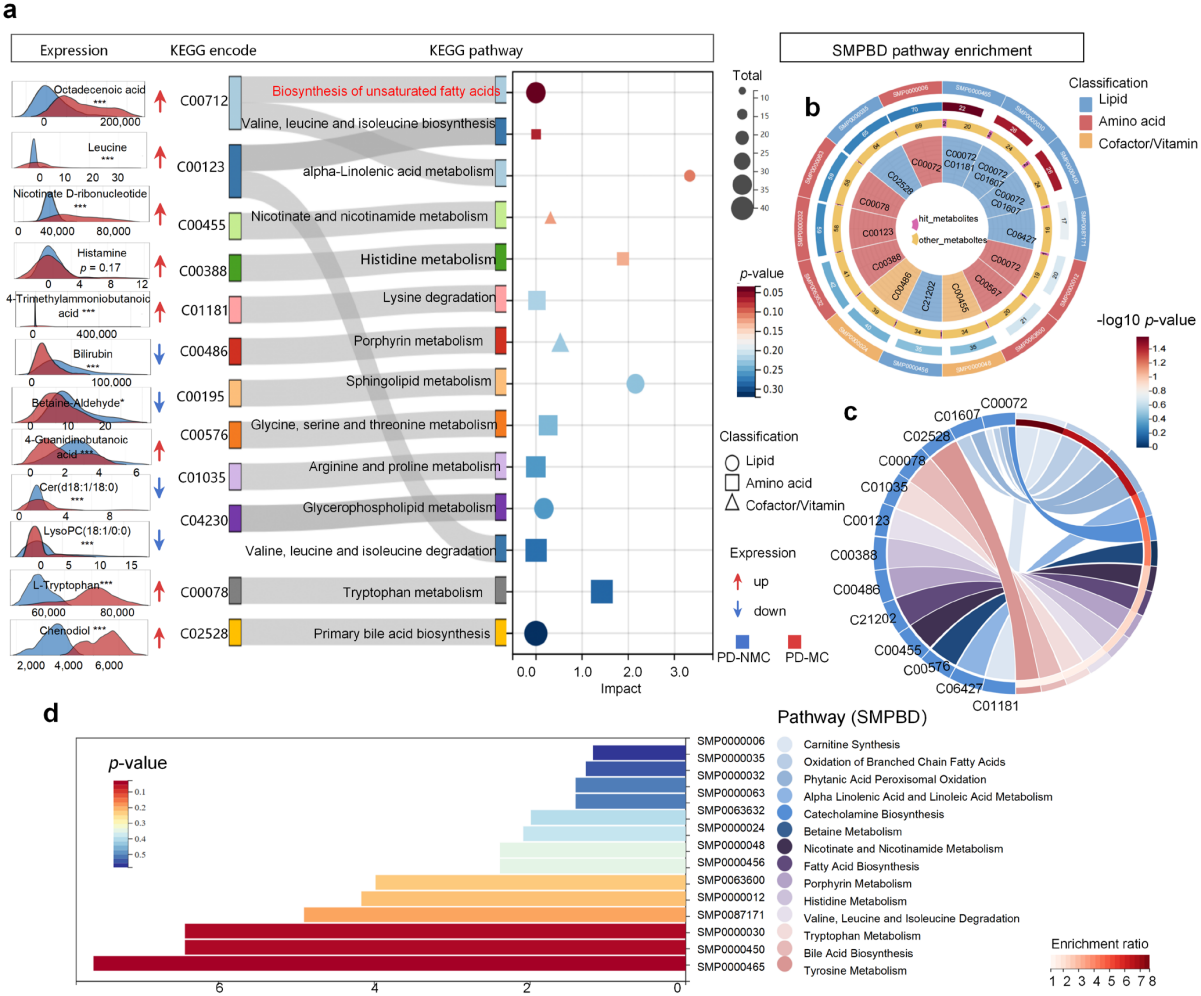
Pathway analysis of key metabolites. (a) The Sankey diagram shows the association between metabolic pathways and metabolites, identified by KEGG codes. The right bubble visualizes pathway classification, biological impact, total metabolite counts, and *p* values. The left peak plot displays key metabolite expression levels in PD‐MC and PD‐NMC groups, highlighting significant differences (*p* < 0.05). *p < 0.05, **p < 0.01, ***p < 0.001. A red arrow indicates metabolite enrichment in the PD‐MC group, while a blue arrow indicates enrichment in the PD‐NMC group. (b) The Small Molecule Pathway Database (SMPDB) are employed for metabolic enrichment analysis, where the outer to inner rings depict key metabolic pathways, −log10(*p* value), and characteristic metabolites, with colors indicating different classes of metabolites. (c, d) The chord diagram illustrates the enrichment rates of specific pathways and the bar graph displays pathway code names.

According to the identified panel of differential metabolites, we sought to define the biological relevance of the dominating metabolic alterations. Pathway enrichment analysis (PEA) was performed to explore changes in metabolic pathways to PD‐MC onset and progression. Then, we excavated KEGG analysis by MetaboAnalyst and found that metabolic dysregulations in the biosynthesis of unsaturated fatty acids (Lipid classification), Valine, leucine, and isoleucine biosynthesis (Amino acid classification), alpha‐linolenic acid (ALA) metabolism (Lipid classification), and Nicotinate/nicotinamide metabolisms (Cofactor/Vitamin classification) et al. [49], might be involved in the etiopathogenesis of PD‐MC (Figure 8a and Table S14). Of note, SMPDB (Small Molecule Pathway Database) pathway analysis reveals these mentioned are crucial for amino acid and fatty acid synthesis essential to human physiology, as the most significant pathways associated with PD‐MC patients. Specifically, the SMPDB is designed to support pathway discovery and elucidation in metabolomics, transcriptomics, proteomics et al. [30]. Then, the enriched pathways and associated metabolites are visualized as the chordal maps (Figure 8b,c and Table S15). The network shows enriched pathways where edges connect pathways that share many plasma metabolites. Analysis of selected plasma metabolites confirmed that tyrosine metabolism, bile acid biosynthesis, tryptophan metabolism, valine, leucine, and isoleucine degradation were obviously in PD‐MC compared with the PD‐NMC by the SMPDB pathway enrichment (Figure 8d). These findings offer additional evidence supporting the hypothesis that PD‐MC and PD‐NMC are characterized by distinct metabolic pathway patterns.

### 2.9 Gut Microbiota Composition Is Linked to Plasma Metabolomic Profiles in PD‐MC

We conducted a Spearman correlation analysis to explore the functional relationship between altered gut microbiota and plasma metabolites in PD‐MC. This analysis was based on a limited sample of patients possessing both 16S rRNA and plasma metabolome data and including a total of 17 PD‐MC (Figures S3a and S5b). Of note, 30 characteristic microorganisms at the genus level and 27 characteristic metabolites uncover a significant correlation (Figure S3b,c and Table S16). Our analysis identified a statistically significant negative correlation between *Agathobacter* and Tyrosy‐Threonine (rho = −0.811, *p* = 0.027). Genera *Ligilactobacillus* exhibited a negative correlation with Tyrosy‐Threonine (rho = −0.867, *p* = 0.012) and a positive correlation with D‐6‐E (rho = 0.705, *p* = 0.02). Moreover, a significant positive correlation was also observed between *Ligilactobacillus* and D‐6‐E (rho = 0.705, *p* = 0.02). Leucine showed a strong negative correlation with *Roseburia* (rho = −0.811, *p* = 0.027) and CAG_56 (rho = −0.867, *p* = 0.012), but a positive relationship with the *Eubacterium* (rho = 0.786, *p* = 0.036). Furthermore, correlation analysis demonstrated a significant negative correlation between N‐BO and *Akkermansia*. In contrast, N‐acetyl was negatively correlated with *Roseburia*, while showing a positive correlation with *Eubacterium*. Finally, a correlation analysis was conducted between the five‐core microbiota and the seven key metabolites (Figure S3d), which revealed a statistically significant positive correlation between *Ligilactobacillus* and D‐6‐E. Subsequently, we conducted repeated trials in all PD patients, observing analogous outcomes with a weak correlation (Figures S4a–c, S5a, and Table S17). These findings further underscore the association between key microbial populations and metabolomic profiles in PD‐MC.

## 3 Discussion

There has been much interest in the identification of features from the gut microbiome or plasma metabolome that may serve as promising biomarkers of PD with the hope that they may be useful in the differentiation of disease status and as prognostic factors in disease outcomes, such as PD‐MC. Our study is the first integrated analysis of the gut microbiome and plasma metabolome in patients with PD‐MC and PD‐NMC. Despite the substantial overlap in the changes of fecal microbiome and plasma metabolome between PD‐MC and PD‐NMC, our analysis was able to establish disease‐related key characteristics that can potentially be used to predict the patients with PD‐MC and their distinct taxonomic subgroups. Furthermore, the features of taxa and metabolites were widely correlated with each other, and integrated analysis showed unique reactions in PD‐MC patients, providing new mechanistic insights into the pathogenesis of PD‐MC conditions. Intriguingly, we propose the utilization of fecal microbiota and plasma metabolites as noninvasive diagnostic tools, which may facilitate the early detection and prediction of PD‐MC, thereby promoting timely interventions and mitigating the risk of motor complications. In addition, considering the emerging evidence in animal models that the microbiota and metabolites play an important role in neurodegeneration disease or PD [31, 32], such biomarkers might offer new ideas into disease etiopathogenesis, perhaps leading to novel therapeutic methods.

Alterations in gut microbial community composition and plasma metabolites were common features in PD patients compared to controls. Many consistently altered differential taxa and metabolites have been identified in PD subjects [7, 33], which may indicate a general mechanism of PD. However, whether fecal microbiome dysbiosis and plasma metabolomic profiles are involved in PD progression, particularly in the development of PD‐MC, is still undetermined. There is almost no study on this issue, and the current project just fills this gap. Herein, rather than focusing on PD, we performed a multiomics analysis of later‐stage disease (i.e., PD‐MC) to identify microbiome and metabolome‐related features that might be of value as biomarkers to monitor motor complications for PD patients.

In this study, despite the partial overlap in the diversity of the fecal microbiome between PD‐MC and PD‐NMC patients, the spatial distribution was driven by their unique taxa signatures. For instance, an increase in fecal *Lactobacillus*, *Limosilactobacillus*, *Bifidobacterium*, and *Ligilactobacillus* genera along with depleted *Agathobacter* was detected in PD‐MC groups, coupled with the induction of the 3‐DSC, 1,3‐DTl, Leucine, N‐Acetylisoleucine, D‐6‐E, N‐BO, and 4‐HUA plasma metabolites in PD‐MC subjects. Building upon these specific findings, we can propose potential mechanistic links to the pathogenesis of PD‐MC. The observed depletion of Agathobacter, a recognized producer of butyrate, indicates a reduction in beneficial short‐chain fatty acids (SCFAs), which are essential for maintaining gut barrier integrity and exerting anti‐inflammatory effects. Simultaneously, the elevated levels of branched‐chain amino acids (e.g., Leucine, N‐Acetylisoleucine) may modulate systemic immune responses. We hypothesize that this convergence of events—a decline in protective SCFAs coupled with an increase in immunomodulatory amino acids—may create a systemic environment that exacerbates neuroinflammation and contributes to the severity of motor complications via the gut‐brain axis [14]. These results are in line with the dominance of the microbiome and metabolome in associating with deviations of PD‐MC‐enriched, which further highlights the deteriorative role of the gut microbiome in PD‐MC. Overall, through the integration of multidimensional omics data, we have constructed a significant enhancement in the performance of our machine learning models for diagnosing PD‐MC, or even treatment.

Most studies to date analyzed PD‐MC patients in bulk, searching for population‐level risk factors (e.g., gender, age, BMI index, medications, or dosage), instead of focusing on the biological variability at an individual level. In the present study, we analyzed the individual‐level fecal microbiota deviations of PD‐MC patients and found that these variations are person‐specific, concerning the genetic and environmental factors underlying their levels. The basic characteristics of gut dysbiosis observed in PD‐MC included increased alpha diversity, converted microbial community, and altered abundances of individual taxa. Notably, it has been reported that an increase in fecal *Lactobacillus*, *Limosilactobacillus*, *Bifidobacterium*, and *Ligilactobacillus* genera along with depleted *Agathobacter* genera in PD‐MC group. Among them, *Lactobacillus* constitutes a significant component of the human and animal microbiota, and supplementation with *Lactobacillus* inhibits the permeability of gut barriers in PD Model [34]. In this study, we found high *lactobacillus* in the PD‐MC group may be partly because PD‐MC people have a longer disease duration and are more likely to take probiotics for a longer time.

By comparing the serum profiles of PD‐MC patients with PD‐NMC matched controls, it is noteworthy that we found a unique metabolomics signature of PD‐MC, with hundreds of metabolites significantly perturbed when compared with PD‐NMC. Finally, metabolomic analysis revealed that levels of 3‐DSC, 1,3‐DTl, Leucine, N‐Acetylisoleucine, D‐6‐E, N‐BO, and 4‐HUA were upregulated in PD‐MC patients. These metabolites could be partially explained by differences in the fecal microbiota and their corresponding enzymes involved in amino acid metabolism and synthesis. A growing body of evidence shows that amino acid biosynthesis is upregulated in PD patients, potentially serving as a useful marker for PD [35]. In this study, we found that the distinction between PD‐MC and PD‐NMC is underpinned by significant alterations in KEGG pathways related to amino acids and unsaturated fatty acids. This is consistent with reports that serum amino acid profiles may serve as biomarkers of PD progression [36], and that unsaturated fatty acids—key neuronal membrane components with neuroprotective, antioxidant, and anti‐inflammatory properties [37]—represent a potential therapeutic strategy for PD.

Building on these metabolic disturbances, our findings support an integrated pathogenic model for PD‐MC. Recent genetic evidence has demonstrated bidirectional causal links between plasma metabolites and Parkinson's disease [38]. In PD‐MC, we propose that metabolic dysregulation converges specifically on impairment of the kynurenine pathway—promoting oxidative stress and excitotoxicity—combined with perturbations in unsaturated fatty acids that undermine neuronal integrity and anti‐inflammatory signaling. We hypothesize that this synergistic imbalance generates a proinflammatory milieu that drives PD‐MC progression, underscoring the need to investigate master regulators such as the NRF2‐autophagy axis [39].

In conclusion, our comprehensive analysis enables us to propose a preliminary model of PD‐MC pathogenesis, primarily driven by disruptions in the gut–brain axis. This model suggests that alterations in the gut microbiome, characterized by a reduction in SCFA producers such as Agathobacter and an increase in specific genera, lead to a significant shift in the metabolic environment. This metabolic shift, marked by modified bile acid profiles, increased amino acid levels, and disrupted fatty acid compositions, is hypothesized to induce systemic inflammation and impair the integrity of the blood–brain barrier. These peripheral alterations are posited to affect the central nervous system by potentially exacerbating neuroinflammation and oxidative stress, thereby contributing to the nigrostriatal degeneration associated with motor complications. Although this model necessitates direct experimental validation, it offers a mechanistic framework to inform and guide future research endeavors. Of course, the study suffers from some limitations that must be acknowledged. First, nontargeted metabolomics analyses of plasma samples demonstrated variability in the total number of metabolites identified across two independent tests. This variability was attributed to factors such as sample pretreatment, technical fluctuations, batch effects, database matching, and the stringency of identification criteria. Despite these differences, the internal data quality for each independent test underwent rigorous evaluation to ensure the reliability of the results. To enhance the robustness of the research, two separate experiments (Cohort 2 and Cohort 3) were conducted, followed by comprehensive statistical analyses. Furthermore, the identification of differential metabolites was based on high‐confidence MS/MS spectral matching (Level 2 according to the Metabolomics Standards Initiative). While this represents the current best practice for untargeted discovery, definitive confirmation with authentic chemical standards is required in future targeted studies. Second, the comprehensive multi‐omics analysis of the intestinal microbiome and plasma metabolome was constrained to a cohort of 17 patients with PD‐MC, as data pertaining to both their microbiota and metabolome were accessible. This limitation may compromise the robustness of the observed associations among the metabolites derived from the microbiota. Additionally, it is crucial to acknowledge that this study employs a single‐center observational cohort design, which inherently limits the ability to infer causal relationships from the observed associations. To substantiate these findings and explore the underlying mechanisms in greater depth, further research should be conducted using external independent cohorts and complemented by animal experiments for functional verification. Finally, it is essential to note that the collection of samples from PD may be influenced by various factors, including the use of probiotics and prebiotics, traditional Chinese medicine, and other complicating variables. The current analysis does not evaluate the potential effects of these factors.

## 4 Methods

### 4.1 Ethics Approval and Consent to Participate

Participants for this study were enlisted from both the outpatient and inpatient units of the Neurology Department at the First Affiliated Hospital of Wenzhou Medical University, during the period from March 2018 to August 2024. This study received approval from the Institutional Ethics Committee of the First Affiliated Hospital of Wenzhou Medical University. Prior to participation, all involved individuals provided written informed consent.

### 4.2 Experimental Design and Subject Recruitment

Kindly refer to Figure 1 which delineates the flowchart of the study design for the current research. In this study, a cohort of 108 participants diagnosed with Parkinson's disease was recruited for gut microbiome analysis, while an additional 246 participants with Parkinson's disease were enrolled for nontargeted metabolomics analysis. This study included PD patients aged 40‐80, diagnosed according to Movement Disorder Society (MDS) clinical diagnostic criteria [40, 41]. The exclusion criteria encompassed Secondary Parkinson's disease, other neurodegenerative disorders (e.g., Alzheimer's disease), and additional significant conditions that impact motor function, cognitive abilities, mood, mental status, or capacity to perform daily activities. Furthermore, individuals with severe speech or hearing impairments were also excluded. All participants underwent a comprehensive clinical assessment at the time of initial inclusion, which encompassed the collection of demographic information such as age, gender, body mass index (BMI), smoking status, alcohol consumption, and comorbidities. Furthermore, clinical scales and medication usage were documented and are detailed in Table 1. Patients with Parkinson's disease (PD) were divided into two groups based on motor symptom fluctuations and dyskinesia: those with motor complications (PD‐MC) and those without (PD‐NMC). For each participant diagnosed with PD, both fecal and blood samples were collected by a medical professional during their initial clinical consultation. This study encompasses three cohorts of Parkinson's disease (PD), namely Cohort 1 (PD‐MC: *n* = 42, PD‐NMC: *n* = 66), Cohort 2 (PD‐MC: *n* = 50, PD‐NMC: *n* = 90), and Cohort 3 (PD‐MC: *n* = 37, PD‐NMC: *n* = 69). Stool samples from Cohort 1 underwent 16S rRNA gene sequencing, while serum samples from Cohorts 2 and 3 were analyzed with untargeted metabolomics. A total of 37 PD patients across the three cohorts underwent simultaneous plasma untargeted metabolomics analysis (PD‐MC: *n* = 17, PD‐NMC: *n* = 20), and we performed a multiomics correlation analysis based on these data. The characteristics of the subjects are delineated in Table 1.

### 4.3 Questionnaire and Clinical Assessment

All participants diagnosed with PD were subjected to a thorough clinical scale evaluation upon their enrollment in the study. We evaluated motor symptom severity and progression stage of PD with a modified Unified Parkinson's Disease Rating Scale (UPDRS III) and Hoehn‐Yahr staging (H‐Y) [42]. PD‐MC was thoroughly characterized by dyskinesia as indicated in the UPDRS‐IV, fluctuations in motor symptoms, and a reported decrease in drug efficacy by patients. Additionally, participants with PD‐MC were categorized into three distinct groups for further subgroup analysis: the dyskinesia group, the symptom fluctuation group, and the combined group, which exhibited both conditions concurrently. Nonmotor symptoms (NMS) can be categorized into sensory, sleep, autonomic, cognitive, and psychiatric domains [43]. The evaluation of NMS is conducted through various assessment instruments, such as the Mini‐Mental State Examination (MMSE), the Hamilton Depression Rating Scale (HAMD), the Hamilton Anxiety Inventory (HAMA), the Hong Kong Rapid Eye Movement Sleep Behavior Disorder Questionnaire (RBDQ‐HK), and the Revised Activities of Daily Living Scale (ADL). The assessment additionally encompassed the Levodopa Equivalent Daily Dose (LEDD) and complications associated with Parkinson's Disease, such as falls, constipation, dyskinesia, and on‐off phenomena.

### 4.4 Collection of Stool Samples and 16S rRNA Microbiota Sequencing

During the registration procedure, more than 1 g of fresh fecal matter was collected using a sterile, disposable fecal sampling tube, and subsequently preserved at a temperature of −80°C within a 30‐min interval for subsequent analysis. Specific stool sampling tubes, filled with 5 mL of stool sample nucleic acid preservation solution, were used to gather in excess of 1 mL of fresh stool solution, which was subsequently stored at a temperature of −80°C until microbiota sequencing. To assess the diversity and composition of the gut microbiota, fecal microbiota analysis was conducted via PCR amplification. Total genomic DNA was extracted from fecal samples employing the cetyltrimethylammonium bromide (CTAB) method. All PCR reactions were prepared with 15 μL of Phusion ➅ High‐Fidelity PCR Master Mix (New England Biolabs), 0.2 μM of specific primers (341F and 806R), and 10 ng of genomic DNA template. The thermal cycling protocol entailed an initial denaturation phase at 98°C for 1 min, succeeded by 30 cycles comprising denaturation at 98°C for 10 s, annealing at 50°C for 30 s, and extension at 72°C for 30 s. A final extension step was conducted at 72°C for 5 min. This protocol was employed for the amplification of the 16S ribosomal RNA (16SrRNA) gene, specifically targeting the highly variable V3‐V4 regions. The mixed PCR products obtained were subsequently purified employing the Qiagen Gel Extraction Kit (Qiagen, Germany). The libraries were then systematically sequenced, indexed, and validated using Qubit fluorometry, real‐time PCR, and bioanalyzer assessments. Following these procedures, the libraries were pooled and sequenced on an Illumina platform, with pooling decisions guided by library concentrations and specific data requirements [43].

### 4.5 Analysis of the Microbial Composition

Initially, data quality control was conducted, with comprehensive methodologies outlined in the attachment titled “QC_methods for details” (refer to Supporting Information for additional information). Following this, UPARS software (UPARS v7.0.1001, http://drive5.com/uparse/) was employed to cluster sequences exhibiting ≥ 97% similarity into the same Operational Taxonomic Units (OTUs). Each representative OTU sequence was subsequently screened and taxonomically annotated utilizing the Silva database (http://www.arb‐silva.de/) in accordance with the mothur algorithm. Following this, alpha and beta diversity analyses were performed on the normalized data. Alpha diversity within the sample was evaluated using three indices in QIIME1: Chao1, Shannon, and Simpson, to assess community diversity, richness, and evenness. Beta diversity was analyzed through principal component analysis (PCA) and principal coordinate analysis (PCoA). PCA was utilized to reduce the dimensionality of the original variables, employing the ade4 and ggplot2 packages in R software (version 4.3.2) before conducting cluster analysis. PCoA was applied to simplify and visualize the complex, multidimensional data. This involved creating a distance matrix from the samples, which was then transformed into orthogonal axes to emphasize the factors contributing to maximum variation. The analyses were conducted using the ade4 and ggplot2 packages in R software (version 4.3.2).

### 4.6 Identification of PD‐MC‐Related Microbial Species

(1) The UPARSE software (version 7.0.1001, http://www.drive5.com/uparse/) was employed to cluster all effective tags from all samples, resulting in sequences being grouped into Operational Taxonomic Units (OTUs) with 97% similarity [44]. Subsequently, species annotation and statistical analyses across various taxonomic levels were conducted by comparing the sequences with the Silva138 database (http://www.arb‐silva.de/) [45]. (2) The alpha diversity indices, including Chao1, Shannon, Simpson, and ACE, were computed using QIIME software (Version 1.9.1) to assess the abundance and diversity of the microbiota. Beta diversity was evaluated utilizing PCA, PCoA, and nonmetric multidimensional scaling (NMDS). This analysis was used to reduce the dimensionality of the original variable using the ade4 package with R software and the ggplot2 package (version 4.2.1). (3) Metagenomic‐Seq assays were conducted to compare microbial species abundance at different taxonomic levels between groups, applying thresholds of fold change (FC) > 2 or FC < 0.5 and a significance level of *p* < 0.05 to identify species with significant differences. The Metastats software (http://metastats.cbcb.umd.edu/) was employed to evaluate differences in species abundance between groups, yielding *p* values. These *p* values were subsequently adjusted using the false discovery rate (FDR) method. (4) LEfSe is an analytical methodology developed for the identification and interpretation of high‐dimensional biomarkers, which emphasizes the importance of both statistical significance and biological relevance, facilitating the identification of biomarkers that demonstrate substantial differences between groups across multiple taxonomic levels. The LEfSe tool, available publicly, is used for this analysis, considering taxa with a *p* value < 0.05 and the log LDA score ≥ 2. (5) The random forest algorithm, a well‐established machine learning model derived from the classification tree algorithm, was employed to evaluate trait importance. Microorganisms were selected based on the highest scores of Mean Decrease in Gini and Mean Decrease in Accuracy, as determined through Random Forest feature selection. (6) Receiver Operating Characteristic (ROC) analysis was conducted using the pROC package in R, following a random forest screening of characteristic species [46]. An area under the curve (AUC) value exceeding 0.6 was utilized as the evaluation criterion.

### 4.7 Collection of Plasma and Non‐Targeted UHPLC–MS/MS Analysis

Venous blood samples were collected into tubes containing EDTA and subsequently centrifuged at 3000 rpm for 10 min to facilitate plasma separation. The isolated plasma was aliquoted into 250 μL portions and stored at −80°C until further analysis. For metabolomic extraction, 150 μL of plasma was mixed with 400 μL of a pre‐chilled methanol/water/dichloromethane solution (1:1:2, v/v/v). This mixture was vortexed for 60 s and then centrifuged at 15,000 rpm for 15 min at 4°C to achieve phase separation. The upper aqueous‐methanol phase, containing polar metabolites, and the lower organic phase, containing lipophilic compounds, were carefully collected into separate tubes, ensuring the intermediate protein precipitate was not disturbed. Both fractions were evaporated to dryness under a gentle stream of nitrogen gas. The polar extract was reconstituted in 100 μL of acetonitrile/water (1:1, v/v), while the lipophilic extract was reconstituted in 125 μL of chloroform/methanol (1:1, v/v). These reconstituted solutions were centrifuged at 15,000 rpm for 15 min at 4°C, and the resulting supernatants were analyzed using UHPLC–MS/MS. A pooled quality control (QC) sample, created by combining equal volumes of all individual samples, was systematically analyzed at regular intervals throughout the analytical sequence to ensure instrument stability and data reproducibility. Chromatographic separation was conducted using a SHIMADZU CBM‐30A Lite LC system in conjunction with an API 6600 Q‐TRAP mass spectrometer. The mass spectrometer operated in electrospray ionization positive (ESI+) mode, scanning a mass range from m/z 80 to 1200. For the analysis of polar metabolites, separation was performed on a Waters Acquity amide‐based hydrophilic interaction liquid chromatography (HILIC) column (2.1 × 100 mm, 1.7 μm). The mobile phase comprised (A) water with 5 mM ammonium acetate and 0.1% formic acid, and (B) acetonitrile, delivered at a flow rate of 0.3 mL·min^−1^. The gradient elution program was as follows: 0–0.5 min, 98% B; 0.5–13 min, 98% to 40% B; 13–13.1 min, 40% to 98% B; 13.1–18 min, 98% B. The column temperature was maintained at 35°C, and the injection volume was set to 2 μL. For the analysis of lipids, chromatographic separation was performed using a Phenomenex Kinetex C18 column (dimensions: 2.1 × 100 mm, particle size: 2.6 μm). The mobile phase consisted of two components: (A) a mixture of water, methanol, and acetonitrile in a volumetric ratio of 3:1:1, supplemented with 5 mM ammonium acetate, and (B) isopropanol. The flow rate was maintained at 0.3 mL·min^−1^. The gradient elution program was as follows: from 0 to 0.5 min, 25% B; from 0.5 to 1.5 min, a linear increase from 25% to 40% B; from 1.5 to 3 min, a further increase from 40% to 60% B; from 3 to 13 min, a gradient from 60% to 98% B; from 13 to 13.1 min, a rapid decrease from 98% to 25% B; and from 13.1 to 18 min, 25% B was maintained. The column temperature was controlled at 40°C, and the injection volume was set at 1 μL. For both analytical procedures, the collision energy applied for fragmentation was 40 V. The confidence level for the identification of differential metabolites was assigned according to the guidelines of the Metabolomics Standards Initiative [47]. Identifications based on the matching of high‐resolution MS/MS fragmentation spectra to reference spectra in the mzCloud and HMDB databases were assigned as Level 2 (putative annotation).

### 4.8 Identification of PD‐MC‐Related Plasma Differential Metabolites

The initial raw data were collected and subsequently processed into mzXML format utilizing the MassHunter workstation software. This is followed by a comprehensive data preprocessing phase conducted with the XC‐MS software (XC‐MS Plus, California, United States). The preprocessing phase includes nonlinear retention time alignment, peak identification, filtering, alignment, matching, and subsequent identification [48]. The primary parameter settings are delineated as follows: peak width = 50, Bw = 0.1, Mzwid = 0.1, and Mbundiac = 80%. Subsequently, the 3D raw dataset, encompassing *m*/*z* values, retention time (RT), and peak intensity, is exported to Microsoft Excel 2020 (Microsoft, Redmond, WA). Metabolite identification was conducted by cross‐referencing precise m/z values and MS/MS spectral data with the HMDB 4.0 database. Subsequent annotation was carried out utilizing the online platforms MetDNA2 (available at http://metdna.zhulab.cn/) and One‐MAP (accessible via http://www.5omics.com/). Prior to performing univariate and multivariate data analyses, the LC‐MS raw data are normalized using Log10 transformation and Pareto scaling. We identified differential metabolites based on the following criteria: (1) Univariate statistical analysis was employed to assess the statistical significance of metabolites between the two groups using the *t*‐test, with a significance threshold set at *p* < 0.05. Additionally, the fold change in metabolite levels (FC values) between the two groups was calculated, with metabolites classified as differential if FC ≥ 2 or FC ≤ 0.5. The volcano plot was generated utilizing the R package ggplot2, which facilitated the integration of log2(Fold Change) and log10(*p* value) values to identify metabolites of interest. (2) Multidimensional statistical analysis was employed to examine the differences in metabolite distribution between groups using PCA and PCoA, with a significance threshold set at *p* < 0.05. Partial Least Squares Discriminant Analysis (PLS‐DA) was employed to elucidate the relationship between metabolite expression and group classification. The model's performance was assessed by the goodness‐of‐fit (R^2^Y, denoted as R2 in figures) and the predictive ability (Q^2^, denoted as Q2 in figures). Metabolites with Variable Importance in Projection (VIP) scores exceeding 1 were identified as significant. This analysis was conducted using the R package “mixOmics.” (3) In the implementation of a Random Forest (RF) model, the dataset was divided into training and validation sets in a 7:3 ratio. For the analysis incorporating Cohort 1 and Cohort 2 (PD‐MC, *n* = 7), the Random Forest model was constructed utilizing the randomForest package in R, with parameters set to mtry = 43 and ntree = 77. Conversely, for the analysis integrating Cohort 1 and Cohort 3 (PD‐MC, *n* = 10), the model was developed with mtry = 54 and ntree = 49. The training set is utilized for model optimization, while the out‐of‐bag (OOB) error serves as a metric for assessing the model's performance. The random Forest package in R is used to determine the optimal number of decision trees that produce the lowest error rate to achieve the most favorable OOB error value. The feature importance score, represented by the average reduced precision and indicating features contributing to the model's accuracy, was calculated using the random forest algorithm. The top 50 important features were subsequently identified. The performance of the random forest model was evaluated through ROC analysis. The AUC and the corresponding 95% confidence interval (CI) were determined using the R package “pROC” with 10,000 bootstrap replicates. Metabolites with an AUC greater than 0.6 were considered characteristic.

### 4.9 Kyoto Encyclopedia of Genes and Genomes (KEGG) Enrichment Analysis

MetaboAnalyst 6.0 (https://new.metaboanalyst.ca/) was employed to predict the enrichment pathways of differential metabolites. Following the matching and annotation of metabolite identifiers within the KEGG, PubChem, and HMDB databases, enrichment analysis and pathway analysis were conducted utilizing the KEGG and SMPDB pathway databases.

### 4.10 Integrated Microbiome‐Metabolite Correlation Analysis

Spearman correlation analyses were conducted using the cor.test function in R to evaluate the associations between differential microbial taxa and metabolites. These analyses were performed separately for the entire PD cohort and the PD‐MC subcohort. For the comprehensive PD analysis, we included patients overlapping between Cohort 1 and Cohort 2 (*N* = 16) as well as between Cohort 1 and Cohort 3 (*N* = 21). For the PD‐MC analysis, we incorporated overlapping PD‐MC patients between Cohort 1 and Cohort 2 (*N* = 7) and between Cohort 1 and Cohort 3 (*N* = 10). In each scenario, the union of all features with calculable correlations from the two respective dataset pairs was assembled for visualization using the Corrplot package. A *p* value of less than 0.05 was considered statistically significant.

### 4.11 Statistics Analysis

Statistical analyses were conducted utilizing R (version 4.3.2) and SPSS Statistics (version 27). For data exhibiting a normal distribution, comparisons between groups were made using independent Student's *t*‐tests or Pearson chi‐squared tests, as appropriate. In cases where data were not normally distributed, the Mann‐Whitney *U* test was employed for nonparametric analysis. The predominant approach for addressing multiple comparisons is the application of the FDR correction. In this study, *p* values were adjusted to *q* values utilizing the Benjamini‐Hochberg FDR method, defined as *q* value(*i*) = *p*(*i*) × length(*p*)/rank(*p*). Significant interspecies differences were identified through species analysis based on either *p* values or *q* values. A multivariate diagnostic model, predicated on characteristic microorganisms and metabolites, was developed. The diagnostic efficacy of the model was evaluated by calculating the AUC. Further, Subgroup analysis was executed using a one‐way analysis of variance (ANOVA), and intergroup comparisons were facilitated through the Least Significant Difference (LSD) method. The results of the generalized Linear Model (GLM) were used to adjust for basic demographic information (age, gender, BMI, education level, disease duration), nonmotor symptoms (MMSE, HAMA, HAMD, RBD, constipation), and LEED for differential analysis of the core microbiota. The significance selection of Omnibus was *p* < 0.01.

## 5 Conclusions

This study presents the first comprehensive multiomics profiling of the fecal microbiome and plasma metabolome in PD‐MC patients, revealing distinct patterns of change. Overall, these results unravel new paradigms and therapeutic avenues for PD‐MC, which may constitute the basis for future mechanistic excavation, preclinical, and human interventional studies.

## Author Contributions

H.G. and C.X. designed and coordinated the project. X.X. and J.H. prepared the samples and analyzed the data. Y.Z., T.J., W.Q., and Q.D. recruited the participants, J.X. and J.Q. made substantial contributions to conception and replenished the required data. S.Q. and C.X. were involved in drafting the manuscript.

## Funding

This work was supported by Projects of the National Science Foundation of China, No. 82001363, No. 81600977, No. 82271469, the Projects of the Natural Science Foundation of Zhejiang Province, LQ23H090007, Y19H090059, LZ23H090001, Leading Innovative and Entrepreneur Team Introduction Program of Zhejiang, No. 2023R01002, the “Pioneer” and “Leading Goose” R&D Program of Zhejiang Province, 2025C02111, and The Projects of the Wenzhou City Committee of Science and Technology, No. Y20220164, 2024Y0194.

## Ethics Statement

The study was approved by the institutional Ethics Board Committee of the Wenzhou Medical University First Affiliated Hospital (KY2021‐153). All participants provided written informed consent before participating in this study.

## Consent

The authors have nothing to report.

## Conflicts of Interest

The authors declare no conflicts of interest.

## Supporting information


**Figure S1:** Differential microbiota correlation in PD‐NMC groups. Spearman correlation analysis of the characteristic microbial abundances, as depicted in Figure 3e, revealed a significant association among the microbiota in PD‐NMC. The phylus levels are represented by nodes of different colors, while the edges indicated positive (red) or negative (blue) correlations. The thickness of the edges corresponds to the magnitude of the correlation coefficients; All correlation *p* values are below 0.05. Detailed correlation coefficients and *p* values are provided in the right heat map.
**Figure S2:** Combined AUC of Cohort 1 potential microbiota. In Cohort 1, combined AUC analysis of the two, three and four core metabolites is illustrated in Figure S1, related to Figure 4.
**Figure S3:** Integrated analysis of multiomics in PD‐MC. (a) Venn diagrams showed PD‐MC participants overlap in Cohorts 1, 2 and 3, revealed shared PD‐MC patients in Cohorts 1 and 2 (*N* = 7) and in Cohorts 1 and 3 (*N* = 10). (b) The network analysis demonstrated statistically significant and suggestive associations (*p* < 0.05, Spearman analysis) among differentially abundant microbiota taxa (illustrated in red) and metabolites (illustrated in blue). Edges connecting the nodes represent positive (red) or negative (blue) correlations. Node numbers corresponding to microorganisms and metabolites are provided in the accompanying legend, with core elements emphasized in red and blue. (c) Correlation chord diagram of microorganisms and metabolites in PD‐MC. (d) Core Microbiota–Metabolites correlation heat map; *p* < 0.05*, *p* < 0.01**, *p* < 0.001***.
**Figure S4:** Integrated analysis of multiomics in PD patients. (a) Venn diagrams showed PD participants overlap in Cohorts 1, 2 and 3, revealed shared PD patients in Cohorts 1 and 2 (*N* = 16) and in Cohorts 1 and 3 (*N* = 21). (b) The network analysis demonstrated statistically significant and suggestive associations (*p* < 0.05, Spearman analysis) among differentially abundant taxa (illustrated in red) and metabolites (illustrated in blue). Edges connecting the nodes represent positive (red) or negative (blue) correlations. Node numbers corresponding to microorganisms and metabolites are provided in the accompanying legend, with core elements emphasized in red and blue. (c) Core correlations between microbiota and metabolites are illustrated using network and heat maps; *p* < 0.05*, *p* < 0.01**, *p* < 0.001***.
**Figure S5:** Workflow for integrated microbiome–metabolite analysis. (a) Analysis workflow for the overall PD cohort. (b) Analysis workflow for the PD‐MC subcohort. Both workflows illustrate the process of performing independent Spearman correlation analyses on overlapping patients from different cohort pairs and merging all calculable features for visualization in network and heatmap plots (see Figures S3 and S4). The specific sample sizes and the number of features calculated vary between the two cohorts.

## Data Availability

The datasets used and/or analyzed during the current study are available from the corresponding author on reasonable request.
