# Supplementary material for: Exploring the Fecal Microbiome Dysbiosis and Its Plasma Metabolome Determinants in Advanced Parkinson's Disease With Motor Complications
Source: CNS Neurosci Ther. 2026 Jan 23;32(1):e70750. doi: 10.1002/cns.70750 (PMC12828341; doi:10.1002/cns.70750)
Supplement: Supplementary file 1 — Figure S1: Differential microbiota correlation in PD‐NMC groups. Spearman correlation analysis of the characteristic microbial abundances, as depicted in Figure 3e, revealed a significant association among the microbiota in PD‐NMC. The phylus levels are represented by nodes of different colors, while the edges indicated positive (red) or negative (blue) correlations. The thickness of the edges corresponds to the magnitude of the correlation coefficients; All correlation p values are below 0.05. Detailed correlation coefficients and p values are provided in the right heat map. Figure S2: Combined AUC of Cohort 1 potential microbiota. In Cohort 1, combined AUC analysis of the two, three and four core metabolites is illustrated in Figure S1, related to Figure 4. Figure S3: Integrated analysis of multiomics in PD‐MC. (a) Venn diagrams showed PD‐MC participants overlap in Cohorts 1, 2 and 3, revealed shared PD‐MC patients in Cohorts 1 and 2 (N = 7) and in Cohorts 1 and 3 (N = 10). (b) The network analysis demonstrated statistically significant and suggestive associations (p < 0.05, Spearman analysis) among differentially abundant microbiota taxa (illustrated in red) and metabolites (illustrated in blue). Edges connecting the nodes represent positive (red) or negative (blue) correlations. Node numbers corresponding to microorganisms and metabolites are provided in the accompanying legend, with core elements emphasized in red and blue. (c) Correlation chord diagram of microorganisms and metabolites in PD‐MC. (d) Core Microbiota–Metabolites correlation heat map; p < 0.05*, p < 0.01**, p < 0.001***. Figure S4: Integrated analysis of multiomics in PD patients. (a) Venn diagrams showed PD participants overlap in Cohorts 1, 2 and 3, revealed shared PD patients in Cohorts 1 and 2 (N = 16) and in Cohorts 1 and 3 (N = 21). (b) The network analysis demonstrated statistically significant and suggestive associations (p < 0.05, Spearman analysis) among differentially abundant taxa (il [file CNS-32-e70750-s001.zip › Supple_documentary/Supplementary documents_table.docx]

**Supplementary documents**

**(tables)**

**Supple Table 1: Relative abundance values of the top 10 species among different taxonomic groups (related to Fig 2e ，f)**

| **Relative abundance of phylus taxonomic** | | | **Relative abundance of class taxonomic** | | | **Relative abundance of order taxonomic** | | | **Relative abundance of family taxonomic** | | | **Relative abundance of genus taxonomic** | | | **Relative abundance of species taxonomic** | | |
| --- | --- | --- | --- | --- | --- | --- | --- | --- | --- | --- | --- | --- | --- | --- | --- | --- | --- |
| **Phylus**  **taxonomic** | **PD-MC** | **PD-**  **NMC** | **Class taxonomic** | **PD-MC** | **PD-NMC** | **Order taxonomic** | **PD-MC** | **PD-**  **NMC** | **Family taxonomic** | **PD-MC** | **PD-NMC** | **Genus taxonomic** | **PD-MC** | **PD-**  **NMC** | **species taxonomic** | **PD-MC** | **PD-NMC** |
| Firmicutes | 0.4372  11 | 0.4306  53 | Bacteroidia | 0.336744 | 0.338982 | Bacteroidales | 0.336662 | 0.3385  4 | Others | 0.3244  96 | 0.366148 | Others | 0.525175 | 0.5431  7 | Others | 0.801841 | 0.779933 |
| Bacteroidota | 0.3367  48 | 0.3389  82 | Clostridia | 0.26506 | 0.319143 | Lachnospirales | 0.119149 | 0.1398  96 | Bacteroidaceae | 0.2379  59 | 0.232068 | Bacteroides | 0.237959 | 0.2320  68 | Escherichia_coli | 0.04689 | 0.091809 |
| Proteobacteria | 0.1210  04 | 0.1544  33 | Gammaproteobacteri a | 0.120975 | 0.153682 | Others | 0.112652 | 0.1282  62 | Lachnospiraceae | 0.1190  48 | 0.139815 | Bifidobacterium | 0.057441 | 0.0227  34 | Klebsiella_pneumoniae | 0.04024 | 0.026675 |
| Actinobacteriota | 0.0699  22 | 0.0390  06 | Negativicutes | 0.08905 | 0.063501 | Oscillospirales | 0.110787 | 0.1218  05 | Enterobacteriaceae | 0.1064  26 | 0.131639 | Escherichia-Shigella | 0.047033 | 0.0920  59 | Bifidobacterium_longum | 0.023413 | 0.008414 |
| Verrucomicrobiota | 0.0191  22 | 0.0085  79 | Bacilli | 0.080918 | 0.047103 | Enterobacterales | 0.107225 | 0.1336  13 | Bifidobacteriaceae | 0.0574  72 | 0.022757 | Prevotella_9 | 0.046357 | 0.0515  94 | Bacteroides_fragilis | 0.021992 | 0.028013 |
| Fusobacteriota | 0.0056  21 | 0.0179  58 | Actinobacteria | 0.058249 | 0.023301 | Lactobacillales | 0.074195 | 0.0383  75 | Prevotellaceae | 0.0544  3 | 0.058875 | Megamonas | 0.023008 | 0.0063  03 | Lactobacillus_amylovorus | 0.018241 | 0.000046 |
| Desulfobacterota | 0.0052  47 | 0.0029  98 | Verrucomicrobiae | 0.019058 | 0.00857 | Veillonellales-  Selenomonadales | 0.068282 | 0.0451  06 | Lactobacillaceae | 0.0444  38 | 0.008488 | Lactobacillus | 0.021742 | 0.0001  75 | Enterococcus_faecium | 0.017873 | 0.014547 |
| Synergistota | 0.0042  3 | 0.0006  49 | Others | 0.012076 | 0.006262 | Bifidobacteriales | 0.057472 | 0.0227  57 | Selenomonadaceae | 0.0230  4 | 0.006773 | Blautia | 0.018086 | 0.0255  39 | Bacteroides_plebeius | 0.015798 | 0.026941 |
| Euryarchaeota | 0.0005  95 | 0.0058  08 | Coriobacteriia | 0.011654 | 0.01569 | Clostridia_UCG-014 | 0.007067 | 0.0161  6 | Enterococcaceae | 0.0179  87 | 0.014561 | Enterococcus | 0.017987 | 0.0145  61 | Collinsella_aerofaciens | 0.00874 | 0.011851 |
| Others | 0.0003 | 0.0004  97 | Fusobacteriia | 0.005621 | 0.017958 | Erysipelotrichales | 0.006106 | 0.0080  12 | Streptococcaceae | 0.0116  79 | 0.015193 | Citrobacter | 0.003943 | 0.0111  21 | Citrobacter_farmeri | 0.003943 | 0.011121 |
| Halobacterota | 0 | 0.0004  37 | Methanobacteria | 0.000595 | 0.005808 | Pseudomonadales | 0.000403 | 0.0074  74 | Erysipelatoclostridiaceae | 0.0030  25 | 0.003683 | Erysipelatoclostridium | 0.001269 | 0.0006  76 | Erysipelatoclostridium_ra  mosum | 0.001029 | 0.00065 |

**Supple Table 2: Lefse analysis of different taxonomics (related to Fig 3a ，b)**

| **Taxonomic** | **Group** | **LDA** | ***p*-value** | **taxonomic detail** |
| --- | --- | --- | --- | --- |
| p__Actinobacteriota | PD.MC | 4.262770514 | 0.009442196 | k__Bacteria.p__Actinobacteriota |
| c__Actinobacteria | PD.MC | 4.299700641 | 0.001762711 | k__Bacteria.p__Actinobacteriota.c__Actinobacteria |
| c__Rhodothermia | PD.MC | 3.609956744 | 0.025421566 | k__Bacteria.p__Bacteroidota.c__Rhodothermia |
| o__Bifidobacteriales | PD.MC | 4.295661298 | 0.002201062 | k__Bacteria.p__Actinobacteriota.c__Actinobacteria.o__Bifidobacteriales |
| o__Monoglobales | PD.NMC | 2.797116317 | 0.017344994 | k__Bacteria.p__Firmicutes.c__Clostridia.o__Monoglobales |
| o__Balneolales | PD.MC | 3.609560748 | 0.025421566 | k__Bacteria.p__Bacteroidota.c__Rhodothermia.o__Balneolales |
| f__Dietziaceae | PD.MC | 3.006055114 | 0.011214209 | k__Bacteria.p__Actinobacteriota.c__Actinobacteria.o__Corynebacteriales.f__Dietziaceae |
| f__Bifidobacteriaceae | PD.MC | 4.295661298 | 0.002201062 | k__Bacteria.p__Actinobacteriota.c__Actinobacteria.o__Bifidobacteriales.f__Bifidobacteriaceae |
| f__Balneolaceae | PD.MC | 3.323668247 | 0.025421566 | k__Bacteria.p__Bacteroidota.c__Rhodothermia.o__Balneolales.f__Balneolaceae |
| f__Pasteurellaceae | PD.NMC | 2.754903821 | 0.031334188 | k__Bacteria.p__Proteobacteria.c__Gammaproteobacteria.o__Enterobacterales.f__Pasteurellaceae |
| f__Monoglobaceae | PD.NMC | 2.797116317 | 0.017344994 | k__Bacteria.p__Firmicutes.c__Clostridia.o__Monoglobales.f__Monoglobaceae |

| f__Lactobacillaceae | PD.MC | 4.323882869 | 0.003191056 | k__Bacteria.p__Firmicutes.c__Bacilli.o__Lactobacillales.f__Lactobacillaceae |
| --- | --- | --- | --- | --- |
| g__Prevotella_7 | PD.NMC | 3.122064624 | 0.014809987 | k__Bacteria.p__Bacteroidota.c__Bacteroidia.o__Bacteroidales.f__Prevotellaceae.g__Prevotella_7 |
| g__Lactobacillus | PD.MC | 4.081608623 | 0.000199693 | k__Bacteria.p__Firmicutes.c__Bacilli.o__Lactobacillales.f__Lactobacillaceae.g__Lactobacillus |
| g__Haemophilus | PD.NMC | 2.790078272 | 0.037966207 | k__Bacteria.p__Proteobacteria.c__Gammaproteobacteria.o__Enterobacterales.f__Pasteurellaceae.g__Haemophilus |
| g__Blautia | PD.NMC | 3.66789237 | 0.010070072 | k__Bacteria.p__Firmicutes.c__Clostridia.o__Lachnospirales.f__Lachnospiraceae.g__Blautia |
| g__Ligilactobacillus | PD.MC | 3.986379867 | 0.002923335 | k__Bacteria.p__Firmicutes.c__Bacilli.o__Lactobacillales.f__Lactobacillaceae.g__Ligilactobacillus |
| g__Roseburia | PD.NMC | 3.301571614 | 0.035448078 | k__Bacteria.p__Firmicutes.c__Clostridia.o__Lachnospirales.f__Lachnospiraceae.g__Roseburia |
| g__Limosilactobacillus | PD.NMC | 3.284356634 | 0.001344964 | k__Bacteria.p__Firmicutes.c__Bacilli.o__Lactobacillales.f__Lactobacillaceae.g__Limosilactobacillus |
| g__Azoarcus | PD.MC | 2.31414913 | 0.045530259 | k__Bacteria.p__Proteobacteria.c__Gammaproteobacteria.o__Burkholderiales.f__Rhodocyclaceae.g__Azoarcus |
| g__Agathobacter | PD.NMC | 3.753265125 | 0.000439572 | k__Bacteria.p__Firmicutes.c__Clostridia.o__Lachnospirales.f__Lachnospiraceae.g__Agathobacter |
| g___Eubacterium__hallii_group | PD.NMC | 2.926455463 | 0.035622807 | k__Bacteria.p__Firmicutes.c__Clostridia.o__Lachnospirales.f__Lachnospiraceae.g___Eubacterium__hallii_group |
| g__Peptoniphilus | PD.NMC | 2.176556799 | 0.028761055 | k__Bacteria.p__Firmicutes.c__Clostridia.o__Peptostreptococcales_Tissierellales.f__Family_XI.g__Peptoniphilus |
| g__Lachnospiraceae_NK4A136_group | PD.NMC | 3.059698464 | 0.032833063 | k__Bacteria.p__Firmicutes.c__Clostridia.o__Lachnospirales.f__Lachnospiraceae.g__Lachnospiraceae_NK4A136_group |
| g__Bifidobacterium | PD.MC | 4.295589817 | 0.002766322 | k__Bacteria.p__Actinobacteriota.c__Actinobacteria.o__Bifidobacteriales.f__Bifidobacteriaceae.g__Bifidobacterium |
| g__Dietzia | PD.MC | 3.025961624 | 0.011214209 | k__Bacteria.p__Actinobacteriota.c__Actinobacteria.o__Corynebacteriales.f__Dietziaceae.g__Dietzia |
| g__Monoglobus | PD.NMC | 2.797116317 | 0.017344994 | k__Bacteria.p__Firmicutes.c__Clostridia.o__Monoglobales.f__Monoglobaceae.g__Monoglobus |
| s__Haemophilus_parainfluenzae | PD.NMC | 2.783152435 | 0.039238404 | k__Bacteria.p__Proteobacteria.c__Gammaproteobacteria.o__Enterobacterales.f__Pasteurellaceae.g__Haemophilus.s__Haemophilus_parainfluenzae |
| s__Lactobacillus_mucosae | PD.NMC | 3.390341895 | 0.011544885 | k__Bacteria.p__Firmicutes.c__Bacilli.o__Lactobacillales.f__Lactobacillaceae.g__Limosilactobacillus.s__Lactobacillus_mucosae |
| s__Lachnospiraceae_bacterium_GAM79 | PD.NMC | 3.016639824 | 0.033772326 | k__Bacteria.p__Firmicutes.c__Clostridia.o__Lachnospirales.f__Lachnospiraceae.g__Lachnospiraceae_NK4A136_group.s__Lachnospiraceae_bacterium_GAM79 |
| s__Bifidobacterium_adolescentis | PD.MC | 2.61200739 | 0.016032095 | k__Bacteria.p__Actinobacteriota.c__Actinobacteria.o__Bifidobacteriales.f__Bifidobacteriaceae.g__Bifidobacterium.s__Bifidobacterium_adolescentis |
| s__Lactobacillus_salivarius | PD.MC | 3.995164458 | 0.003341511 | k__Bacteria.p__Firmicutes.c__Bacilli.o__Lactobacillales.f__Lactobacillaceae.g__Ligilactobacillus.s__Lactobacillus_salivarius |
| s__Parabacteroides_gordonii | PD.NMC | 2.002140436 | 0.042028614 | k__Bacteria.p__Bacteroidota.c__Bacteroidia.o__Bacteroidales.f__Tannerellaceae.g__Parabacteroides.s__Parabacteroides_gordonii |
| s__Lactobacillus_amylovorus | PD.MC | 4.005990985 | 0.000249426 | k__Bacteria.p__Firmicutes.c__Bacilli.o__Lactobacillales.f__Lactobacillaceae.g__Lactobacillus.s__Lactobacillus_amylovorus |
| s__Dialister_sp_Marseille_P5638 | PD.MC | 3.50158337 | 0.026212631 | k__Bacteria.p__Firmicutes.c__Negativicutes.o__Veillonellales_Selenomonadales.f__Veillonellaceae.g__Dialister.s__Dialister_sp_ Marseille_P5638 |
| s__Lactobacillus_reuteri | PD.MC | 2.622103046 | 0.000332545 | k__Bacteria.p__Firmicutes.c__Bacilli.o__Lactobacillales.f__Lactobacillaceae.g__Limosilactobacillus.s__Lactobacillus_reuteri |
| s__iron_reducing_bacterium_enrichment_culture_clone_HN70 | PD.NMC | 2.29647294 | 0.03327597 | k__Bacteria.p__Firmicutes.c__Clostridia.o__Christensenellales.f__Christensenellaceae.g__Christensenellaceae_R_7_group .s__iron_reducing_bacterium_enrichment_culture_clone_HN70 |
| s__Bifidobacterium_longum | PD.MC | 3.938076694 | 0.042133973 | k__Bacteria.p__Actinobacteriota.c__Actinobacteria.o__Bifidobacteriales.f__Bifidobacteriaceae.g__Bifidobacterium.s__Bifidobacterium_longum |
| s__Lactobacillus_fermentum | PD.MC | 2.822931459 | 0.029270231 | k__Bacteria.p__Firmicutes.c__Bacilli.o__Lactobacillales.f__Lactobacillaceae.g__Limosilactobacillus.s__Lactobacillus_fermentum |

**Supple Table 3: Matastat analysis of different taxonomic groups (p <0.05)**

| **Genus taxonomic** | **Mean (PD-MC)** | **standard error (PD-MC)** | **Mean (PD-NMC)** | **standard error (PD-MC)** | ***p* value** | **q-value** | **taxonomic detail** |
| --- | --- | --- | --- | --- | --- | --- | --- |
| Escherichia-Shigella | 0.047033239 | 0.010488826 | 0.092058693 | 0.015988612 | 0.013986014 | 0.114219114 | k__Bacteria;p__Proteobacteria;c__Gammaproteobacteria;o__Enterobacterales;f__Enterobacteriaceae;g__Escherichia-Shigella; |
| Lactobacillus | 0.021742357 | 0.016310148 | 0.000175114 | 5.58E-05 | 0.001998002 | 0.020082482 | k__Bacteria;p__Firmicutes;c__Bacilli;o__Lactobacillales;f__Lactobacillaceae;g__Lactobacillus; |
| Lachnoclostridium | 0.007546478 | 0.001345676 | 0.013243989 | 0.002394243 | 0.033966034 | 0.21827353 | k__Bacteria;p__Firmicutes;c__Clostridia;o__Lachnospirales;f__Lachnospiraceae;g__Lachnoclostridium; |
| Agathobacter | 0.005019705 | 0.001987102 | 0.015867791 | 0.004054994 | 0.014985015 | 0.117482517 | k__Bacteria;p__Firmicutes;c__Clostridia;o__Lachnospirales;f__Lachnospiraceae;g__Agathobacter; |
| Lachnospiraceae_NK4A136_group | 0.002043504 | 0.000488492 | 0.004310273 | 0.000893403 | 0.026973027 | 0.182300458 | k__Bacteria;p__Firmicutes;c__Clostridia;o__Lachnospirales;f__Lachnospiraceae;g__Lachnospiraceae_NK4A136_group; |
| Holdemanella | 0.000439929 | 0.00025258 | 0.002061767 | 0.000791837 | 0.034965035 | 0.220579463 | k__Bacteria;p__Firmicutes;c__Bacilli;o__Erysipelotrichales;f__Erysipelotrichaceae;g__Holdemanella; |
| CAG-352 | 0 | 0 | 0.000740056 | 0.000720611 | 0.017982018 | 0.130536131 | k__Bacteria;p__Firmicutes;c__Clostridia;o__Oscillospirales;f__Ruminococcaceae;g__CAG-352; |
| Enterobacter | 0.003698136 | 0.001205733 | 0.001053953 | 0.000348384 | 0.036963037 | 0.222915546 | k__Bacteria;p__Proteobacteria;c__Gammaproteobacteria;o__Enterobacterales;f__Enterobacteriaceae;g__Enterobacter; |
| Prevotella_7 | 0 | 0 | 0.000722254 | 0.000700984 | 0.000999001 | 0.011887987 | k__Bacteria;p__Bacteroidota;c__Bacteroidia;o__Bacteroidales;f__Prevotellaceae;g__Prevotella_7; |
| [Ruminococcus]_gauvreauii_group | 0.000221449 | 7.17E-05 | 0.000603089 | 0.000201679 | 0.047952048 | 0.268087092 | k__Bacteria;p__Firmicutes;c__Clostridia;o__Lachnospirales;f__Lachnospiraceae;g__[Ruminococcus]_gauvreauii_group; |
| Lactonifactor | 0.000197701 | 8.23E-05 | 0.000851228 | 0.00026349 | 0.014985015 | 0.117482517 | k__Bacteria;p__Firmicutes;c__Clostridia;o__Lachnospirales;f__Lachnospiraceae;g__Lactonifactor; |
| Catenibacterium | 8.31E-06 | 8.31E-06 | 0.000599456 | 0.000458385 | 0.038961039 | 0.231404959 | k__Bacteria;p__Firmicutes;c__Bacilli;o__Erysipelotrichales;f__Erysipelatoclostridiaceae;g__Catenibacterium; |

| Paraclostridium | 0 | 0 | 2.22E-05 | 1.67E-05 | 2.60E-13 | 1.02E-10 | k__Bacteria;p__Firmicutes;c__Clostridia;o__Peptostreptococcales-Tissierellales;f__Peptostreptococcaceae;g__Paraclostridium; |
| --- | --- | --- | --- | --- | --- | --- | --- |
| [Eubacterium]_xylanophilum_group | 7.72E-06 | 4.99E-06 | 0.00022852 | 0.000131139 | 0.012987013 | 0.108317215 | k__Bacteria;p__Firmicutes;c__Clostridia;o__Lachnospirales;f__Lachnospiraceae;g__[Eubacterium]_xylanophilum_group; |
| unidentified_WPS-2 | 0.000161485 | 0.000161485 | 0 | 0 | 0.000999001 | 0.011887987 | k__Bacteria;p__WPS-2;c__unidentified_WPS-2;o__unidentified_WPS-2;f__unidentified_WPS-2;g__unidentified_WPS-2; |
| Anaerovorax | 0 | 0 | 6.90E-06 | 6.54E-06 | 0.000162941 | 0.00304157 | k__Bacteria;p__Firmicutes;c__Clostridia;o__Peptostreptococcales-Tissierellales;f__Anaerovoracaceae;g__Anaerovorax; |
| Lactococcus | 3.56E-06 | 2.18E-06 | 0.000207812 | 0.000115532 | 0.030969031 | 0.202331002 | k__Bacteria;p__Firmicutes;c__Bacilli;o__Lactobacillales;f__Streptococcaceae;g__Lactococcus; |
| TM7x | 2.20E-05 | 6.79E-06 | 0.000181653 | 0.000101802 | 0.042957043 | 0.251330759 | k__Bacteria;p__Patescibacteria;c__Saccharimonadia;o__Saccharimonadales;f__Saccharimonadaceae;g__TM7x; |
| Epulopiscium | 1.66E-05 | 9.97E-06 | 0 | 0 | 1.67E-12 | 2.78E-10 | k__Bacteria;p__Firmicutes;c__Clostridia;o__Lachnospirales;f__Lachnospiraceae;g__Epulopiscium; |
| Asteroleplasma | 0 | 0 | 2.62E-05 | 2.62E-05 | 0.000999001 | 0.011887987 | k__Bacteria;p__Firmicutes;c__Bacilli;o__Erysipelotrichales;f__Erysipelatoclostridiaceae;g__Asteroleplasma; |
| Companilactobacillus | 9.50E-06 | 9.50E-06 | 0 | 0 | 1.86E-07 | 6.08E-06 | k__Bacteria;p__Firmicutes;c__Bacilli;o__Lactobacillales;f__Lactobacillaceae;g__Companilactobacillus; |
| Coriobacteriaceae_UCG-002 | 5.94E-07 | 5.94E-07 | 2.33E-05 | 2.33E-05 | 2.12E-12 | 2.78E-10 | k__Bacteria;p__Actinobacteriota;c__Coriobacteriia;o__Coriobacteriales;f__Atopobiaceae;g__Coriobacteriaceae_UCG-002; |
| W5053 | 1.78E-06 | 1.78E-06 | 2.18E-05 | 2.11E-05 | 1.60E-09 | 7.85E-08 | k__Bacteria;p__Firmicutes;c__Clostridia;o__Peptostreptococcales-Tissierellales;f__Family_XI;g__W5053; |
| Paucilactobacillus | 3.27E-05 | 3.09E-05 | 0 | 0 | 0.000999001 | 0.011887987 | k__Bacteria;p__Firmicutes;c__Bacilli;o__Lactobacillales;f__Lactobacillaceae;g__Paucilactobacillus; |
| Dysgonomonas | 3.03E-05 | 3.03E-05 | 0 | 0 | 0.000999001 | 0.011887987 | k__Bacteria;p__Bacteroidota;c__Bacteroidia;o__Bacteroidales;f__Dysgonomonadaceae;g__Dysgonomonas; |
| Catenisphaera | 0 | 0 | 1.78E-05 | 1.78E-05 | 1.33E-10 | 8.66E-09 | k__Bacteria;p__Firmicutes;c__Bacilli;o__Erysipelotrichales;f__Erysipelotrichaceae;g__Catenisphaera; |
| Enterorhabdus | 0 | 0 | 1.82E-05 | 1.71E-05 | 7.15E-11 | 5.60E-09 | k__Bacteria;p__Actinobacteriota;c__Coriobacteriia;o__Coriobacteriales;f__Eggerthellaceae;g__Enterorhabdus; |
| Arcobacter | 0 | 0 | 1.60E-05 | 1.60E-05 | 1.17E-09 | 6.54E-08 | k__Bacteria;p__Campylobacterota;c__Campylobacteria;o__Campylobacterales;f__Arcobacteraceae;g__Arcobacter; |
| Zoogloea | 0 | 0 | 1.38E-05 | 1.38E-05 | 1.93E-08 | 8.41E-07 | k__Bacteria;p__Proteobacteria;c__Gammaproteobacteria;o__Burkholderiales;f__Rhodocyclaceae;g__Zoogloea; |
| Neisseria | 8.91E-06 | 7.20E-06 | 1.45E-06 | 7.10E-07 | 0.000444376 | 0.006752479 | k__Bacteria;p__Proteobacteria;c__Gammaproteobacteria;o__Burkholderiales;f__Neisseriaceae;g__Neisseria; |
| Aeromonas | 0 | 0 | 1.27E-05 | 1.27E-05 | 6.32E-08 | 2.25E-06 | k__Bacteria;p__Proteobacteria;c__Gammaproteobacteria;o__Enterobacterales;f__Aeromonadaceae;g__Aeromonas; |
| Scardovia | 5.94E-06 | 2.37E-06 | 1.31E-05 | 6.68E-06 | 0.022794014 | 0.162459158 | k__Bacteria;p__Actinobacteriota;c__Actinobacteria;o__Bifidobacteriales;f__Bifidobacteriaceae;g__Scardovia; |
| Enhydrobacter | 2.02E-05 | 1.96E-05 | 1.09E-06 | 1.09E-06 | 9.76E-12 | 9.57E-10 | k__Bacteria;p__Proteobacteria;c__Gammaproteobacteria;o__Pseudomonadales;f__Moraxellaceae;g__Enhydrobacter; |
| Parapusillimonas | 0 | 0 | 1.09E-05 | 1.09E-05 | 1.05E-06 | 2.75E-05 | k__Bacteria;p__Proteobacteria;c__Gammaproteobacteria;o__Burkholderiales;f__Alcaligenaceae;g__Parapusillimonas; |
| Harryflintia | 1.25E-05 | 5.52E-06 | 2.40E-05 | 1.43E-05 | 0.007768601 | 0.070820731 | k__Bacteria;p__Firmicutes;c__Clostridia;o__Oscillospirales;f__Ruminococcaceae;g__Harryflintia; |
| Ruminiclostridium | 2.20E-05 | 1.68E-05 | 8.36E-06 | 4.59E-06 | 0.000257451 | 0.004387868 | k__Bacteria;p__Firmicutes;c__Clostridia;o__Oscillospirales;f__Hungateiclostridiaceae;g__Ruminiclostridium; |
| Leptotrichia | 5.94E-07 | 5.94E-07 | 1.13E-05 | 1.02E-05 | 9.08E-06 | 0.000222554 | k__Bacteria;p__Fusobacteriota;c__Fusobacteriia;o__Fusobacteriales;f__Leptotrichiaceae;g__Leptotrichia; |
| Sanguibacteroides | 1.42E-05 | 1.42E-05 | 1.82E-06 | 1.49E-06 | 9.94E-07 | 2.75E-05 | k__Bacteria;p__Bacteroidota;c__Bacteroidia;o__Bacteroidales;f__Marinifilaceae;g__Sanguibacteroides; |
| Arthrobacter | 0 | 0 | 7.99E-06 | 7.99E-06 | 3.15E-05 | 0.000686939 | k__Bacteria;p__Actinobacteriota;c__Actinobacteria;o__Micrococcales;f__Micrococcaceae;g__Arthrobacter; |
| Succinatimonas | 2.08E-05 | 1.50E-05 | 1.09E-05 | 5.93E-06 | 0.010244027 | 0.089236861 | k__Bacteria;p__Proteobacteria;c__Gammaproteobacteria;o__Enterobacterales;f__Succinivibrionaceae;g__Succinatimonas; |
| unidentified_Erysipelotrichacea | 1.48E-05 | 1.08E-05 | 7.99E-06 | 6.93E-06 | 0.035450271 | 0.220579463 | k__Bacteria;p__Firmicutes;c__Bacilli;o__Erysipelotrichales;f__Erysipelotrichaceae;g__unidentified_Erysipelotrichaceae; |
| Moryella | 0 | 0 | 6.90E-06 | 6.54E-06 | 0.000162941 | 0.00304157 | k__Bacteria;p__Firmicutes;c__Clostridia;o__Lachnospirales;f__Lachnospiraceae;g__Moryella; |
| Schleiferilactobacillus | 1.07E-05 | 1.07E-05 | 0 | 0 | 2.68E-08 | 1.05E-06 | k__Bacteria;p__Firmicutes;c__Bacilli;o__Lactobacillales;f__Lactobacillaceae;g__Schleiferilactobacillus; |
| Ralstonia | 1.07E-05 | 1.01E-05 | 3.63E-07 | 3.63E-07 | 3.26E-07 | 9.84E-06 | k__Bacteria;p__Proteobacteria;c__Gammaproteobacteria;o__Burkholderiales;f__Burkholderiaceae;g__Ralstonia; |
| Macrococcus | 0 | 0 | 6.18E-06 | 5.82E-06 | 0.00053592 | 0.007780764 | k__Bacteria;p__Firmicutes;c__Bacilli;o__Staphylococcales;f__Staphylococcaceae;g__Macrococcus; |
| Gardnerella | 1.13E-05 | 9.55E-06 | 4.72E-06 | 4.02E-06 | 0.016846924 | 0.12699989 | k__Bacteria;p__Actinobacteriota;c__Actinobacteria;o__Bifidobacteriales;f__Bifidobacteriaceae;g__Gardnerella; |
| hgcI_clade | 1.13E-05 | 9.04E-06 | 3.27E-06 | 2.13E-06 | 0.001546015 | 0.016900602 | k__Bacteria;p__Actinobacteriota;c__Actinobacteria;o__Frankiales;f__Sporichthyaceae;g__hgcI_clade; |
| Alicycliphilus | 1.19E-06 | 1.19E-06 | 5.45E-06 | 5.45E-06 | 0.025142107 | 0.175994746 | k__Bacteria;p__Proteobacteria;c__Gammaproteobacteria;o__Burkholderiales;f__Comamonadaceae;g__Alicycliphilus; |
| Oribacterium | 5.34E-06 | 2.17E-06 | 1.16E-05 | 3.95E-06 | 0.036399557 | 0.222915546 | k__Bacteria;p__Firmicutes;c__Clostridia;o__Lachnospirales;f__Lachnospiraceae;g__Oribacterium; |
| Tumebacillus | 0 | 0 | 5.09E-06 | 5.09E-06 | 0.00159521 | 0.016900602 | k__Bacteria;p__Firmicutes;c__Bacilli;o__Alicyclobacillales;f__Alicyclobacillaceae;g__Tumebacillus; |
| Conexibacter | 0 | 0 | 5.09E-06 | 5.09E-06 | 0.00159521 | 0.016900602 | k__Bacteria;p__Actinobacteriota;c__Thermoleophilia;o__Solirubrobacterales;f__Solirubrobacteraceae;g__Conexibacter; |
| Providencia | 5.94E-07 | 5.94E-07 | 5.45E-06 | 4.77E-06 | 0.00812857 | 0.07241817 | k__Bacteria;p__Proteobacteria;c__Gammaproteobacteria;o__Enterobacterales;f__Morganellaceae;g__Providencia; |
| Bacillus | 2.02E-05 | 6.41E-06 | 1.05E-05 | 4.55E-06 | 0.012952193 | 0.108317215 | k__Bacteria;p__Firmicutes;c__Bacilli;o__Bacillales;f__Bacillaceae;g__Bacillus; |
| Empedobacter | 5.94E-06 | 5.94E-06 | 0 | 0 | 6.22E-05 | 0.001282704 | k__Bacteria;p__Bacteroidota;c__Bacteroidia;o__Flavobacteriales;f__Weeksellaceae;g__Empedobacter; |
| Dietzia | 1.13E-05 | 5.77E-06 | 1.45E-06 | 1.02E-06 | 1.51E-05 | 0.000349099 | k__Bacteria;p__Actinobacteriota;c__Actinobacteria;o__Corynebacteriales;f__Dietziaceae;g__Dietzia; |

| Sphaerochaeta | 0 | 0 | 5.81E-06 | 3.91E-06 | 0.001000774 | 0.011887987 | k__Bacteria;p__Spirochaetota;c__Spirochaetia;o__Spirochaetales;f__Spirochaetaceae;g__Sphaerochaeta; |
| --- | --- | --- | --- | --- | --- | --- | --- |
| Anaerofustis | 1.60E-05 | 6.78E-06 | 7.99E-06 | 2.76E-06 | 0.017721786 | 0.130536131 | k__Bacteria;p__Firmicutes;c__Clostridia;o__Eubacteriales;f__Anaerofustaceae;g__Anaerofustis; |
| Latilactobacillus | 0 | 0 | 2.91E-06 | 2.91E-06 | 0.028009624 | 0.186097844 | k__Bacteria;p__Firmicutes;c__Bacilli;o__Lactobacillales;f__Lactobacillaceae;g__Latilactobacillus; |
| CL500-29_marine_group | 7.12E-06 | 4.97E-06 | 1.09E-06 | 1.09E-06 | 0.001899151 | 0.019591239 | k__Bacteria;p__Actinobacteriota;c__Acidimicrobiia;o__Microtrichales;f__Ilumatobacteraceae;g__CL500-29_marine_group; |
| Alcanivorax | 1.72E-05 | 6.32E-06 | 5.45E-06 | 1.99E-06 | 0.00024708 | 0.004387868 | k__Bacteria;p__Proteobacteria;c__Gammaproteobacteria;o__Pseudomonadales;f__Alcanivoracaceae1;g__Alcanivorax; |
| Alloscardovia | 1.13E-05 | 5.77E-06 | 2.54E-06 | 1.88E-06 | 0.000366543 | 0.005986862 | k__Bacteria;p__Actinobacteriota;c__Actinobacteria;o__Bifidobacteriales;f__Bifidobacteriaceae;g__Alloscardovia; |
| UCG-007 | 9.50E-06 | 5.37E-06 | 3.63E-06 | 2.02E-06 | 0.015552361 | 0.119539714 | k__Bacteria;p__Firmicutes;c__Clostridia;o__Oscillospirales;f__Oscillospiraceae;g__UCG-007; |
| Williamsia | 4.16E-06 | 4.16E-06 | 0 | 0 | 0.001136364 | 0.013101605 | k__Bacteria;p__Actinobacteriota;c__Actinobacteria;o__Corynebacteriales;f__Nocardiaceae;g__Williamsia; |
| unidentified_Gastranaerophilales | 0 | 0 | 2.54E-06 | 2.54E-06 | 0.04949887 | 0.268087092 | k__Bacteria;p__Cyanobacteria;c__Vampirivibrionia;o__Gastranaerophilales;f__unidentified_Gastranaerophilales;g__unidentified_Gastranaerophilales; |
| Cryptobacterium | 6.53E-06 | 2.69E-06 | 1.45E-06 | 1.45E-06 | 0.00667107 | 0.062263322 | k__Bacteria;p__Actinobacteriota;c__Coriobacteriia;o__Coriobacteriales;f__Eggerthellaceae;g__Cryptobacterium; |
| Pseudoramibacter | 4.75E-06 | 3.73E-06 | 1.09E-06 | 1.09E-06 | 0.026147146 | 0.179818966 | k__Bacteria;p__Firmicutes;c__Clostridia;o__Eubacteriales;f__Eubacteriaceae;g__Pseudoramibacter; |
| Lentimicrobium | 0 | 0 | 2.54E-06 | 1.88E-06 | 0.04949887 | 0.268087092 | k__Bacteria;p__Bacteroidota;c__Bacteroidia;o__Sphingobacteriales;f__Lentimicrobiaceae;g__Lentimicrobium; |
| Solirubrobacter | 5.94E-06 | 3.78E-06 | 3.63E-07 | 3.63E-07 | 0.000447868 | 0.006752479 | k__Bacteria;p__Actinobacteriota;c__Thermoleophilia;o__Solirubrobacterales;f__Solirubrobacteraceae;g__Solirubrobacter; |
| Chelativorans | 1.07E-05 | 3.81E-06 | 5.09E-06 | 1.83E-06 | 0.043682942 | 0.251819312 | k__Bacteria;p__Proteobacteria;c__Alphaproteobacteria;o__Rhizobiales;f__Rhizobiaceae;g__Chelativorans; |
| Rubellimicrobium | 3.56E-06 | 2.49E-06 | 0 | 0 | 0.002993355 | 0.029334878 | k__Bacteria;p__Proteobacteria;c__Alphaproteobacteria;o__Rhodobacterales;f__Rhodobacteraceae;g__Rubellimicrobium; |
| Pseudopropionibacterium | 0 | 0 | 2.54E-06 | 1.05E-06 | 0.04949887 | 0.268087092 | k__Bacteria;p__Actinobacteriota;c__Actinobacteria;o__Propionibacteriales;f__Propionibacteriaceae;g__Pseudopropionibacterium; |
| Azoarcus | 4.16E-06 | 2.07E-06 | 3.63E-07 | 3.63E-07 | 0.006071136 | 0.058045984 | k__Bacteria;p__Proteobacteria;c__Gammaproteobacteria;o__Burkholderiales;f__Rhodocyclaceae;g__Azoarcus; |
| Blautia | 0.018086373 | 0.038343752 | 0.025538657 | 0.039256451 | 0.010255621 | 0.227489436 | k__Bacteria;p__Firmicutes;c__Clostridia;o__Lachnospirales;f__Lachnospiraceae;g__Blautia; |
| [Eubacterium]_hallii_group | 0.00390118 | 0.009305077 | 0.00421218 | 0.007179094 | 0.034513172 | 0.376145294 | k__Bacteria;p__Firmicutes;c__Clostridia;o__Lachnospirales;f__Lachnospiraceae;g__[Eubacterium]_hallii_group; |

**Supple Table 4: Random Forest Mean Decrease Accuracy top 30 genus taxonomic（ related to Fig 3d）**

| **Genus taxonomic** | **Mean Decrease Accuracy** | **taxonomic detail** |
| --- | --- | --- |
| Lactobacillus | 30.25194729 | k__Bacteria;p__Firmicutes;c__Bacilli;o__Lactobacillales;f__Lactobacillaceae;g__Lactobacillus; |
| Blautia | 21.24463225 | k__Bacteria;p__Firmicutes;c__Clostridia;o__Lachnospirales;f__Lachnospiraceae;g__Blautia; |
| Agathobacter | 19.50318842 | k__Bacteria;p__Firmicutes;c__Clostridia;o__Lachnospirales;f__Lachnospiraceae;g__Agathobacter; |
| Bifidobacterium | 14.74319459 | k__Bacteria;p__Actinobacteriota;c__Actinobacteria;o__Bifidobacteriales;f__Bifidobacteriaceae;g__Bifidobacterium; |
| Limosilactobacillus | 13.84685867 | k__Bacteria;p__Firmicutes;c__Bacilli;o__Lactobacillales;f__Lactobacillaceae;g__Limosilactobacillus; |
| Ligilactobacillus | 12.99252572 | k__Bacteria;p__Firmicutes;c__Bacilli;o__Lactobacillales;f__Lactobacillaceae;g__Ligilactobacillus; |
| Bacillus | 12.00113253 | k__Bacteria;p__Firmicutes;c__Bacilli;o__Bacillales;f__Bacillaceae;g__Bacillus; |
| Roseburia | 9.325657041 | k__Bacteria;p__Firmicutes;c__Clostridia;o__Lachnospirales;f__Lachnospiraceae;g__Roseburia; |
| Aliidiomarina | 8.740180079 | k__Bacteria;p__Proteobacteria;c__Gammaproteobacteria;o__Enterobacterales;f__Idiomarinaceae;g__Aliidiomarina; |
| Halomonas | 8.70264395 | k__Bacteria;p__Proteobacteria;c__Gammaproteobacteria;o__Pseudomonadales;f__Halomonadaceae;g__Halomonas; |
| Bilophila | 7.414578727 | k__Bacteria;p__Desulfobacterota;c__Desulfovibrionia;o__Desulfovibrionales;f__Desulfovibrionaceae;g__Bilophila; |
| [Eubacterium]_hallii_group | 7.015746156 | k__Bacteria;p__Firmicutes;c__Clostridia;o__Lachnospirales;f__Lachnospiraceae;g__[Eubacterium]_hallii_group; |
| Cronobacter | 6.966891262 | k__Bacteria;p__Proteobacteria;c__Gammaproteobacteria;o__Enterobacterales;f__Enterobacteriaceae;g__Cronobacter; |
| Holdemanella | 6.784771539 | k__Bacteria;p__Firmicutes;c__Bacilli;o__Erysipelotrichales;f__Erysipelotrichaceae;g__Holdemanella; |
| UCG-003 | 6.244414101 | k__Bacteria;p__Firmicutes;c__Clostridia;o__Oscillospirales;f__Oscillospiraceae;g__UCG-003; |
| Chelativorans | 6.186662286 | k__Bacteria;p__Proteobacteria;c__Alphaproteobacteria;o__Rhizobiales;f__Rhizobiaceae;g__Chelativorans; |
| Epulopiscium | 6.155814477 | k__Bacteria;p__Firmicutes;c__Clostridia;o__Lachnospirales;f__Lachnospiraceae;g__Epulopiscium; |
| CAG_56 | 6.076204578 | k__Bacteria;p__Firmicutes;c__Clostridia;o__Lachnospirales;f__Lachnospiraceae;g__CAG-56; |
| Erysipelotrichaceae_UCG-003 | 5.810852372 | k__Bacteria;p__Firmicutes;c__Bacilli;o__Erysipelotrichales;f__Erysipelatoclostridiaceae;g__Erysipelotrichaceae_UCG-003; |
| Alcanivorax | 5.580824146 | k__Bacteria;p__Proteobacteria;c__Gammaproteobacteria;o__Pseudomonadales;f__Alcanivoracaceae1;g__Alcanivorax; |
| Merdibacter | 5.41876591 | k__Bacteria;p__Firmicutes;c__Bacilli;o__Erysipelotrichales;f__Erysipelotrichaceae;g__Merdibacter; |

| Akkermansia | 5.148023914 | k__Bacteria;p__Verrucomicrobiota;c__Verrucomicrobiae;o__Verrucomicrobiales;f__Akkermansiaceae;g__Akkermansia; |
| --- | --- | --- |
| Cryptobacterium | 5.139608313 | k__Bacteria;p__Actinobacteriota;c__Coriobacteriia;o__Coriobacteriales;f__Eggerthellaceae;g__Cryptobacterium; |
| Prevotella_7 | 4.959366384 | k__Bacteria;p__Bacteroidota;c__Bacteroidia;o__Bacteroidales;f__Prevotellaceae;g__Prevotella_7; |
| Veillonella | 4.80756779 | k__Bacteria;p__Firmicutes;c__Negativicutes;o__Veillonellales-Selenomonadales;f__Veillonellaceae;g__Veillonella; |
| Fusicatenibacter | 4.730897462 | k__Bacteria;p__Firmicutes;c__Clostridia;o__Lachnospirales;f__Lachnospiraceae;g__Fusicatenibacter; |
| F0332 | 4.729537544 | k__Bacteria;p__Actinobacteriota;c__Actinobacteria;o__Actinomycetales;f__Actinomycetaceae;g__F0332; |
| Lachnospiraceae_FCS020_group | 4.678496581 | k__Bacteria;p__Firmicutes;c__Clostridia;o__Lachnospirales;f__Lachnospiraceae;g__Lachnospiraceae_FCS020_group; |
| Sutterella | 4.621904368 | k__Bacteria;p__Proteobacteria;c__Gammaproteobacteria;o__Burkholderiales;f__Sutterellaceae;g__Sutterella; |
| UCG_002 | 4.485798742 | k__Bacteria;p__Firmicutes;c__Clostridia;o__Oscillospirales;f__Oscillospiraceae;g__UCG-002; |

**Supple Table 5: Random Forest Mean Decrease Gini top 30 genus taxonomic (related to Fig 3d)**

| **Genus taxonomic** | **Mean Decrease Gini** | **taxonomic detail** |
| --- | --- | --- |
| Lactobacillus | 1.355598166 | k__Bacteria;p__Firmicutes;c__Bacilli;o__Lactobacillales;f__Lactobacillaceae;g__Lactobacillus; |
| Blautia | 0.934755108 | k__Bacteria;p__Firmicutes;c__Clostridia;o__Lachnospirales;f__Lachnospiraceae;g__Blautia; |
| Agathobacter | 0.754488912 | k__Bacteria;p__Firmicutes;c__Clostridia;o__Lachnospirales;f__Lachnospiraceae;g__Agathobacter; |
| Bifidobacterium | 0.686669796 | k__Bacteria;p__Actinobacteriota;c__Actinobacteria;o__Bifidobacteriales;f__Bifidobacteriaceae;g__Bifidobacterium; |
| Ligilactobacillus | 0.516342664 | k__Bacteria;p__Firmicutes;c__Bacilli;o__Lactobacillales;f__Lactobacillaceae;g__Ligilactobacillus; |
| Limosilactobacillus | 0.50886112 | k__Bacteria;p__Firmicutes;c__Bacilli;o__Lactobacillales;f__Lactobacillaceae;g__Limosilactobacillus; |
| Roseburia | 0.417906115 | k__Bacteria;p__Firmicutes;c__Clostridia;o__Lachnospirales;f__Lachnospiraceae;g__Roseburia; |
| [Eubacterium]_hallii_group | 0.368126896 | k__Bacteria;p__Firmicutes;c__Clostridia;o__Lachnospirales;f__Lachnospiraceae;g__[Eubacterium]_hallii_group; |
| Bacillus | 0.334200385 | k__Bacteria;p__Firmicutes;c__Bacilli;o__Bacillales;f__Bacillaceae;g__Bacillus; |
| Escherichia-Shigella | 0.308307786 | k__Bacteria;p__Proteobacteria;c__Gammaproteobacteria;o__Enterobacterales;f__Enterobacteriaceae;g__Escherichia-Shigella; |
| Halomonas | 0.302002087 | k__Bacteria;p__Proteobacteria;c__Gammaproteobacteria;o__Pseudomonadales;f__Halomonadaceae;g__Halomonas; |
| Bilophila | 0.284672512 | k__Bacteria;p__Desulfobacterota;c__Desulfovibrionia;o__Desulfovibrionales;f__Desulfovibrionaceae;g__Bilophila; |
| Romboutsia | 0.262593204 | k__Bacteria;p__Firmicutes;c__Clostridia;o__Peptostreptococcales-Tissierellales;f__Peptostreptococcaceae;g__Romboutsia; |
| Lachnoclostridium | 0.257785184 | k__Bacteria;p__Firmicutes;c__Clostridia;o__Lachnospirales;f__Lachnospiraceae;g__Lachnoclostridium; |
| Oscillibacter | 0.255236839 | k__Bacteria;p__Firmicutes;c__Clostridia;o__Oscillospirales;f__Oscillospiraceae;g__Oscillibacter; |
| CAG_56 | 0.246709776 | k__Bacteria;p__Firmicutes;c__Clostridia;o__Lachnospirales;f__Lachnospiraceae;g__CAG-56; |
| Megasphaera | 0.245985324 | k__Bacteria;p__Firmicutes;c__Negativicutes;o__Veillonellales-Selenomonadales;f__Veillonellaceae;g__Megasphaera; |
| Lachnospiraceae_NK4A136_group | 0.235007888 | k__Bacteria;p__Firmicutes;c__Clostridia;o__Lachnospirales;f__Lachnospiraceae;g__Lachnospiraceae_NK4A136_group; |
| Erysipelotrichaceae_UCG_003 | 0.232750141 | k__Bacteria;p__Firmicutes;c__Bacilli;o__Erysipelotrichales;f__Erysipelatoclostridiaceae;g__Erysipelotrichaceae_UCG-003; |
| Akkermansia | 0.224163389 | k__Bacteria;p__Verrucomicrobiota;c__Verrucomicrobiae;o__Verrucomicrobiales;f__Akkermansiaceae;g__Akkermansia; |
| Acidaminococcus | 0.223517865 | k__Bacteria;p__Firmicutes;c__Negativicutes;o__Acidaminococcales;f__Acidaminococcaceae;g__Acidaminococcus; |
| Aliidiomarina | 0.221652235 | k__Bacteria;p__Proteobacteria;c__Gammaproteobacteria;o__Enterobacterales;f__Idiomarinaceae;g__Aliidiomarina; |
| [Eubacterium]_ventriosum_group | 0.217283723 | k__Bacteria;p__Firmicutes;c__Clostridia;o__Lachnospirales;f__Lachnospiraceae;g__[Eubacterium]_ventriosum_group; |
| Lachnospiraceae_FCS020_group | 0.216372157 | k__Bacteria;p__Firmicutes;c__Clostridia;o__Lachnospirales;f__Lachnospiraceae;g__Lachnospiraceae_FCS020_group; |
| Haemophilus | 0.216272106 | k__Bacteria;p__Proteobacteria;c__Gammaproteobacteria;o__Enterobacterales;f__Pasteurellaceae;g__Haemophilus; |
| Cronobacter | 0.211294054 | k__Bacteria;p__Proteobacteria;c__Gammaproteobacteria;o__Enterobacterales;f__Enterobacteriaceae;g__Cronobacter; |
| Fusicatenibacter | 0.202795095 | k__Bacteria;p__Firmicutes;c__Clostridia;o__Lachnospirales;f__Lachnospiraceae;g__Fusicatenibacter; |
| UCG-003 | 0.194961866 | k__Bacteria;p__Firmicutes;c__Clostridia;o__Oscillospirales;f__Oscillospiraceae;g__UCG-003; |
| Enterobacter | 0.190412546 | k__Bacteria;p__Proteobacteria;c__Gammaproteobacteria;o__Enterobacterales;f__Enterobacteriaceae;g__Enterobacter; |
| Enterococcus | 0.188539164 | k__Bacteria;p__Firmicutes;c__Bacilli;o__Lactobacillales;f__Enterococcaceae;g__Enterococcus; |

**Supple Table 6: Single AUC analysis at the genus level (related to Fig 3e)**

| **Genus taxonomic** | **merge** | **AUC** | ***p* value** | **q-value** | **phylus** | **taxonomic detai** |
| --- | --- | --- | --- | --- | --- | --- |
| Lactobacillus | 4 | 0.699 | 0.001998002 | 0.020082482 | Firmicutes | k__Bacteria;p__Firmicutes;c__Bacilli;o__Lactobacillales;f__Lactobacillaceae;g__Lactobacillus; |
| Agathobacter | 4 | 0.702 | 0.014985015 | 0.117482517 | Firmicutes | k__Bacteria;p__Firmicutes;c__Clostridia;o__Lachnospirales;f__Lachnospiraceae;g__Agathobacter; |
| Prevotella_7 | 3 | 0.567 | 0.000999001 | 0.011887987 | Bacteroidota | k _Bacteria;p__Bacteroidota;c__Bacteroidia;o__Bacteroidales;f__Prevotellaceae;g__Prevotella_7; |
| Blautia | 3 | 0.648 | 0.010255621 | 0.227489436 | Firmicutes | k__Bacteria;p__Firmicutes;c__Clostridia;o__Lachnospirales;f__Lachnospiraceae;g__Blautia; |
| Lachnospiraceae_NK4A136_group | 3 | 0.622 | 0.026973027 | 0.182300458 | Firmicutes | k__Bacteria;p__Firmicutes;c__Clostridia;o__Lachnospirales;f__Lachnospiraceae;g__Lachnospiraceae_NK4A136_group; |
| Bacillus | 3 | 0.570 | 0.012952193 | 0.108317215 | Firmicutes | k__Bacteria;p__Firmicutes;c__Bacilli;o__Bacillales;f__Bacillaceae;g__Bacillus; |
| Ligilactobacillus | 3 | 0.666 | 0.052947053 | 0.268087092 | Firmicutes | k__Bacteria;p__Firmicutes;c__Bacilli;o__Lactobacillales;f__Lactobacillaceae;g__Ligilactobacillus; |
| Roseburia | 3 | 0.620 | 0.602397602 | 0.906250837 | Firmicutes | k__Bacteria;p__Firmicutes;c__Clostridia;o__Lachnospirales;f__Lachnospiraceae;g__Roseburia; |
| Limosilactobacillus | 3 | 0.679 | 0.775224775 | 0.994378171 | Firmicutes | k__Bacteria;p__Firmicutes;c__Bacilli;o__Lactobacillales;f__Lactobacillaceae;g__Limosilactobacillus; |
| Bifidobacterium | 3 | 0.672 | 0.067932068 | 0.302606484 | Actinobacteriota | k__Bacteria;p__Actinobacteriota;c__Actinobacteria;o__Bifidobacteriales;f__Bifidobacteriaceae;g__Bifidobacterium; |
| Lachnoclostridium | 2 | 0.595 | 0.033966034 | 0.21827353 | Firmicutes | k__Bacteria;p__Firmicutes;c__Clostridia;o__Lachnospirales;f__Lachnospiraceae;g__Lachnoclostridium; |
| Holdemanella | 2 | 0.570 | 0.034965035 | 0.220579463 | Firmicutes | k__Bacteria;p__Firmicutes;c__Bacilli;o__Erysipelotrichales;f__Erysipelotrichaceae;g__Holdemanella; |
| Enterobacter | 2 | 0.511 | 0.036963037 | 0.222915546 | Proteobacteria | k__Bacteria;p__Proteobacteria;c__Gammaproteobacteria;o__Enterobacterales;f__Enterobacteriaceae;g__Enterobacter; |
| Epulopiscium | 2 | 0.549 | 1.67E-12 | 2.78E-10 | Firmicutes | k__Bacteria;p__Firmicutes;c__Clostridia;o__Lachnospirales;f__Lachnospiraceae;g__Epulopiscium; |
| Dietzia | 2 | 0.570 | 1.51E-05 | 0.000349099 | Actinobacteriota | k__Bacteria;p__Actinobacteriota;c__Actinobacteria;o__Corynebacteriales;f__Dietziaceae;g__Dietzia; |
| Alcanivorax | 2 | 0.561 | 0.00024708 | 0.004387868 | Proteobacteria | k__Bacteria;p__Proteobacteria;c__Gammaproteobacteria;o__Pseudomonadales;f__Alcanivoracaceae1;g__Alcanivorax; |
| Cryptobacterium | 2 | 0.565 | 0.00667107 | 0.062263322 | Actinobacteriota | k__Bacteria;p__Actinobacteriota;c__Coriobacteriia;o__Coriobacteriales;f__Eggerthellaceae;g__Cryptobacterium; |
| Chelativorans | 2 | 0.541 | 0.043682942 | 0.251819312 | Proteobacteria | k__Bacteria;p__Proteobacteria;c__Alphaproteobacteria;o__Rhizobiales;f__Rhizobiaceae;g__Chelativorans; |
| Azoarcus | 2 | 0.542 | 0.006071136 | 0.058045984 | Proteobacteria | k__Bacteria;p__Proteobacteria;c__Gammaproteobacteria;o__Burkholderiales;f__Rhodocyclaceae;g__Azoarcus; |
| Haemophilus | 2 | 0.614 | 0.618381618 | 0.911299227 | Proteobacteria | k__Bacteria;p__Proteobacteria;c__Gammaproteobacteria;o__Enterobacterales;f__Pasteurellaceae;g__Haemophilus; |
| Aliidiomarina | 2 | 0.587 | 0.092907093 | 0.375459592 | Proteobacteria | __Bacteria;p__Proteobacteria;c__Gammaproteobacteria;o__Enterobacterales;f__Idiomarinaceae;g__Aliidiomarina; |
| Halomonas | 2 | 0.600 | 0.140859141 | 0.496262358 | Proteobacteria | k__Bacteria;p__Proteobacteria;c__Gammaproteobacteria;o__Pseudomonadales;f__Halomonadaceae;g__Halomonas; |
| Bilophila | 2 | 0.603 | 0.271728272 | 0.723515926 | Desulfobacterota | k__Bacteria;p__Desulfobacterota;c__Desulfovibrionia;o__Desulfovibrionales;f__Desulfovibrionaceae;g__Bilophila; |
| [Eubacterium]_hallii_group | 2 | 0.621 | 0.034513172 | 0.376145294 | Firmicutes | k__Bacteria;p__Firmicutes;c__Clostridia;o__Lachnospirales;f__Lachnospiraceae;g__[Eubacterium]_hallii_group; |
| Cronobacter | 2 | 0.574 | 0.146853147 | 0.496262358 | Proteobacteria | k__Bacteria;p__Proteobacteria;c__Gammaproteobacteria;o__Enterobacterales;f__Enterobacteriaceae;g__Cronobacter; |
| UCG_003 | 2 | 0.596 | 0.471528472 | 0.867789488 | Firmicutes | k__Bacteria;p__Firmicutes;c__Clostridia;o__Oscillospirales;f__Oscillospiraceae;g__UCG-003; |
| CAG_56 | 2 | 0.598 | 0.07992008 | 0.340529036 | Firmicutes | k__Bacteria;p__Firmicutes;c__Clostridia;o__Lachnospirales;f__Lachnospiraceae;g__CAG-56; |
| Erysipelotrichaceae_UCG_003 | 2 | 0.595 | 0.291708292 | 0.723515926 | Firmicutes | k__Bacteria;p__Firmicutes;c__Bacilli;o__Erysipelotrichales;f__Erysipelatoclostridiaceae;g__Erysipelotrichaceae_UCG-003; |
| Fusicatenibacter | 2 | 0.601 | 0.786213786 | 1 | Firmicutes | k__Bacteria;p__Firmicutes;c__Clostridia;o__Lachnospirales;f__Lachnospiraceae;g__Fusicatenibacter; |
| Lachnospiraceae_FCS020_group | 2 | 0.598 | 0.538461538 | 0.879487179 | Firmicutes | k__Bacteria;p__Firmicutes;c__Clostridia;o__Lachnospirales;f__Lachnospiraceae;g__Lachnospiraceae_FCS020_group; |
| Akkermansia | 2 | 0.583 | 0.26973027 | 0.723515926 | Verrucomicrobiota | k__Bacteria;p__Verrucomicrobiota;c__Verrucomicrobiae;o__Verrucomicrobiales;f__Akkermansiaceae;g__Akkermansia; |

**Supple Table 7: Spearman's correlation analysis at the level of microbial genera in PD-MC (related to Fig 4)**

| **Genus** | **Genus** | **r** | ***p* value** |
| --- | --- | --- | --- |
| Lactobacillus | Ligilactobacillus | 0.584430602 | 6.02E-05 |
| Lactobacillus | Roseburia | -0.34580609 | 0.026788606 |
| Lactobacillus | Lachnoclostridium | -0.439832939 | 0.004008728 |
| Lactobacillus | Agathobacter | -0.366484862 | 0.018436617 |
| Lactobacillus | Limosilactobacillus | 0.777330317 | 2.27E-09 |
| Lactobacillus | Lachnospiraceae_NK4A136_group | -0.334419266 | 0.032592879 |
| Lactobacillus | [Eubacterium]_hallii_group | -0.357339774 | 0.021811028 |

| Lactobacillus | Fusicatenibacter | -0.373590837 | 0.016127954 |
| --- | --- | --- | --- |
| Lactobacillus | Erysipelotrichaceae_UCG-003 | -0.416073843 | 0.006816537 |
| Lactobacillus | Bilophila | -0.310543314 | 0.048141845 |
| Bifidobacterium | Lachnoclostridium | -0.332360246 | 0.033745531 |
| Bifidobacterium | Limosilactobacillus | 0.441042442 | 0.003897901 |
| Blautia | Roseburia | 0.401078404 | 0.009353407 |
| Blautia | Lachnoclostridium | 0.352546508 | 0.023776623 |
| Blautia | Agathobacter | 0.379851282 | 0.014301049 |
| Blautia | [Eubacterium]_hallii_group | 0.648633569 | 4.51E-06 |
| Blautia | CAG-56 | 0.366308922 | 0.01849713 |
| Blautia | Bilophila | 0.325441634 | 0.037866413 |
| Akkermansia | [Eubacterium]_hallii_group | 0.335935647 | 0.031764878 |
| Ligilactobacillus | Lachnoclostridium | -0.340609331 | 0.029321192 |
| Ligilactobacillus | Limosilactobacillus | 0.653076979 | 3.69E-06 |
| Ligilactobacillus | [Eubacterium]_hallii_group | -0.358220104 | 0.021465222 |
| Roseburia | Agathobacter | 0.728554423 | 6.67E-08 |
| Roseburia | Limosilactobacillus | -0.320486385 | 0.041063591 |
| Roseburia | Lachnospiraceae_NK4A136_group | 0.653626478 | 3.60E-06 |
| Roseburia | [Eubacterium]_hallii_group | 0.437679347 | 0.004212878 |
| Roseburia | Fusicatenibacter | 0.61857981 | 1.63E-05 |
| Roseburia | CAG-56 | 0.312689831 | 0.04653593 |
| Roseburia | Erysipelotrichaceae_UCG-003 | 0.445429909 | 0.003517992 |
| Lachnoclostridium | Agathobacter | 0.313454316 | 0.045974553 |
| Lachnoclostridium | Limosilactobacillus | -0.594380625 | 4.18E-05 |
| Lachnoclostridium | Bilophila | 0.4192208 | 0.006367186 |
| Agathobacter | Limosilactobacillus | -0.349446789 | 0.025124693 |
| Agathobacter | Lachnospiraceae_NK4A136_group | 0.672181383 | 1.49E-06 |
| Agathobacter | [Eubacterium]_hallii_group | 0.44599366 | 0.003471604 |
| Agathobacter | Fusicatenibacter | 0.68258378 | 8.84E-07 |
| Agathobacter | CAG-56 | 0.46933973 | 0.001963917 |
| Agathobacter | Erysipelotrichaceae_UCG-003 | 0.397632692 | 0.010039074 |
| Limosilactobacillus | Lachnospiraceae_NK4A136_group | -0.333392401 | 0.033163607 |
| Limosilactobacillus | CAG-56 | -0.315215219 | 0.044702322 |
| Limosilactobacillus | Bilophila | -0.51421749 | 0.000583436 |
| Lachnospiraceae_NK4A136_group | [Eubacterium]_hallii_group | 0.352712056 | 0.023706356 |
| Lachnospiraceae_NK4A136_group | Fusicatenibacter | 0.569915084 | 0.00010043 |
| Lachnospiraceae_NK4A136_group | Erysipelotrichaceae_UCG-003 | 0.391745789 | 0.011310113 |
| Lachnospiraceae_NK4A136_group | Bilophila | 0.430126621 | 0.005002145 |
| [Eubacterium]_hallii_group | Fusicatenibacter | 0.51604749 | 0.000553269 |
| [Eubacterium]_hallii_group | CAG-56 | 0.309270329 | 0.049115165 |
| [Eubacterium]_hallii_group | Erysipelotrichaceae_UCG-003 | 0.447597153 | 0.003342574 |
| Fusicatenibacter | CAG-56 | 0.422066323 | 0.005983155 |
| Fusicatenibacter | Erysipelotrichaceae_UCG-003 | 0.441168028 | 0.003886548 |

| Haemophilus | Enterobacter | 0.467922281 | 0.002035354 |
| --- | --- | --- | --- |

**Supple Table 8: Spearman's correlation analysis at the level of microbial genera in PD-NMC (related to Fig 4)**

| **Genus** | **Genus** | **r** | ***p* value** |
| --- | --- | --- | --- |
| Lactobacillus | Ligilactobacillus | 0.505604063 | 1.28E-05 |
| Lactobacillus | Limosilactobacillus | 0.525896769 | 4.87E-06 |
| Bifidobacterium | Roseburia | -0.322928848 | 0.007690543 |
| Bifidobacterium | Agathobacter | -0.335998481 | 0.005438878 |
| Bifidobacterium | CAG-56 | -0.290195374 | 0.017211499 |
| Blautia | Agathobacter | 0.330780493 | 0.006256552 |
| Blautia | Lachnospiraceae_NK4A136_group | 0.339048148 | 0.005005939 |
| Blautia | [Eubacterium]_hallii_group | 0.644622967 | 3.94E-09 |
| Blautia | Fusicatenibacter | 0.406898197 | 0.000633002 |
| Ligilactobacillus | Limosilactobacillus | 0.359882449 | 0.002778413 |
| Roseburia | Agathobacter | 0.557608892 | 9.50E-07 |
| Roseburia | Lachnospiraceae_NK4A136_group | 0.583479915 | 2.20E-07 |
| Roseburia | Fusicatenibacter | 0.501657343 | 1.53E-05 |
| Roseburia | Haemophilus | 0.385879207 | 0.001259704 |
| Roseburia | CAG-56 | 0.29015147 | 0.017229117 |
| Roseburia | Erysipelotrichaceae_UCG-003 | 0.372188946 | 0.001926027 |
| Lachnoclostridium | Limosilactobacillus | -0.267027362 | 0.02893198 |
| Lachnoclostridium | Holdemanella | -0.317074437 | 0.008939544 |
| Lachnoclostridium | Haemophilus | -0.270499807 | 0.026834342 |
| Lachnoclostridium | CAG-56 | -0.251743968 | 0.03987325 |
| Agathobacter | Limosilactobacillus | -0.275166125 | 0.024218679 |
| Agathobacter | Lachnospiraceae_NK4A136_group | 0.537426048 | 2.74E-06 |
| Agathobacter | [Eubacterium]_hallii_group | 0.4287028 | 0.000295186 |
| Agathobacter | Fusicatenibacter | 0.467913184 | 6.53E-05 |
| Agathobacter | Haemophilus | 0.284623631 | 0.019574305 |
| Agathobacter | CAG-56 | 0.493336674 | 2.22E-05 |
| Agathobacter | Erysipelotrichaceae_UCG-003 | 0.347394115 | 0.00397271 |
| Lachnospiraceae_NK4A136_group | [Eubacterium]_hallii_group | 0.344380927 | 0.004321582 |
| Lachnospiraceae_NK4A136_group | Fusicatenibacter | 0.548231567 | 1.57E-06 |
| Lachnospiraceae_NK4A136_group | CAG-56 | 0.50037658 | 1.62E-05 |
| Lachnospiraceae_NK4A136_group | Erysipelotrichaceae_UCG-003 | 0.39640495 | 0.000897523 |
| Holdemanella | Enterobacter | 0.250922271 | 0.040547435 |
| Holdemanella | Bilophila | 0.275265661 | 0.024165325 |
| [Eubacterium]_hallii_group | Fusicatenibacter | 0.521680063 | 5.98E-06 |
| [Eubacterium]_hallii_group | CAG-56 | 0.391585344 | 0.001049677 |
| [Eubacterium]_hallii_group | Erysipelotrichaceae_UCG-003 | 0.417228057 | 0.000443846 |
| Fusicatenibacter | Haemophilus | 0.323112937 | 0.007653881 |
| Fusicatenibacter | CAG-56 | 0.532478846 | 3.52E-06 |

| Fusicatenibacter | Erysipelotrichaceae_UCG-003 | 0.397394076 | 0.000868883 |
| --- | --- | --- | --- |
| Fusicatenibacter | Bilophila | 0.253230441 | 0.038677232 |
| CAG-56 | Erysipelotrichaceae_UCG-003 | 0.310727424 | 0.010490117 |

**Supple Table 9: Demographic characteristics of the metabolomics cohort 2 and cohort 3**

| **Characteristics** | **Cohort 2 for Metabolome** | | | **Cohort 3 for Metabolome** | | |
| --- | --- | --- | --- | --- | --- | --- |
|  | **PD-MC** | **PD-NMC** | ***p* value** | **PD-MC** | **PD-NMC** | ***p* value** |
|  | **（N=50）** | **（N=90）** |  | **（N=37）** | **（N=69）** |  |
| Age, years | 66.4± 7.3 | 64.5± 9.2 | 0.211 | 69.8± 8.6 | 66.2± 13.2 | 0.138 |
| Females, n (%) | 26 (52%) | 36 (40%) | 0.171 | 20 (54.1%) | 35 (50.7%) | 0.744 |
| BMI, kg/m** | 23.4± 4.4 | 24.1± 3.0 | 0.189 | 24.6± 4.1 | 23.4± 3.0 | 0.132 |
| Education, years | 2 (0, 6) | 5 (1, 8) | 0.020 | 3 (0, 6) | 5 (0, 6) | 0.237 |
| Disease duration, years | 7 (5, 10) | 2 (1, 4) | <0.001 | 8 (4, 12) | 2 (1, 2.5) | <0.001 |
| Cigarette, n (%) | 10 (20%) | 20 (22.2%) | 0.759 | 5 (13.5%) | 13 (18.8%) | 0.486 |
| Alcohol, n (%) | 9 (18%) | 29 (32.2%) | 0.064 | 5 (13.5%) | 19 (27.5%) | 0.100 |
| Hypertension, n (%) | 15 (30%) | 27(30%) | 1.000 | 14 (37.8%) | 27 (39.1%) | 0.896 |
| Diabetes, n (%) | 7 (14%) | 15 (16.7%) | 0.678 | 8 (21.6%) | 10 (14.5%) | 0.351 |
| Constipation, n (%) | 36 (72%) | 28 (31.1%) | <0.001 | 29 (78.4%) | 28 (40.6%) | <0.001 |
| Dyskinesia, n (%) | 12 (24%) | 0 (0%) | <0.001 | 8 (21.6%) | 0 (0%) | <0.001 |
| Fall, n (%) | 15 (30%) | 10 (11.2%) | 0.006 | 17 (45.9%) | 11 (15.9%) | <0.001 |
| UPDRS total score | 60 (48, 77) | 33 (21, 46) | <0.001 | 53 (35.5, 75.5) | 37 (22.5, 53.5) | 0.003 |
| UPDRS part I score | 3 (1, 6) | 1.5 (0.75, 3) | 0.004 | 2 (1, 3) | 2 (1, 3) | 0.758 |
| UPDRS part II score | 17.5 (13.75, 23) | 9 (5, 13) | <0.001 | 15 (9, 21.5) | 11 (6, 14) | 0.001 |
| UPDRS part III score | 35.5 (24.75, 43) | 22 (13, 30.25) | <0.001 | 33 (21, 46) | 24 (14, 37.5) | 0.023 |
| UPDRS part IV score | 5.5 (4, 8) | 1 (0, 2) | <0.001 | 3 (1, 6) | 0 (0, 1) | <0.001 |
| H-Y stage | 3 (2.9, 4) | 2 (1.5, 3) | <0.001 | 3 (2, 4) | 2 (2, 3) | 0.012 |
| MMSE | 18.9± 7.3 | 22.7± 6.1 | 0.002 | 18.9± 6.5 | 19.8± 6.6 | 0.477 |
| HAMA | 12 (6, 14.3) | 7.5 (4, 11) | <0.001 | 7 (3.3, 10) | 8 (3, 11.5) | 0.614 |
| HAMD | 9 (4, 11) | 4 (2, 7) | <0.001 | 5 (3, 8.5) | 5 (2, 8.5) | 0.730 |
| RBDQ-HK | 27.5 (12.8, 44.3) | 9 (3, 22) | <0.001 | 14 (3.3, 28.75) | 10 (2, 28.5) | 0.634 |
| ADL | 35 (28, 45) | 22.5 (20, 28) | <0.001 | 35 (29.3, 53) | 28 (21, 36) | <0.001 |
| PD medication, n (%) | 50 (100%) | 80 (88.9%) | <0.001 | 37 (100%) | 53 (76.9%) | <0.001 |
| L-DOPA intake | 46 (92%) | 79 (87.8%) | 0.439 | 36 (97.3%) | 50 (72.5%） | 0.002 |
| Dopamine agonists | 42(84%) | 54 (60%) | 0.003 | 27 (73%) | 30 (43.5%) | 0.004 |
| MAO-B inhibitor | 11 (22%) | 18 (20%) | 0.780 | 6 (16.2%) | 10 (14.5%) | 0.813 |
| COMT inhibitor | 23 (46%) | 9 (10%) | <0.001 | 17 (45.9%) | 10 (14.5%) | <0.001 |
| Amantadine | 11 (22%) | 19 (21.1%) | 0.902 | 11 (29.7%) | 5 (7.2%) | 0.002 |
| Benzhexol hydrochloride | 3 (6%) | 4 (4.4%) | 0.686 | 2 (5.4%) | 2 (2.9%) | 0.519 |
| LED mg/day | 575 (387.5, 801.9) | 375 (187.5, 456.3) | <0.001 | 637.5 (405.75, 731.3) | 300 (25, 400) | <0.001 |

**Supple Table 10: Result of Histograms and volcano charts in cohort 2 (related to Fig 5i, k)**

| **Metabolite encode** | **Fold change (FC)** | **log2FC** | ***p* value** | **expression** | **VIP** | **Ranked by mean decrease accuracy** | **Ranked by mean decrease gini** | **Metabolite identification** |
| --- | --- | --- | --- | --- | --- | --- | --- | --- |
| 919 | 6.4699 | 2.6937 | 0.0400 | up | 9.3096 | 1239 | 2892 | undefined |
| 719 | 20.6557 | 4.3685 | 0.0266 | up | 7.1614 | 5297 | 1750 | undefined |
| 728 | 16.0282 | 4.0025 | 0.0240 | up | 6.6465 | 4909 | 1652 | undefined |
| 937 | 4.7669 | 2.2530 | 0.0441 | up | 5.644 | 1412 | 2195 | undefined |
| 1730 | 0.3027 | -1.7238 | 0.0447 | down | 5.5036 | 3077 | 3996 | Pramipexole |
| 1143 | 2.5798 | 1.3672 | 0.0215 | up | 4.5096 | 1534 | 425 | undefined |
| 3294 | 4.3550 | 2.1227 | 0.0110 | up | 4.353 | 1 | 5 | 3-deoxysappanchalcone |
| 717 | 3.7460 | 1.9054 | 0.0053 | up | 4.0225 | 13 | 8 | 1,3-Dimethyluracil |
| 1072 | 3.9669 | 1.9880 | 0.0186 | up | 3.7884 | 550 | 885 | undefined |
| 47 | 4.9718 | 2.3138 | 0.0317 | up | 3.7006 | 1208 | 2787 | undefined |
| 46 | 5.1910 | 2.3760 | 0.0295 | up | 3.5864 | 2388 | 3307 | undefined |
| 2928 | 3.2539 | 1.7022 | 0.0108 | up | 3.5854 | 3597 | 4516 | undefined |
| 106 | 5.2862 | 2.4022 | 0.0250 | up | 3.5498 | 1187 | 2689 | undefined |
| 2716 | 2.8225 | 1.4970 | 0.0019 | up | 3.4325 | 359 | 107 | Histidylmethionine |
| 1145 | 2.3854 | 1.2542 | 0.0356 | up | 3.3648 | 1532 | 249 | undefined |
| 1254 | 2.7357 | 1.4519 | 0.0240 | up | 3.1462 | 2893 | 3812 | Valylproline |
| 1742 | 0.4731 | -1.0797 | 0.0419 | down | 3.1429 | 1616 | 944 | Prolylproline |
| 419 | 2.3469 | 1.2308 | 0.0240 | up | 3.1275 | 5711 | 727 | L-Aspartate-semialdehyde |
| 2008 | 0.1634 | -2.6139 | 0.0126 | down | 2.7239 | 3208 | 4127 | Droxidopa |
| 3267 | 3.1377 | 1.6497 | 0.0019 | up | 2.6016 | 12 | 68 | Epinephrine glucuronide |
| 3979 | 2.2760 | 1.1865 | 0.0045 | up | 2.4239 | 184 | 334 | undefined |
| 3134 | 2.6628 | 1.4130 | 0.0000 | up | 2.2258 | 170 | 31 | undefined |
| 915 | 2.0834 | 1.0590 | 0.0069 | up | 2.1571 | 2758 | 3677 | Methyl 2-octynoate |
| 253 | 2.0682 | 1.0484 | 0.0008 | up | 2.1133 | 1451 | 76 | Betaine-Aldehyde |
| 3229 | 2.3664 | 1.2427 | 0.0001 | up | 2.0944 | 4 | 36 | undefined |
| 3117 | 2.1652 | 1.1145 | 0.0001 | up | 2.0631 | 5023 | 470 | 18-methylnonadecanoic acid |
| 5455 | 2.1739 | 1.1203 | 0.0006 | up | 2.0474 | 332 | 232 | 9-Hydroxytridecyl docosanoate |
| 1163 | 2.1785 | 1.1233 | 0.0008 | up | 2.0466 | 22 | 172 | N-Acetylisoleucine |
| 5464 | 2.1537 | 1.1068 | 0.0006 | up | 2.0103 | 1600 | 817 | undefined |
| 5469 | 2.2282 | 1.1559 | 0.0006 | up | 1.9805 | 1595 | 762 | Cer(d18:1/18:1(11Z)) |
| 1142 | 2.0320 | 1.0229 | 0.0049 | up | 1.9558 | 1685 | 1398 | undefined |
| 5476 | 2.1963 | 1.1351 | 0.0006 | up | 1.9368 | 266 | 962 | undefined |
| 1382 | 2.0656 | 1.0466 | 0.0037 | up | 1.8881 | 5504 | 2542 | undefined |
| 3687 | 0.4964 | -1.0105 | 0.0093 | down | 1.8495 | 1670 | 1295 | undefined |
| 581 | 2.9123 | 1.5422 | 0.0003 | up | 1.727 | 272 | 10 | Leucine |
| 2949 | 2.3286 | 1.2195 | 0.0043 | up | 1.7034 | 10 | 56 | Tyrosyl-Threonine |
| 2088 | 0.4247 | -1.2356 | 0.0096 | down | 1.6294 | 370 | 216 | undefined |
| 357 | 3.0191 | 1.5941 | 0.0104 | up | 1.6105 | 5130 | 1057 | Histamine |
| 4675 | 0.4955 | -1.0132 | 0.0423 | down | 1.5528 | 1730 | 1641 | LysoPC(14:0/0:0) |
| 1210 | 0.4563 | -1.1319 | 0.0401 | down | 1.1003 | 5166 | 1222 | Ascorbic acid |

**Supple Table 11: result of Histograms and volcano charts in cohort 3（ related to Fig 5l, j）**

| **Metabolite encode** | **Fold change (FC)** | **log2FC** | ***p* value** | **expression** | **VIP** | **Ranked by mean decrease accuracy** | **Ranked by mean decrease gini** | **Metabolite identification** |
| --- | --- | --- | --- | --- | --- | --- | --- | --- |
| 1782 | 2.5790 | 1.3668 | 7.41015E-08 | up | 11.764 | 1902 | 2022 | Docosenic acid |
| 1983 | 2.3587 | 1.2380 | 1.2918E-24 | up | 7.8549 | 372 | 153 | 25-Azacholesterol |
| 2091 | 9.2471 | 3.2090 | 4.47097E-08 | up | 6.293 | 5 | 10 | dodec-6-enoic acid |
| 1000 | 0.4496 | -1.1534 | 4.67211E-13 | down | 5.8963 | 1268 | 1427 | undefined |
| 1981 | 2.4352 | 1.2840 | 3.52411E-25 | up | 5.2299 | 89 | 63 | undefined |
| 1813 | 2.5886 | 1.3722 | 2.70241E-08 | up | 5.1085 | 1926 | 2044 | Deoxycytidine monophosphate (dCMP) |
| 1376 | 0.4671 | -1.0982 | 2.66791E-06 | down | 3.7242 | 1577 | 1716 | undefined |
| 1477 | 2.1122 | 1.0787 | 2.14387E-12 | up | 3.3656 | 1662 | 1795 | Octadecenoic acid |
| 502 | 23.6776 | 4.5655 | 0.041494366 | up | 3.3537 | 856 | 1034 | 4-Trimethylammoniobutanoic acid |
| 1002 | 0.4364 | -1.1964 | 2.30554E-12 | down | 2.9735 | 1270 | 610 | Glutamylproline |
| 1785 | 2.6990 | 1.4324 | 1.0497E-08 | up | 2.8715 | 2900 | 294 | undefined |
| 2607 | 0.4546 | -1.1372 | 8.60966E-07 | down | 2.8034 | 101 | 213 | Bilirubin |
| 127 | 0.4829 | -1.0501 | 1.35763E-07 | down | 2.6413 | 542 | 739 | p-Cresol |
| 1781 | 2.7734 | 1.4717 | 1.70356E-24 | up | 2.5718 | 83 | 69 | N-butyl Oleate |
| 936 | 33.6548 | 5.0727 | 1.33203E-22 | up | 2.0758 | 66 | 57 | 4-hydroxyundecanoic acid |
| 1812 | 2.5821 | 1.3686 | 1.25393E-10 | up | 1.6591 | 115 | 145 | undefined |
| 1989 | 2.3884 | 1.2560 | 3.36988E-25 | up | 1.6367 | 2058 | 2169 | undefined |
| 1289 | 0.4166 | -1.2633 | 1.8011E-06 | down | 1.4339 | 1507 | 1648 | undefined |
| 1681 | 2.0124 | 1.0089 | 1.76637E-15 | up | 1.2041 | 1834 | 1958 | Nicotinamide ribotide |
| 409 | 0.4636 | -1.1092 | 2.26014E-12 | down | 1.1929 | 778 | 963 | undefined |
| 827 | 0.2307 | -2.1160 | 1.61185E-16 | down | 1.0874 | 1127 | 1293 | 7-methyldecanoic acid |
| 184 | 0.4852 | -1.0434 | 2.43266E-14 | down | 1.0627 | 586 | 781 | undefined |
| 875 | 3.4427 | 1.7836 | 1.53595E-25 | up | 1.011 | 84 | 172 | undefined |

**Supple Table 12: Random Forest top15 metabolites in cohort2 (related to Fig 6c)**

| **Ranked by mean decrease accuracy top 15** | | | **Ranked by mean decrease gini top 15** | | |
| --- | --- | --- | --- | --- | --- |
| **Metabolite encode** | **mean decrease accuracy** | **Metabolite identification** | **Metabolite encode** | **mean decrease gini** | **Metabolite identification** |
| 3294 | 3.0014 | 3-deoxysappanchalcone | 1369 | 0.2888 | 4-Guanidinobutanoic acid |
| 2583 | 2.8123 | undefined | 2583 | 0.2687 | undefined |
| 1369 | 2.6203 | undefined | 1386 | 0.2680 | undefined |
| 3229 | 2.5540 | undefined | 567 | 0.2629 | Cytidine |
| 2546 | 2.4470 | undefined | 3294 | 0.2486 | 3-deoxysappanchalcone |
| 4103 | 2.3449 | undefined | 4103 | 0.2451 | undefined |
| 935 | 2.3303 | undefined | 4182 | 0.1909 | undefined |
| 3228 | 2.2680 | undefined | 717 | 0.1790 | 1,3-Dimethyluracil |
| 2251 | 2.2581 | Daidzein | 4778 | 0.1723 | undefined |
| 2949 | 2.1914 | Tyrosyl-Threonine | 581 | 0.1672 | Leucine |
| 2576 | 2.1762 | Sulfamethazine | 2670 | 0.1604 | undefined |
| 3267 | 2.1555 | Epinephrine glucuronide | 935 | 0.1604 | undefined |

| 717 | 2.1272 | 1,3-Dimethyluracil | 1385 | 0.1579 | undefined |
| --- | --- | --- | --- | --- | --- |
| 2596 | 2.1154 | linolenic acid | 5693 | 0.1470 | undefined |
| 1154 | 2.0886 | undefined | 1154 | 0.1428 | undefined |

**Supple Table 13: Random Forest top15 metabolites in cohort3 (related to Fig 6d)**

| **Ranked by mean decrease accuracy top 15** | | | **Ranked by mean decrease gini top 15** | | |
| --- | --- | --- | --- | --- | --- |
| **Metabolite encode** | **mean decrease accuracy** | **Metabolite identification** | **Metabolite encode** | **mean decrease gini** | **Metabolite identification** |
| 2013 | 4.0007 | Chenodiol | 2013 | 1.1576 | Chenodiol |
| 754 | 3.8950 | undefined | 754 | 1.1317 | undefined |
| 2067 | 3.8811 | 2-acetoxy-4-pentadecylbenzoic acid | 1792 | 1.0692 | undefined |
| 1151 | 3.7904 | undefined | 2215 | 1.0539 | undefined |
| 2091 | 3.7582 | dodec-6-enoic acid | 1151 | 0.9857 | undefined |
| 2215 | 3.7548 | undefined | 883 | 0.9839 | undefined |
| 1792 | 3.6445 | undefined | 1715 | 0.9686 | undefined |
| 883 | 3.4078 | undefined | 61 | 0.8956 | Pyridine |
| 1715 | 3.3898 | undefined | 961 | 0.8769 | L-Tryptophan |
| 61 | 3.3172 | Pyridine | 2091 | 0.8613 | dodec-6-enoic acid |
| 1961 | 3.2678 | undefined | 327 | 0.8591 | undefined |
| 1918 | 3.2429 | undefined | 1742 | 0.8075 | undefined |
| 327 | 3.2238 | undefined | 2067 | 0.8002 | 2-acetoxy-4-pentadecylbenzoic acid |
| 1737 | 3.2082 | undefined | 1918 | 0.7962 | undefined |
| 1269 | 3.2020 | undefined | 1737 | 0.7492 | undefined |

**Supple Table14: KEGG pathway enrichment (related to Fig 8a)**

| **KEGG pathway** | **Pathway impact** | | ***p* value** | **Count** | **Hit** | **Metabolites（ KEGG encode）** | **Expression** | **Classification** |
| --- | --- | --- | --- | --- | --- | --- | --- | --- |
| Biosynthesis of unsaturated fatty acids | 0 |  | 0.033832 | 36 | 2 | Octadecenoic acid (C00712) | up | lipid |
| Valine, leucine and isoleucine biosynthesis | 0 |  | 0.064294 | 8 | 1 | Leucine (C00123) | up | lipid |
| alpha-Linolenic acid metabolism | 0.33333 |  | 0.10252 | 13 | 1 | Octadecenoic acid (C00712) | up | Amino acids |
| Nicotinate and nicotinamide metabolism | 0.03158 |  | 0.1174 | 15 | 1 | Nicotinamide ribotide (C00455) | up | Cofactor/vitamin |
| Histidine metabolism | 0.18852 |  | 0.12475 | 16 | 1 | Histamine (C00388) | up | Amino acids |
| Lysine degradation | 0.00204 |  | 0.22196 | 30 | 1 | 4-Trimethylammoniobutanoic acid (C01181) | up | Amino acids |
| Porphyrin metabolism | 0.0528 |  | 0.2285 | 31 | 1 | Bilirubin (C0486) | Down | Cofactor/vitamin |
| Sphingolipid metabolism | 0.21576 |  | 0.235 | 32 | 1 | Cer(d18:1/18:1(11Z)) (C00195) | Down | lipid |
| Glycine, serine and threonine metabolism | 0.02673 |  | 0.24145 | 33 | 1 | Betaine-Aldehyde (C00576) | up | Amino acids |
| Arginine and proline metabolism | 0 |  | 0.26048 | 36 | 1 | 4-Guanidinobutanoic acid (C01035) | up | Amino acids |
| Glycerophospholipid metabolism | 0.01736 |  | 0.26048 | 36 | 1 | LysoPC(14:0/0:0) (C04230) | Down | lipid |
| Valine, leucine and isoleucine degradation | 0 |  | 0.28518 | 40 | 1 | Leucine (C00123) | up | Amino acids |
| Tryptophan metabolism | 0.14305 |  | 0.29123 | 41 | 1 | Chenodiol (C00078) | up | Amino acids |
| Primary bile acid biosynthesis | 0 |  | 0.3208 | 46 | 1 | L-Tryptophan (C02528) | up | lipid |

**Supple Table 15: SPMBD pathway enrichment（ related to Fig 8b, c, d）**

| **SPMBD pathway** | **Enrichment ratio** | ***p* value** | **Count** | **Hit** | **Metabolites（ KEGG encode）** | **ID** | **classification** |
| --- | --- | --- | --- | --- | --- | --- | --- |
| Carnitine Synthesis | 7.604562738 | 0.0266 | 22 | 2 | Ascorbic acid (C00072); 4-Trimethylammoniobutanoic acid (C01181) | SMP0000465 | Lipid |
| Oxidation of Branched Chain Fatty Acids | 6.430868167 | 0.0364 | 26 | 2 | Leucine (C00123); Phytanic acid (C01607) | SMP0000030 | Lipid |
| Phytanic Acid Peroxisomal Oxidation | 6.430868167 | 0.0364 | 26 | 2 | Leucine (C00123); Phytanic acid (C01607) | SMP0000450 | Lipid |
| Alpha Linolenic Acid and Linoleic Acid Metabolism | 4.901960784 | 0.187 | 17 | 1 | alpha-Linolenic acid (C06427) | SMP0087171 | Lipid |
| Catecholamine Biosynthesis | 4.166666667 | 0.216 | 20 | 1 | Ascorbic acid (C00072) | SMP0000012 | Amino acid |
| Betaine Metabolism | 3.984063745 | 0.226 | 21 | 1 | Betaine aldehyde (C00576) | SMP0063600 | Amino acid |
| Nicotinate and Nicotinamide Metabolism | 2.386634845 | 0.349 | 35 | 1 | Nicotinamide ribotide (C00455) | SMP0000048 | Cofactor/vitami  n |
| Fatty Acid Biosynthesis | 2.386634845 | 0.349 | 35 | 1 | trans-Dodec-2-enoic acid (C21202) | SMP0000456 | Lipid |
| Porphyrin Metabolism | 2.087682672 | 0.388 | 40 | 1 | Bilirubin (C0486) | SMP0000024 | Cofactor/vitami  n |
| Histidine Metabolism | 1.988071571 | 0.404 | 42 | 1 | Histamine (C00388) | SMP0063632 | Amino acid |
| Valine, Leucine and Isoleucine Degradation | 1.414427157 | 0.519 | 59 | 1 | Leucine (C00123) | SMP0000032 | Amino acid |
| Tryptophan Metabolism | 1.414427157 | 0.519 | 59 | 1 | 4-Guanidinobutanoic acid (C01035) | SMP0000063 | Amino acid |
| Bile Acid Biosynthesis | 1.285347044 | 0.555 | 65 | 1 | Chenodiol (C00078) | SMP0000035 | Lipid |
| Tyrosine Metabolism | 1.193317422 | 0.583 | 70 | 1 | L-Tryptophan (C02528) | SMP0000006 | Amino acid |

**Supple Table 16: Spearman's correlation analysis at the level of microbial genera and Metabolite in PD-MC individuals (related to Fig 9a-c)**

| **microbial genera** | **Metabolite** | **r** | ***p* value** |
| --- | --- | --- | --- |
| Agathobacter | Pramipexole | -0.811 | 0.027 |
| Agathobacter | Tyrosyl-Threonine | -0.811 | 0.027 |
| Blautia | 4-Trimethylammoniobutanoicacid | 0.673 | 0.033 |
| Ligilactobacillus | Pramipexole | -0.867 | 0.012 |
| Ligilactobacillus | Tyrosyl-Threonine | -0.867 | 0.012 |
| Ligilactobacillus | dodec-6-enoicacid | 0.705 | 0.023 |
| Ligilactobacillus | Octadecenoicacid | 0.644 | 0.044 |
| Ligilactobacillus | pCresol | -0.669 | 0.035 |
| Roseburia | Leucine | -0.811 | 0.027 |
| Roseburia | N-Acetylisoleucine | -0.811 | 0.027 |
| Limosilactobacillus | Ascorbic acid | -0.775 | 0.041 |
| Lachnoclostridium | Glutamylproline | 0.758 | 0.011 |
| Enterobacter | Docosenicacid | -0.644 | 0.044 |
| Enterobacter | Ascorbic acid | -0.873 | 0.010 |
| Haemophilus | Cer(d18:1/18:1(11Z)) | 0.757 | 0.049 |
| Halomonas | 18-methylnonadecanoic acid | 0.823 | 0.023 |
| Eubacterium_hallii_group | NbutylOleate | 0.632 | 0.050 |

| Eubacterium_hallii_group | 25-Azacholesterol | 0.656 | 0.039 |
| --- | --- | --- | --- |
| Eubacterium_hallii_group | Leucine | 0.786 | 0.036 |
| Eubacterium_hallii_group | N-Acetylisoleucine | 0.786 | 0.036 |
| Bilophila | Ascorbic acid | 0.821 | 0.023 |
| Cronobacter | Ascorbic acid | -0.867 | 0.012 |
| UCG_003 | Tyrosyl-Threonine | -0.811 | 0.027 |
| UCG_003 | NbutylOleate | 0.886 | 0.001 |
| UCG_003 | 4-hydroxyundecanoicacid | 0.795 | 0.006 |
| UCG_003 | 25-Azacholesterol | 0.899 | 0.000 |
| UCG_003 | Bilirubin | 0.705 | 0.023 |
| UCG_003 | pCresol | -0.666 | 0.036 |
| UCG_003 | Nicotinamideribotide | 0.653 | 0.041 |
| CAG_56 | 4-Trimethylammoniobutanoicacid | 0.884 | 0.001 |
| CAG_56 | Leucine | -0.867 | 0.012 |
| CAG_56 | 1,3-Dimethyluracil | -0.788 | 0.035 |
| CAG_56 | N-Acetylisoleucine | -0.867 | 0.012 |
| CAG_56 | Pramipexole | -0.808 | 0.028 |
| Erysipelotrichaceae_UCG_003 | Histidylmethionine | -0.847 | 0.016 |
| Fusicatenibacter | 1,3-Dimethyluracil | -0.883 | 0.008 |
| Akkermansia | Cer(d18:1/18:1(11Z)) | 0.852 | 0.015 |
| Akkermansia | dodec6enoicacid | 0.690 | 0.027 |
| Akkermansia | NbutylOleate | 0.744 | 0.014 |
| Akkermansia | Docosenicacid | 0.676 | 0.032 |
| Akkermansia | 25-Azacholesterol | 0.731 | 0.016 |
| Akkermansia | Octadecenoicacid | 0.655 | 0.040 |
| Akkermansia | pCresol | -0.751 | 0.012 |
| Akkermansia | Nicotinamideribotide | 0.799 | 0.006 |

**Supple Table 17: Spearman's correlation analysis at the level of microbial genera and Metabolite in all PD individuals (related to Supple Fig. 3)**

| **microbial genera** | **Metabolite** | **r** | ***p* value** |
| --- | --- | --- | --- |
| Agathobacter | N-Acetylisoleucine | -0.537 | 0.032 |
| Agathobacter | Prolylproline | 0.531 | 0.034 |
| Agathobacter | dodec6enoic acid | -0.553 | 0.009 |
| Agathobacter | DeoxycytidinemonophosphatedCMP | -0.478 | 0.028 |
| Agathobacter | pCresol | 0.446 | 0.043 |
| Lactobacillus | dodec6enoic acid | 0.627 | 0.002 |
| Lactobacillus | N-butylOleate | 0.595 | 0.004 |
| Lactobacillus | Docosenic acid | 0.435 | 0.049 |
| Lactobacillus | 25Azacholesterol | 0.488 | 0.025 |
| Lactobacillus | Octadecenoic acid | 0.456 | 0.038 |
| Lactobacillus | pCresol | -0.499 | 0.021 |

| Lactobacillus | Nicotinamideribotide | 0.611 | 0.003 |
| --- | --- | --- | --- |
| Blautia | Blautia | -0.556 | 0.025 |
| Lachnospiraceae_NK4A136_group | Cer(d18:1/18:1(11Z)) | 0.595 | 0.015 |
| Lachnospiraceae_NK4A136_group | Ascorbic acid | 0.610 | 0.012 |
| Ligilactobacillus | Leucine | -0.536 | 0.032 |
| Ligilactobacillus | N-Acetylisoleucine | -0.545 | 0.029 |
| Ligilactobacillus | dodec6enoic acid | 0.701 | 0.000 |
| Ligilactobacillus | N-butylOleate | 0.501 | 0.021 |
| Ligilactobacillus | 4-hydroxyundecanoic acid | 0.447 | 0.042 |
| Ligilactobacillus | Octadecenoic acid | 0.482 | 0.027 |
| Ligilactobacillus | pCresol | -0.726 | 0.000 |
| Ligilactobacillus | Nicotinamideribotide | 0.496 | 0.022 |
| Bacillus | dodec6enoic acid | 0.638 | 0.002 |
| Bacillus | N-butylOleate | 0.461 | 0.036 |
| Bacillus | Octadecenoic acid | 0.435 | 0.049 |
| Bacillus | pCresol | -0.471 | 0.031 |
| Limosilactobacillus | dodec6enoic acid | 0.712 | 0.000 |
| Limosilactobacillus | N-butylOleate | 0.693 | 0.001 |
| Limosilactobacillus | Docosenic acid | 0.591 | 0.005 |
| Limosilactobacillus | 25Azacholesterol | 0.580 | 0.006 |
| Limosilactobacillus | Octadecenoic acid | 0.679 | 0.001 |
| Limosilactobacillus | Glutamylproline | -0.487 | 0.025 |
| Limosilactobacillus | pCresol | -0.703 | 0.000 |
| Limosilactobacillus | Nicotinamideribotide | 0.676 | 0.001 |
| Limosilactobacillus | 7methyldecanoic acid | -0.437 | 0.048 |
| Bifidobacterium | Octadecenoic acid | 0.538 | 0.012 |
| Bifidobacterium | 1,3-Dimethyluracil | 0.668 | 0.005 |
| Lachnoclostridium | Glutamylproline | 0.469 | 0.032 |
| Lachnoclostridium | 7-methyldecanoic acid | 0.503 | 0.020 |
| Holdemanella | Bilirubin | 0.504 | 0.020 |
| Cryptobacterium | Prolylproline | -0.499 | 0.049 |
| Halomonas | 3-deoxysappanchalcone | 0.584 | 0.018 |
| Halomonas | LysoPC(14:0/0:0) | -0.590 | 0.016 |
| Eubacterium_hallii_group | N-Acetylisoleucine | -0.521 | 0.039 |
| Eubacterium_hallii_group | Bilirubin | 0.460 | 0.036 |
| Bilophila | N-butylOleate | 0.449 | 0.041 |
| Bilophila | 25-Azacholesterol | 0.443 | 0.309 |
| Bilophila | Nicotinamideribotide | 0.531 | 0.013 |
| Halomonas | dodec6enoicacid | 0.518 | 0.016 |
| Halomonas | Octadecenoic acid | 0.530 | 0.013 |
| Halomonas | pCresol | -0.482 | 0.027 |
| Halomonas | Nicotinamideribotide | 0.450 | 0.041 |
| Aliidiomarina | dodec6enoic acid | 0.436 | 0.048 |

| Aliidiomarina | pCresol | -0.470 | 0.032 |
| --- | --- | --- | --- |
| Chelativorans | dodec6enoic acid | 0.457 | 0.037 |
| Chelativorans | pCresol | -0.466 | 0.033 |
| Chelativorans | Nicotinamideribotide | 0.468 | 0.032 |
| Cronobacter | 4-hydroxyundecanoicacid | 0.493 | 0.023 |
| Cronobacter | 25-Azacholesterol | 0.452 | 0.040 |
| Cronobacter | Ascorbic acid | -0.509 | 0.044 |
| CAG_56 | Leucine | -0.660 | 0.005 |
| CAG_56 | N-Acetylisoleucine | -0.609 | 0.012 |
| Fusicatenibacter | dodec6enoic acid | -0.509 | 0.018 |
| Akkermansia | Leucine | 0.539 | 0.031 |
| Akkermansia | dodec6enoic acid | 0.454 | 0.039 |
| Akkermansia | N-butylOleate | 0.583 | 0.006 |
| Akkermansia | 25-Azacholesterol | 0.624 | 0.003 |
| Akkermansia | Octadecenoic acid | 0.702 | 0.000 |
| Akkermansia | pCresol | -0.602 | 0.004 |
| Akkermansia | Nicotinamideribotide | 0.581 | 0.006 |
| Erysipelotrichaceae_UCG_003 | pCresol | 0.499 | 0.021 |
| UCG_003 | 25-Azacholesterol | 0.533 | 0.013 |

**Supple table 18: Subgroup analysis of characteristic microorganisms and metabolites of PD-MC**

| **Classification** | **Dyskinesia** | **Symptom fluctuation** | **Combined group** | ***p* value** |
| --- | --- | --- | --- | --- |
| Number | 5 | 27 | 10 |  |
| **Microbiota in Cohort 1** | | | | |
| Lactobacillus | 2.2E-04 (2.2E-04) | 2.2E-03 (1.7E-02) | 1.2E-04 (3.0E-04) | 0.672 |
| Agathobacter | 1.8E-03 (1.7E-03) | 7.7E-03 (1.2E-02) | 8.3E-03 (1.5E-02) | 0.594 |
| Prevotella_7 | 0.0E-00 (0.0E-00) | 1.7E-03 (9.0E-03) | 0.0E-00 (0.0E-00) | 0.766 |
| Blautia | 0.02 (0.01) | 0.01 (0.03) | 0.01 (0.01) | 0.938 |
| Lachnospiraceae_NK4A136_group | 1.9E-04 (2.4E-04) | 4.5E-03 (8.1E-03) | 9.0E-04 (1.4E-03) | 0.205 |
| Bacillus | 9.7E-06 (1.3E-05) | 2.8E-05 (4.6E-05) | 2.9E-05 (3.9E-05) | 0.656 |
| Ligilactobacillus | 8.8E-04 (1.5E-03) | 2.6E-03 (5.2E-03) | 3.0E-02 (9.2E-02) | 0.252 |
| Roseburia | 8.5E-03 (1.5E-02) | 7.7E-03 (1.5E-02) | 4.0E-03 (5.9E-03) | 0.745 |
| Limosilactobacillus | 4.1E-02 (9.1E-02) b, c | 1.5E-03 (5.8E-03) a, c | 7.1E-04 (1.5E-03) a, b | 0.028 |
| Bifidobacterium | 2.0E-02 (4.0E-04) | 2.7E-02 (5.7E-02) | 2.3E-02 (4.5E-02) | 0.944 |
| Lachnoclostridium | 7.8E-03 (8.2E-03) | 8.8E-03 (9.4E-03) | 1.2E-02 (1.2E-02) | 0.652 |
| Holdemanella | 6.0E-04 (1.3E-03) | 9.7E-04 (2.2E-03) | 7.4E-04 (1.9E-03) | 0.548 |
| Enterobacter | 0.0E-00 (0.0E-00) | 3.1E-03 (7.7E-03) | 5.1E-05 (9.2E-05) | 0.323 |
| Epulopiscium | 0.0E-00 (0.0E-00) | 9.1E-06 (4.2E-05) | 0.0E-00 (0.0E-00) | 0.721 |
| Dietzia | 4.9E-06 (1.1E-05) | 8.1E-06 (1.9E-05) | 3.2E-05 (1.9E-05) | 0.206 |
| Alcanivorax | 0.0E-00 (0.0E-00) | 2.7E-05 (4.4E-05) | 3.4E-05 (4.2E-05) | 0.312 |
| Cryptobacterium | 0.0E-00 (0.0E-00) | 9.0E-06 (2.0E-05) | 0.0E-00 (0.0E-00) | 0.256 |
| Chelativorans | 4.8E-06 (1.1E-05) | 2.3E-05 (3.1E-05) | 1.4E-05 (2.1E-05) | 0.370 |
| Azoarcus | 4.9E-06 (1.1E-05) | 4.5E-06 (1.4E-05) | 4.9E-06 (1.5E-05) | 0.997 |
| Haemophilus | 7.8E-05 (1.4E-04) | 1.0E-03 (4.4E-03) | 6.6E-04 (1.9E-03) | 0.860 |
| Aliidiomarina | 5.8E-05 (4.4E-05) | 5.4E-05 (7.9E-05) | 5.4E-05 (7.1E-05) | 0.992 |
| Halomonas | 1.6E-04 (1.2E-04) | 3.2E-04 (3.9E-04) | 9.8E-04 (2.1E-03) | 0.210 |
| Bilophila | 4.2E-03 (7.8E-03) | 2.5E-03 (3.4E-03) | 3.0E-03 (4.0E-03) | 0.692 |
| Eubacterium_hallii_group | 2.9E-03 (5.2E-03) | 2.1E-03 (5.0E-03) | 1.5E-03 (2.0E-03) | 0.838 |
| Cronobacter | 0.0E-00 (0.0E-00) | 2.2E-05 (5.6E-05) | 1.5E-05 (4.6E-05) | 0.670 |
| UCG_003 | 1.6E-03 (2.6E-03) | 1.6E-03 (3.2E-03) | 1.5E-03 (2.3E-03) | 0.995 |
| CAG_56 | 4.0E-04 (2.6E-04) | 5.7E-04 (1.0E-03) | 4.1E-04 (6.6E-04) | 0.853 |
| Erysipelotrichaceae_UCG_003 | 2.8E-04 (4.0E-04) | 1.6E-03 (3.2E-03) | 1.9E-03 (3.0E-03) | 0.584 |
| Fusicatenibacter | 2.5E-04 (2.4E-04) | 3.5E-03 (7.8E-03) | 1.9E-03 (1.8E-03) | 0.539 |
| Lachnospiraceae_FCS020_group | 1.1E-04 (1.4E-04) | 1.1E-04 (1.6E-04) | 1.8 E-04 (2.6E-04) | 0.536 |
| Akkermansia | 2.1E-02 (4.6E-02) | 7.3E-03 (2.1E-02) | 1.5E-02 (4.8E-02) | 0.621 |
| **Metabolites in Cohort 2** | | | | |
| Number | 5 | 38 | 7 | *p*-value |
| Pramipexole | 0.83 (0.46) | 1.84 (4.0) | 0.87 (1.10) | 0.71 |
| 3-deoxysappanchalcone | 6.93 (13.86) b | 0.96 (2.33) a | 3.08 (6.34) | 0.046 |
| 1,3-Dimethyluracil | 1.19 (0.68) | 7.31 (16.1) | 3.64 (4.61) | 0.595 |
| Histidylmethionine | 3.83 (5.17) | 4.94 (8.45) | 1.87 (2.24) | 0.618 |

| Prolylproline | 1.51 (0.24) | 2.00 (2.27) | 1.43 (0.56) | 0.736 |
| --- | --- | --- | --- | --- |
| Droxidopa | 2.05 (2.71) | 0.75 (1.34) | 0.66 (0.99) | 0.178 |
| Epinephrine glucuronide | 3.78 (6.49) | 5.00 (9.60) | 1.52 (1.91) | 0.621 |
| 18-methylnonadecanoic acid | 4.41 (6.23) | 1.78 (1.67) | 2.67 (3.36) | 0.106 |
| N-Acetylisoleucine | 2.70 (0.52) | 4.93 (7.28) | 2.94 (2.55) | 0.627 |
| Cer(d18:1/18:1(11Z)) | 2.93 (3.51) | 4.81 (6.78) | 2.94 (2.73) | 0.660 |
| Leucine | 1.35 (0.57) | 3.81 (6.26) | 1.48 (2.56) | 0.585 |
| Tyrosyl-Threonine | 3.09 (4.12) | 4.61 (7.92) | 1.68 (2.36) | 0.585 |
| LysoPC(14:0/0:0) | 0.87 (0.48) | 1.15 (2.05) | 0.83 (1.39) | 0.894 |
| Ascorbic acid | 2.86 (2.33) | 1.53 (1.27) | 1.50 (1.11) | 0.135 |
| **Metabolites in Cohort 3** | | | | |
| Number | 5 | 25 | 9 |  |
| Docosenic acid | 344471 (223403) | 287043 (167018) | 270261 (214345) | 0.765 |
| 25-Azacholesterol | 332444 (18600) | 274310 (99131) | 249185 (112334) | 0.314 |
| dodec-6-enoic acid | 158542 (243068) | 77366 (71937) | 116448 (147172) | 0.351 |
| Deoxycytidine monophosphate | 503090 (204941) c b | 258872 (233707) a | 186255 (164983) a | 0.038 |
| Octadecenoic acid | 148102 (86832) | 12182 (51508) | 155797 (44660) | 0.246 |
| 4-Trimethylammoniobutanoic acid | 2207 (1354) | 25283 (108837) | 61395 (172163) | 0.636 |
| Glutamylproline | 39171 (10028) | 31524 (13066) | 49968 (33273) | 0.058 |
| Bilirubin | 15271 (3829) | 19678 (13553) | 16543 (12406) | 0.685 |
| p-Cresol | 42321 (18214) | 41342 (17541) | 38549 (10439) | 0.880 |
| N-butyl Oleate | 82258 (19491) | 70777 (27139) | 67733 (35634) | 0.647 |
| 4-hydroxyundecanoic acid | 42989 (16200) | 32602 (21349) | 31065 (25807) | 0.585 |
| Nicotinamide ribotide | 47834 (20705) | 39178 (17894) | 38697 (14006) | 0.597 |
| 7-methyldecanoic acid | 2649 (1696) c | 3759 (3197) c | 10000 (12106) a, b | 0.036 |

Continuous variables were evaluated for normality using the Kolmogorov–Smirnov test. Data are presented as mean (standard deviation). The p- values for continuous variables derived from One-way ANOVA, and post hoc comparisons were adjusted by LSD.

a: *p* values are given overall among Dyskinesia, Symptom fluctuation and Combined group. a Dyskinesia b Symptom fluctuation c Combined group *p-value*<0.05

**Supple table 19: Analysis of core microbial differences between PD-MC and PD-NMC groups using a generalized linear model.**

| **Characteristics** | **beta** | **95% Wald confidence interval** | **Wald χ²** | ***p* value** |
| --- | --- | --- | --- | --- |
| Age | -0.027 | (-0.15, 0.10) | 0.19 | 0.664 |
| Sex (Female)a | -0.464 | (-2.54, 1.61) | 0.19 | 0.660 |
| BMI | -0.206 | (-0.54, 0.13) | 1.45 | 0.229 |
| Education | 0.101 | (-0.22, 0.42) | 0.38 | 0.538 |
| Disease duration | -0.476 | (-0.79, -0.17) | 8.98 | 0.003 |
| Constipation a | -0.363 | (-2.48, 1.75) | 0.113 | 0.737 |
| MMSE | 0.018 | (-0.17, 0.21) | 0.036 | 0.849 |
| HAMA | -0.220 | (-0.52, 0.08) | 2.11 | 0.146 |
| HAMD | 0.176 | (-0.19, 0.54) | 0.88 | 0.349 |
| RBDQ-HK | 0.079 | (0.01, 0.15) | 4.72 | 0.030 |

| LEED | -0.007 | (-0.015, 0.000) | 3.76 | 0.053 |
| --- | --- | --- | --- | --- |
| Lactobacillus | 702.2 | (-1129.2, 2533.6) | 0.57 | 0.452 |
| Agathobacter | 323.9 | (-49.6, 697.5) | 2.89 | 0.089 |
| Ligilactobacillus | -960.2 | (-1782.6, -137.86) | 5.24 | 0.022 |
| Limosilactobacillus | 2914.2 | (535.9, 5292.4) | 5.77 | 0.016 |
| Bifidobacterium | 9.494 | (-18.5, 37.5) | 0.44 | 0.507 |
| Lactobacillus* LEEDb | -2.531 | (-6.91, 1.85) | 1.29 | 0.257 |
| Agathobacter* LEEDb | -0.375 | (-0.89, 0.14) | 2.01 | 0.157 |
| Ligilactobacillus* LEEDb | 1.934 | (0.23, 3.64) | 4.94 | 0.026 |
| Limosilactobacillus* LEEDb | -2.712 | (-5.03, -0.39) | 5.24 | 0.022 |
| Bifidobacterium* LEEDb | -0.013 | (-0.08, 0.054) | 0.14 | 0.704 |
| Lactobacillus* RBDc | 32.504 | (-43.27, 108.27) | 0.71 | 0.400 |
| Agathobacter* RBDc | -5.899 | (-15.98, 4.18) | 1.32 | 0.251 |
| Ligilactobacillus* RBDc | -0.02 | (-14.34, 14.30) | 0.000 | 0.998 |
| Limosilactobacillus* RBDc | 73.66 | (-130.66, -16.66) | 6.42 | 0.011 |
| Bifidobacterium* RBDc | -0.088 | (-0.64, 0.47) | 0.095 | 0.758 |

The results of the generalized Linear Model (GLM) corrected the basic demographic information (age, gender, BMI, education, disease duration), the non-motor symptoms (MMSE, HAMA, HAMD, RBD, constipation) and LEED to conduct a differential analysis of the five core microbiota， showing that the significance of Omnibus was p <0.01. The Wald square values (χ² , along with their corresponding 95% confidence intervals (95% CI), were presented in the table. Statistical significance was determined at a level of p < 0.05. The analysis was conducted using data from a sample of N = 92 participants with PD in Cohort 1.

a: The categorical variable codes are as follows: Gender (Gender: male = reference group;) Female; Constipation (Constipation: No constipation = reference group;) Constipation. The results of the generalized Linear Model (GLM) showed that the significance of Omnibus was p <0.01.

b: The interaction effect between core microbiota and LEED.

c: The interaction effect between core microbiota and RBD.
