# Supplementary figures and images for: Exploring the Fecal Microbiome Dysbiosis and Its Plasma Metabolome Determinants in Advanced Parkinson's Disease With Motor Complications
Source: CNS Neurosci Ther. 2026 Jan 23;32(1):e70750. doi: 10.1002/cns.70750 (PMC12828341; doi:10.1002/cns.70750)

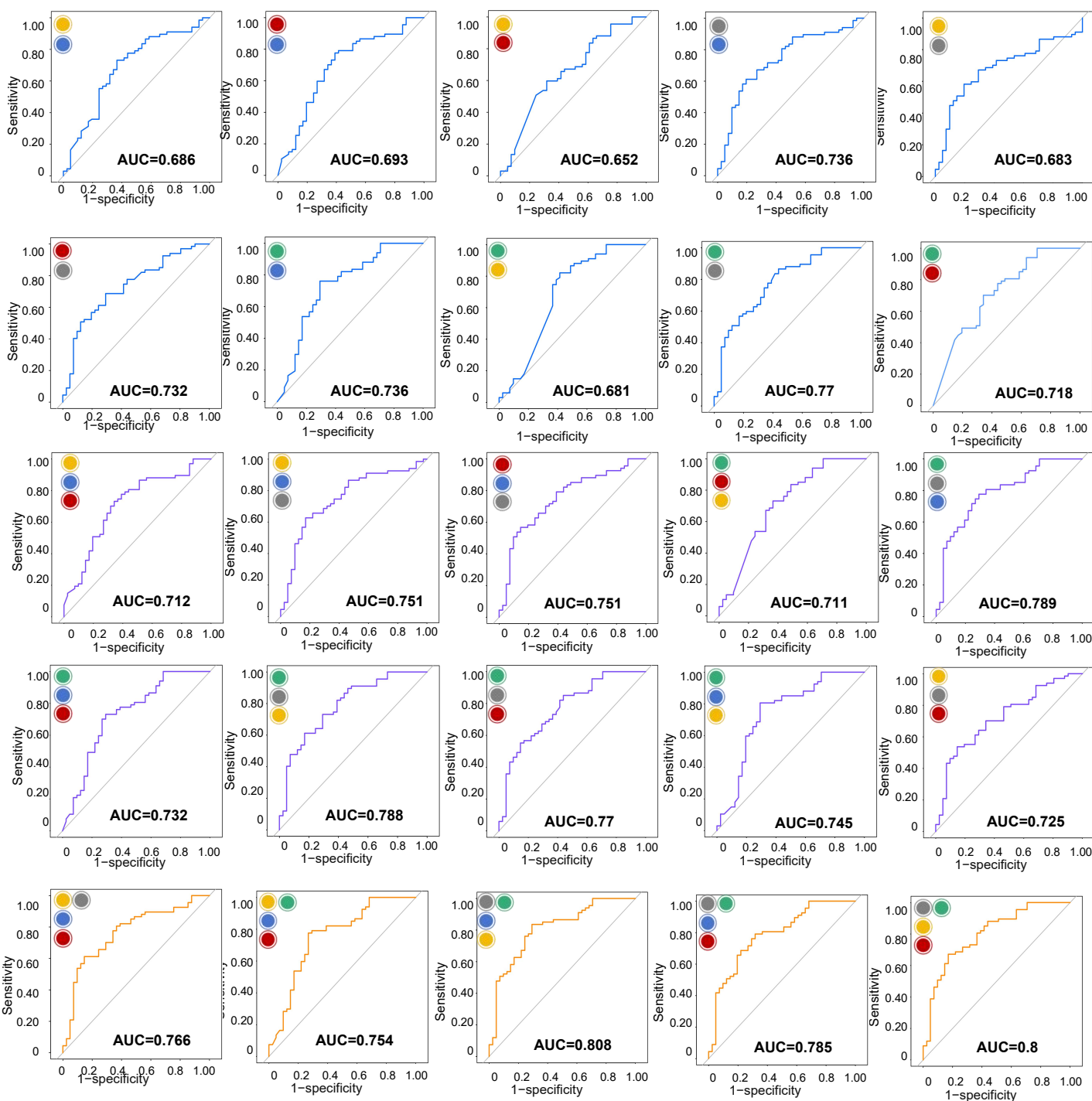

Supple Figure 1

Supplement: Supplementary file 1 — Figure S1: Differential microbiota correlation in PD‐NMC groups. Spearman correlation analysis of the characteristic microbial abundances, as depicted in Figure 3e, revealed a significant association among the microbiota in PD‐NMC. The phylus levels are represented by nodes of different colors, while the edges indicated positive (red) or negative (blue) correlations. The thickness of the edges corresponds to the magnitude of the correlation coefficients; All correlation p values are below 0.05. Detailed correlation coefficients and p values are provided in the right heat map. Figure S2: Combined AUC of Cohort 1 potential microbiota. In Cohort 1, combined AUC analysis of the two, three and four core metabolites is illustrated in Figure S1, related to Figure 4. Figure S3: Integrated analysis of multiomics in PD‐MC. (a) Venn diagrams showed PD‐MC participants overlap in Cohorts 1, 2 and 3, revealed shared PD‐MC patients in Cohorts 1 and 2 (N = 7) and in Cohorts 1 and 3 (N = 10). (b) The network analysis demonstrated statistically significant and suggestive associations (p < 0.05, Spearman analysis) among differentially abundant microbiota taxa (illustrated in red) and metabolites (illustrated in blue). Edges connecting the nodes represent positive (red) or negative (blue) correlations. Node numbers corresponding to microorganisms and metabolites are provided in the accompanying legend, with core elements emphasized in red and blue. (c) Correlation chord diagram of microorganisms and metabolites in PD‐MC. (d) Core Microbiota–Metabolites correlation heat map; p < 0.05*, p < 0.01**, p < 0.001***. Figure S4: Integrated analysis of multiomics in PD patients. (a) Venn diagrams showed PD participants overlap in Cohorts 1, 2 and 3, revealed shared PD patients in Cohorts 1 and 2 (N = 16) and in Cohorts 1 and 3 (N = 21). (b) The network analysis demonstrated statistically significant and suggestive associations (p < 0.05, Spearman analysis) among differentially abundant taxa (il [file CNS-32-e70750-s001.zip › Supple_documentary/Supple_Figures1.pdf]

Spearman correlation in PD-NMC

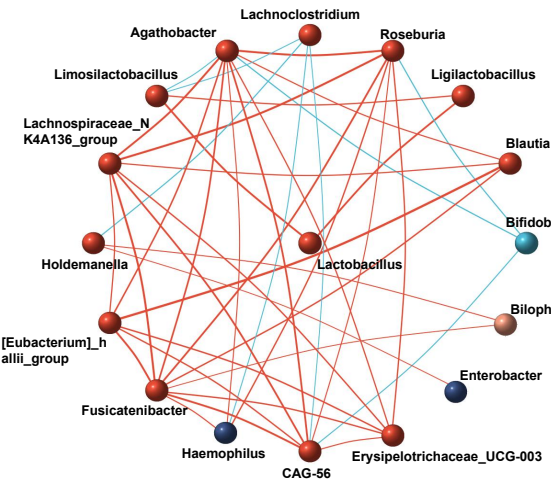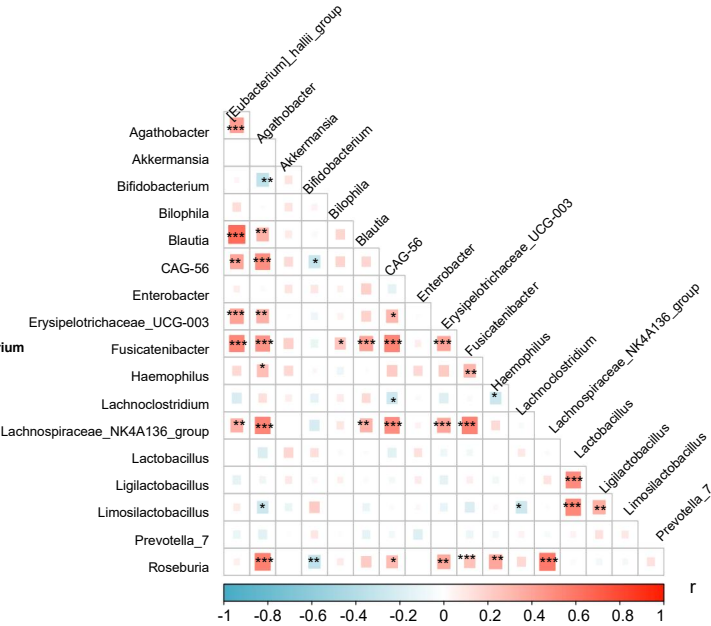

Supple Figure 2

Supplement: Supplementary file 1 — Figure S1: Differential microbiota correlation in PD‐NMC groups. Spearman correlation analysis of the characteristic microbial abundances, as depicted in Figure 3e, revealed a significant association among the microbiota in PD‐NMC. The phylus levels are represented by nodes of different colors, while the edges indicated positive (red) or negative (blue) correlations. The thickness of the edges corresponds to the magnitude of the correlation coefficients; All correlation p values are below 0.05. Detailed correlation coefficients and p values are provided in the right heat map. Figure S2: Combined AUC of Cohort 1 potential microbiota. In Cohort 1, combined AUC analysis of the two, three and four core metabolites is illustrated in Figure S1, related to Figure 4. Figure S3: Integrated analysis of multiomics in PD‐MC. (a) Venn diagrams showed PD‐MC participants overlap in Cohorts 1, 2 and 3, revealed shared PD‐MC patients in Cohorts 1 and 2 (N = 7) and in Cohorts 1 and 3 (N = 10). (b) The network analysis demonstrated statistically significant and suggestive associations (p < 0.05, Spearman analysis) among differentially abundant microbiota taxa (illustrated in red) and metabolites (illustrated in blue). Edges connecting the nodes represent positive (red) or negative (blue) correlations. Node numbers corresponding to microorganisms and metabolites are provided in the accompanying legend, with core elements emphasized in red and blue. (c) Correlation chord diagram of microorganisms and metabolites in PD‐MC. (d) Core Microbiota–Metabolites correlation heat map; p < 0.05*, p < 0.01**, p < 0.001***. Figure S4: Integrated analysis of multiomics in PD patients. (a) Venn diagrams showed PD participants overlap in Cohorts 1, 2 and 3, revealed shared PD patients in Cohorts 1 and 2 (N = 16) and in Cohorts 1 and 3 (N = 21). (b) The network analysis demonstrated statistically significant and suggestive associations (p < 0.05, Spearman analysis) among differentially abundant taxa (il [file CNS-32-e70750-s001.zip › Supple_documentary/Supple_Figures2.pdf]

Spearman correlation between Microbiota and Metabolites in PD-MC

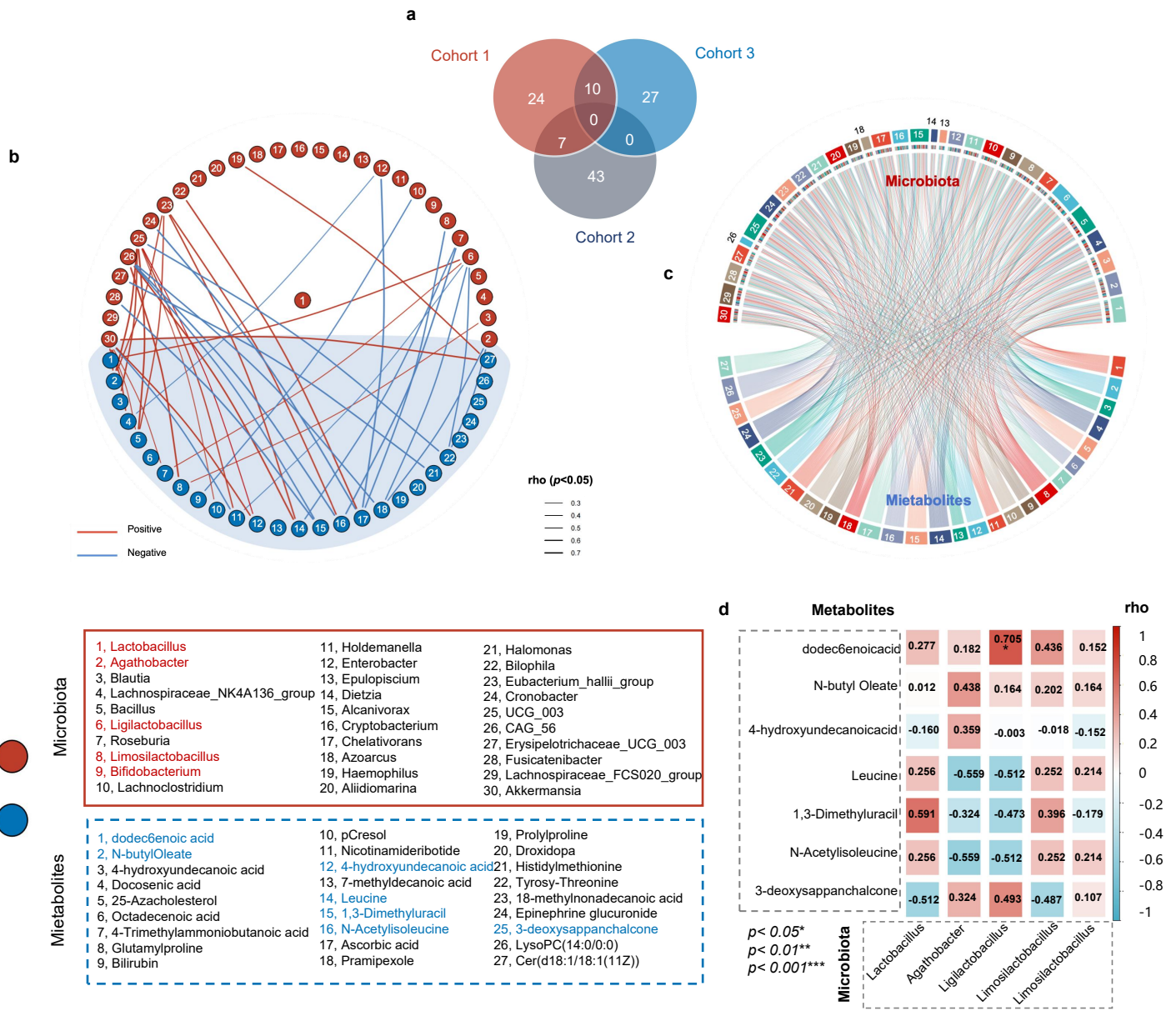

Supple Figure 3

Supplement: Supplementary file 1 — Figure S1: Differential microbiota correlation in PD‐NMC groups. Spearman correlation analysis of the characteristic microbial abundances, as depicted in Figure 3e, revealed a significant association among the microbiota in PD‐NMC. The phylus levels are represented by nodes of different colors, while the edges indicated positive (red) or negative (blue) correlations. The thickness of the edges corresponds to the magnitude of the correlation coefficients; All correlation p values are below 0.05. Detailed correlation coefficients and p values are provided in the right heat map. Figure S2: Combined AUC of Cohort 1 potential microbiota. In Cohort 1, combined AUC analysis of the two, three and four core metabolites is illustrated in Figure S1, related to Figure 4. Figure S3: Integrated analysis of multiomics in PD‐MC. (a) Venn diagrams showed PD‐MC participants overlap in Cohorts 1, 2 and 3, revealed shared PD‐MC patients in Cohorts 1 and 2 (N = 7) and in Cohorts 1 and 3 (N = 10). (b) The network analysis demonstrated statistically significant and suggestive associations (p < 0.05, Spearman analysis) among differentially abundant microbiota taxa (illustrated in red) and metabolites (illustrated in blue). Edges connecting the nodes represent positive (red) or negative (blue) correlations. Node numbers corresponding to microorganisms and metabolites are provided in the accompanying legend, with core elements emphasized in red and blue. (c) Correlation chord diagram of microorganisms and metabolites in PD‐MC. (d) Core Microbiota–Metabolites correlation heat map; p < 0.05*, p < 0.01**, p < 0.001***. Figure S4: Integrated analysis of multiomics in PD patients. (a) Venn diagrams showed PD participants overlap in Cohorts 1, 2 and 3, revealed shared PD patients in Cohorts 1 and 2 (N = 16) and in Cohorts 1 and 3 (N = 21). (b) The network analysis demonstrated statistically significant and suggestive associations (p < 0.05, Spearman analysis) among differentially abundant taxa (il [file CNS-32-e70750-s001.zip › Supple_documentary/Supple_Figures3.pdf]

Spearman correlation between microbiota and metabolites in PD patients

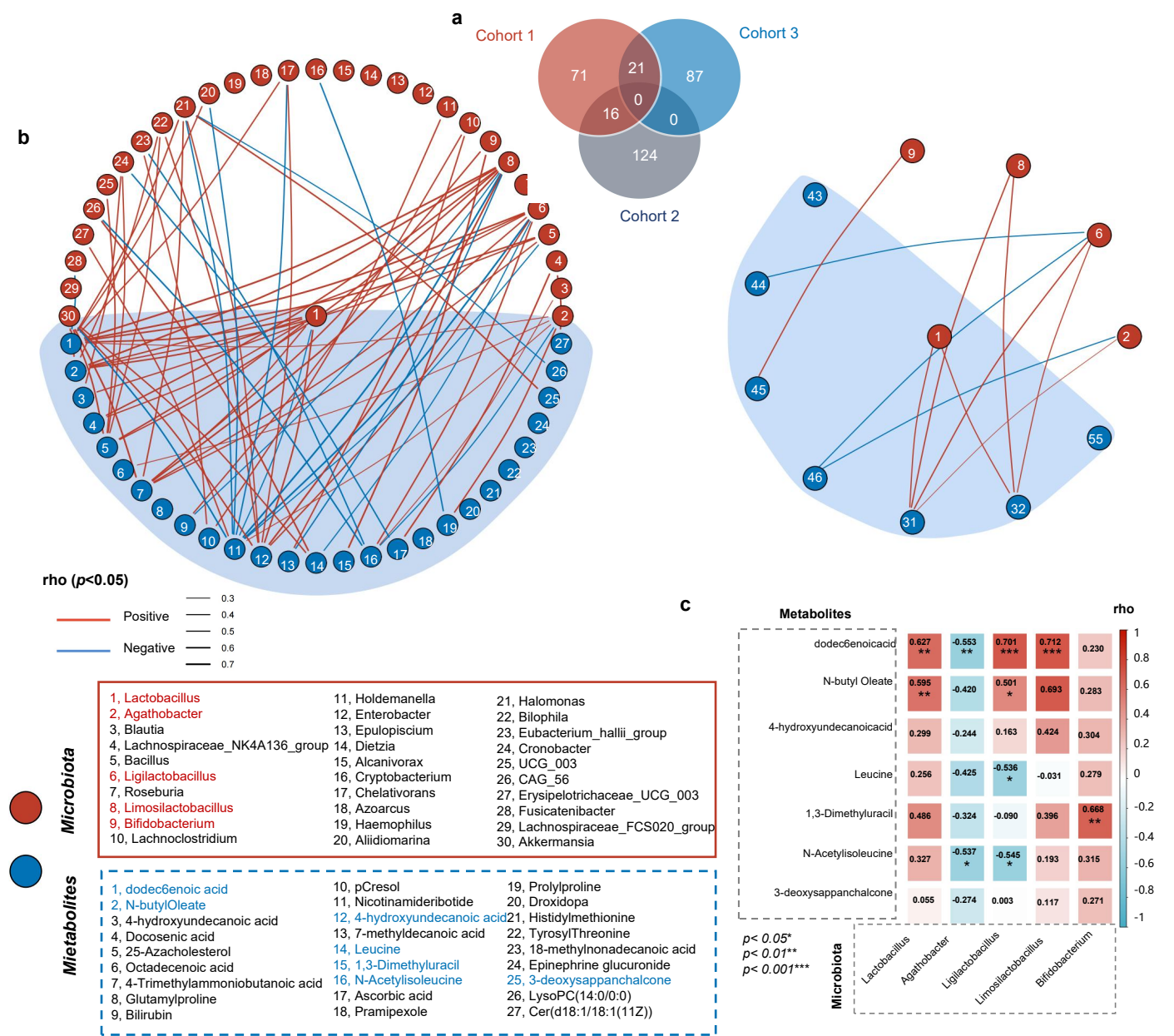

Supple Figure 4

Supplement: Supplementary file 1 — Figure S1: Differential microbiota correlation in PD‐NMC groups. Spearman correlation analysis of the characteristic microbial abundances, as depicted in Figure 3e, revealed a significant association among the microbiota in PD‐NMC. The phylus levels are represented by nodes of different colors, while the edges indicated positive (red) or negative (blue) correlations. The thickness of the edges corresponds to the magnitude of the correlation coefficients; All correlation p values are below 0.05. Detailed correlation coefficients and p values are provided in the right heat map. Figure S2: Combined AUC of Cohort 1 potential microbiota. In Cohort 1, combined AUC analysis of the two, three and four core metabolites is illustrated in Figure S1, related to Figure 4. Figure S3: Integrated analysis of multiomics in PD‐MC. (a) Venn diagrams showed PD‐MC participants overlap in Cohorts 1, 2 and 3, revealed shared PD‐MC patients in Cohorts 1 and 2 (N = 7) and in Cohorts 1 and 3 (N = 10). (b) The network analysis demonstrated statistically significant and suggestive associations (p < 0.05, Spearman analysis) among differentially abundant microbiota taxa (illustrated in red) and metabolites (illustrated in blue). Edges connecting the nodes represent positive (red) or negative (blue) correlations. Node numbers corresponding to microorganisms and metabolites are provided in the accompanying legend, with core elements emphasized in red and blue. (c) Correlation chord diagram of microorganisms and metabolites in PD‐MC. (d) Core Microbiota–Metabolites correlation heat map; p < 0.05*, p < 0.01**, p < 0.001***. Figure S4: Integrated analysis of multiomics in PD patients. (a) Venn diagrams showed PD participants overlap in Cohorts 1, 2 and 3, revealed shared PD patients in Cohorts 1 and 2 (N = 16) and in Cohorts 1 and 3 (N = 21). (b) The network analysis demonstrated statistically significant and suggestive associations (p < 0.05, Spearman analysis) among differentially abundant taxa (il [file CNS-32-e70750-s001.zip › Supple_documentary/Supple_Figures4.pdf]

Workflow for integrated microbiome-metabolite analysis

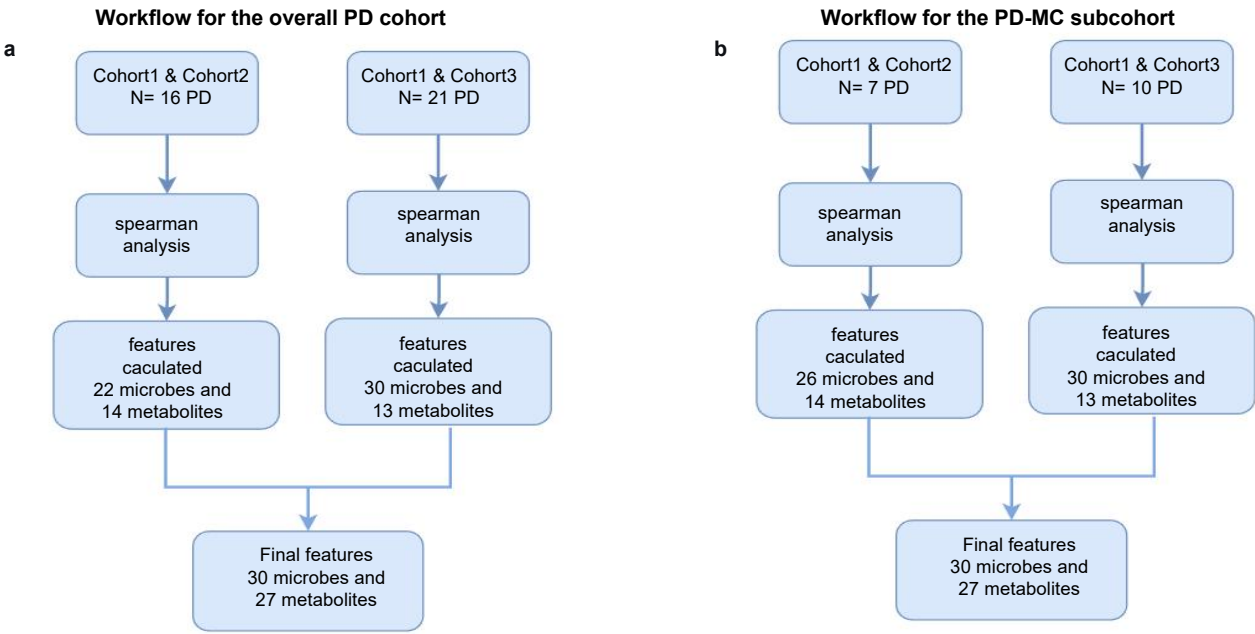

Supplement: Supplementary file 1 — Figure S1: Differential microbiota correlation in PD‐NMC groups. Spearman correlation analysis of the characteristic microbial abundances, as depicted in Figure 3e, revealed a significant association among the microbiota in PD‐NMC. The phylus levels are represented by nodes of different colors, while the edges indicated positive (red) or negative (blue) correlations. The thickness of the edges corresponds to the magnitude of the correlation coefficients; All correlation p values are below 0.05. Detailed correlation coefficients and p values are provided in the right heat map. Figure S2: Combined AUC of Cohort 1 potential microbiota. In Cohort 1, combined AUC analysis of the two, three and four core metabolites is illustrated in Figure S1, related to Figure 4. Figure S3: Integrated analysis of multiomics in PD‐MC. (a) Venn diagrams showed PD‐MC participants overlap in Cohorts 1, 2 and 3, revealed shared PD‐MC patients in Cohorts 1 and 2 (N = 7) and in Cohorts 1 and 3 (N = 10). (b) The network analysis demonstrated statistically significant and suggestive associations (p < 0.05, Spearman analysis) among differentially abundant microbiota taxa (illustrated in red) and metabolites (illustrated in blue). Edges connecting the nodes represent positive (red) or negative (blue) correlations. Node numbers corresponding to microorganisms and metabolites are provided in the accompanying legend, with core elements emphasized in red and blue. (c) Correlation chord diagram of microorganisms and metabolites in PD‐MC. (d) Core Microbiota–Metabolites correlation heat map; p < 0.05*, p < 0.01**, p < 0.001***. Figure S4: Integrated analysis of multiomics in PD patients. (a) Venn diagrams showed PD participants overlap in Cohorts 1, 2 and 3, revealed shared PD patients in Cohorts 1 and 2 (N = 16) and in Cohorts 1 and 3 (N = 21). (b) The network analysis demonstrated statistically significant and suggestive associations (p < 0.05, Spearman analysis) among differentially abundant taxa (il [file CNS-32-e70750-s001.zip › Supple_documentary/Supple_Figures5.pdf]
